# Targeting Inflammation by Pioglitazone and its R-Enantiomer Mitigates Pathological Myocardial Remodeling in Murine Hypertrophic Cardiomyopathy

**DOI:** 10.1016/j.jacbts.2026.101574

**Published:** 2026-06-13

**Authors:** Anna-Theresa Pfaller, Claudia Veneziano, Sarala Raj Murthi, Jan B. Stöckl, Bachuki Shashikadze, Florian Flenkenthaler, Josh Gorham, Alessandra Moretti, Ana Kitanovic, Linden J. Gearing, Frederik Heinrich, Pawel Durek, Katrin Lehmann, Mir-Farzin Mashreghi, Peter Ewert, Thomas Fröhlich, Joachim P. Schmitt, Nadine Spielmann, Martin Hrabě de Angelis, Manuel Schmid, Christopher N. Toepfer, Eicke Latz, Karin Klingel, Gianluca Santamaria, Jonathan G. Seidman, Christine E. Seidman, Cordula M. Wolf

**Affiliations:** a Department of Congenital Heart Defects and Pediatric Cardiology, German Heart Centre Munich, Technical University of Munich, School of Medicine and Health, Munich, Germany; b DZHK (German Centre for Cardiovascular Research), partner site Munich Heart Alliance, Munich, Germany; c Department of Experimental and Clinical Medicine, “Magna Graecia” University of Catanzaro, Catanzaro, Italy; d Laboratory for Functional Genome Analysis LAFUGA, Gene Center, LMU Munich, Munich, Germany; e Department of Genetics, Harvard Medical School, Boston, Massachusetts, USA; f First Department of Medicine and Regenerative Medicine in Cardiovascular Diseases, Klinikum Rechts der Isar, School of Medicine and Health, Technical University of Munich, Germany; g German Rheumatism Research Centre, Berlin, Germany; h Zuse Institute Berlin, Berlin, Germany; i Institute of Pharmacology, University Hospital Düsseldorf and Cardiovascular Research Institute Düsseldorf (CARID), Heinrich-Heine-University, Düsseldorf, Germany; j Institute of Experimental Genetics and German Mouse Clinic, Helmholtz Center Munich, German Research Center for Environmental Health, Neuherberg, Germany; k Cardiovascular Medicine, Radcliffe Department of Medicine, University of Oxford, Oxford, United Kingdom; l Wellcome Centre for Human Genetics, University of Oxford, Oxford, United Kingdom; m Cardiopathology, Institute for Pathology and Neuropathology, University Hospital of Tübingen, Tübingen, Germany; n Interdepartmental Center of Services (CIS), Omics Sciences and Biobank, "Magna Graecia" University, Catanzaro, Italy

**Keywords:** hypertrophic cardiomyopathy, inflammation and oxidative stress, mitochondrial dysfunction, myocardial hypertrophy and fibrosis, pioglitazone

## Abstract

Hypertrophic cardiomyopathy (HCM) is driven by sarcomeric mutations that cause energetic failure and secondary inflammation. This study demonstrates that targeting this metabolic-inflammatory axis with pioglitazone or its peroxisome proliferator-activated receptor gamma inactive enantiomer, R-pioglitazone, reverses disease progression in a murine HCM model. Both agents restored mitochondrial function (including Mitochondrial Pyruvate Carrier 1 [*MPC1*] levels) and resolved inflammation. Notably, R-pioglitazone showed superior efficacy, reducing interstitial fibrosis by >95% and hypertrophy by 33% without affecting healthy control hearts. These findings identify R-pioglitazone as a promising, mechanism-based candidate for disease-modifying therapy in HCM.

## Highlights

• Energetic, inflammatory, and metabolic dysregulation are key drivers of disease progression in HCM.
• Targeted modulation of these pathways ameliorates hypertrophy and fibrosis in an HCM mouse model.
• PPARγ-independent metabolic intervention of R-pio represents a promising strategy to counteract upstream drivers of HCM remodeling.

## Visual Abstract

**Figure:**
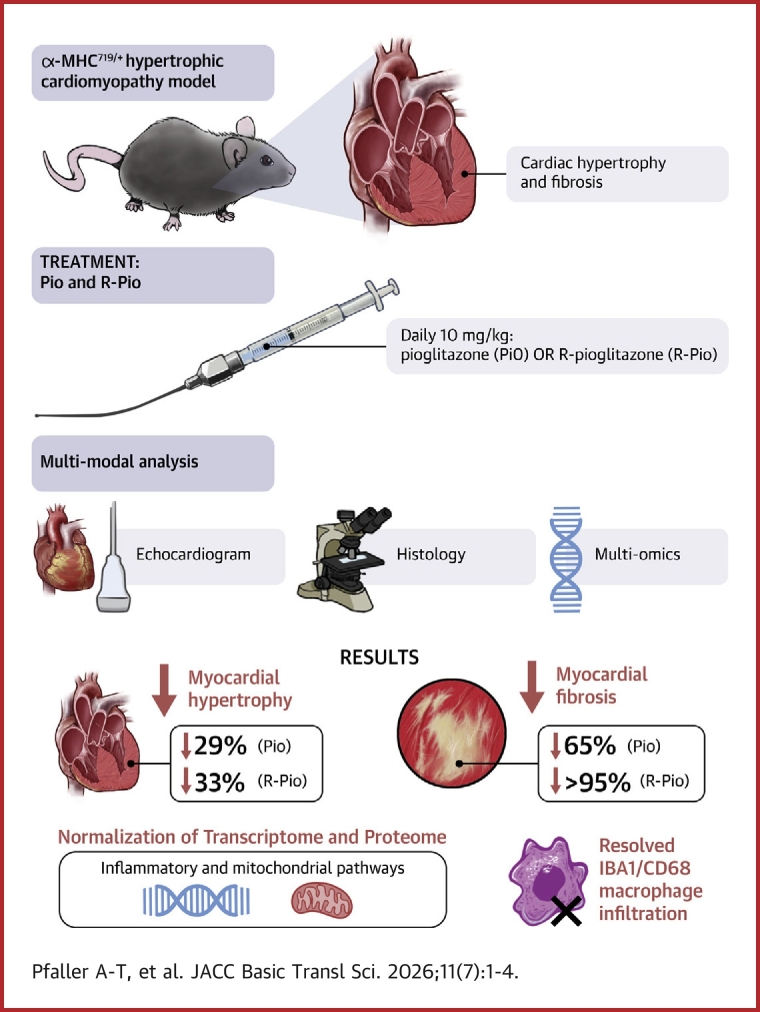

Hypertrophic cardiomyopathy (HCM) affects approximately 1 in 500 individuals.^1^^,^^2^ It is the most common inherited cardiac disease, and a leading cause of sudden cardiac death in young individuals.^3^^,^^4^ Clinically, HCM is characterized by progressive myocardial hypertrophy, diastolic and systolic dysfunction, and an increased risk of life-threatening arrhythmias. Histologically, it is defined by cardiomyocyte hypertrophy, interstitial fibrosis, and myofiber disarray.^2^^,^^5^

HCM is inherited as an autosomal-dominant trait. While pathogenic variants in genes encoding sarcomeric proteins are identified in approximately 40% of patients (sarcomere-positive HCM), a significant proportion of cases remain genotype-negative, sharing a similar phenotype driven by complex polygenic or environmental factors.^6^^,^^7^ Among the identified causes, alterations in *MYH7* (β-myosin heavy chain) and *MYBPC3* (cardiac myosin-binding protein C) are the most frequent.^8^ In sarcomere-positive cases, alterations alter biophysics, including abnormal myosin-actin interactions and elevated ATPase activity. These alterations result in hypercontractility and impaired relaxation, which impose a chronic energetic burden on the cardiomyocyte.^5^^,^^9^

Emerging evidence suggests that this "mechano-energetic uncoupling" is a primary driver of disease progression. The elevated ATP demand, driven by excessive contractility and calcium cycling, leads to early energy depletion and mitochondrial dysfunction.^10^, ^11^, ^12^ This metabolic stress compromises antioxidant capacity and increases mitochondrial reactive oxygen species (ROS) production. Accumulating data indicate that this oxidative stress serves as a potent trigger for downstream profibrotic and proinflammatory signaling pathways, thereby linking the initial metabolic deficit to the structural remodeling observed in HCM.^13^^,^^14^ Consequently, energy deficiency and mitochondrial dysfunction are likely upstream drivers that fuel secondary inflammation and fibrosis.

Although these upstream mechanisms are increasingly acknowledged, current therapeutic strategies remain predominantly centered on symptom management. Although the myosin inhibitor Mavacamten represents an important advance for obstructive HCM,^15^ its efficacy in nonobstructive phenotypes is less established, as recently illustrated by the modest outcomes of the ODYSSEY-HCM (A Study of Mavacamten in Nonobstructive Hypertrophic Cardiomyopathy) trial.^16^ Preventive strategies such as avoidance of competitive sports and prophylactic implantable cardioverter-defibrillator implantation are primarily reserved for high-risk individuals. In later disease stages, patients may require symptomatic treatment of heart failure or ultimately heart transplantation.^17^^,^^18^ These limitations underscore the need for novel therapeutic approaches addressing the underlying cellular and metabolic drivers of HCM.

Pharmacological agents capable of modulating mitochondrial function and inflammation are therefore of particular interest. Pioglitazone (pio), a thiazolidinedione and peroxisome proliferator-activated receptor gamma (PPARγ) agonist approved for type 2 diabetes, has demonstrated antifibrotic and anti-inflammatory effects in various preclinical models, including nonalcoholic steatohepatitis,^19^^,^^20^ cardiac disease,^21^ pulmonary fibrosis,^22^ and renal fibrosis,^23^ partly through modulation of mitochondrial function and inflammatory signaling. Pio has been identified as a selective inhibitor of long-chain acyl-CoA synthetase 4 (*ACSL4*), suppressing its enzymatic activity and thereby reducing lipid peroxidation and ferroptotic cell death.^24^ In addition, its mitochondria-stabilizing effects have been associated with modulation of the mitochondrial pyruvate carrier (*MPC*), a heterodimeric complex composed of *MPC1* and *MPC2*, which regulates pyruvate uptake into mitochondria and thereby controls oxidative metabolism and cellular energy homeostasis.^25^, ^26^, ^27^ However, its clinical use is limited by PPARγ-associated side effects such as weight gain, fluid retention, and bone loss.^28^ Pio exists as a racemic mixture comprising R- and S-enantiomers, with the R-enantiomer (R-pio) preserving the beneficial anti-inflammatory and mitochondria-stabilizing effects independent of PPARγ-activation,^26^^,^^29^ potentially conferring an improved safety profile.

In this study, we evaluated the therapeutic potential of pio and its R-pio as disease-modifying agents in HCM, using a well-established murine model carrying the Arg719Trp alteration in the myosin heavy chain 6 gene.^30^

## Methods

### Chemicals

All chemicals were from Sigma-Aldrich unless stated differently.

### HCM mouse model

All animal procedures were conducted in accordance with European Directive 2010/63/EU and were approved by the local animal ethics committee (Government of Upper Bavaria, Munich, Germany; Project Number: 55.2-2532.Vet_02-15-242).

The previously described α-MHC^719/+^ mouse model, which carries the common human HCM allelic variant Arg719Trp in the murine *Myh6* gene was used in this study.^30^ These heterozygous mice develop characteristic features of HCM, including myocardial hypertrophy and fibrosis, by approximately 30 weeks of age. The α-MHC^719/+^ mouse line was obtained through collaboration with the laboratory of Prof Joachim Schmitt (Heinrich Heine University Düsseldorf), a center of expertise in cardiovascular pharmacology.

To accelerate disease onset and enable early-stage analysis, cyclosporine A (15 mg/kg body weight, dissolved in phosphate buffer saline) was administered orally twice daily for 6 weeks, starting between 6 and 8 weeks of age. This treatment induces a robust HCM phenotype in α-MHC^719/+^ mice, with histologically and functionally detectable myocardial remodeling by 12 weeks of age. Importantly, cyclosporine A by itself does not result in a cardiomyopathic phenotype in wild-type (WT) mice.^31^

To reduce biological variability, only male mice were included, given that the HCM phenotype manifests more predominantly in male mice. Age- and sex-matched untreated WT littermates on the same 129/SvEv genetic background served as controls.

Pioglitazone and R-pio were administered at 10 mg/kg/d by oral gavage. Using body surface area–based dose conversion (K_m mouse = 3; K_m human = 37),^32^ this corresponds to a human-equivalent dose of 0.81 mg/kg (≈48.6 mg/d for a 60-kg adult), which is close to the clinically used maximum dose of pio (45 mg/d).^33^

Animals were randomized into the following age-matched treatment groups:

WT + vehicle (WT), α-MHC^719/+^ + vehicle (α-MHC^719/+^), α-MHC^719/+^ + pioglitazone (α-MHC^719/+^ + pio), and α-MHC^719/+^ + R-pioglitazone (α-MHC^719/+^ + R-pio).

All groups received cyclosporine A treatment as described in the previous text.

At the end of the experimental protocol, animals were anesthetized with isoflurane and weighed under deep sedation. Euthanasia was subsequently carried out via cervical dislocation in accordance with institutional and national ethical guidelines. Following confirmation of death, a midline thoracotomy was performed to expose the thoracic cavity, and hearts were carefully excised. The hearts were rinsed in phosphate buffer saline at a temperature of 4 °C, which consisted of 140 mmol/L NaCl, 3 mmol/L KCl, 6.5 mmol/L Na2HPO4, 1.5 mmol/L KH2PO4, with a pH of 7.4.

### Echocardiography

Transthoracic echocardiography was performed on unsedated mice at the final day of the experimental protocol using a Vevo 3100 Imaging System (VisualSonics), as previously described.^34^ The imaging was carried out with a linear-array probe (MX400) ranging from 18 to 38 MHz. The imaging included 2-dimensional views (left parasternal long- and short-axis) as well as M-Mode (left parasternal short-axis) images. Based on M-Mode tracings, measurements were obtained for the left ventricular end-diastolic diameter, left ventricular end-systolic diameter, and maximal left ventricular wall thickness. The average values from 3 consecutive cardiac cycles are reported. Echocardiographic measurements were obtained by an experienced observer blinded to the genotype of the mice.

### Histopathology

Left ventricular tissue was fixed overnight in 4% paraformaldehyde. Following fixation, tissue was processed and embedded in paraffin according to a previously described protocol.^34^

### Quantification of myocardial fibrosis

Embedded hearts were serially sectioned every 4 μm from base to apex in a transverse plane. Adjacent sections were stained with Masson’s trichrome to assess collagen deposition resulting from fibrosis. Fibrosis quantification was performed using ImageJ software on 3 representative transverse sections per heart.

### Quantification of myocardial macrophage infiltration

Embedded hearts were sectioned at a thickness of 2 μm in the transverse plane. Sections were deparaffinized, rehydrated, and subjected to antigen retrieval according to standard protocols. Immunohistochemical staining was performed using primary antibodies against CD68 (ab125212, Abcam) and Iba1 (ab178846, Abcam) to assess myocardial macrophage infiltration. Staining was visualized using standard chromogenic detection methods. The number of positively stained cells was manually counted on 2 representative transverse sections per heart at 20× magnification.

### RNA sequencing

For transcriptomic analysis, left ventricular tissue was collected from WT and α-MHC^719/+^ mice with or without treatment. In the pio cohort, RNA was extracted from pooled samples (n = 6 WT, n = 4 α-MHC^719/+^, n = 7 α-MHC^719/+^+ pio; all hearts per group were pooled into 1 sample). In the R-pio cohort, RNA was extracted from individual hearts (n = 3 WT, n = 2 α-MHC^719/+^, n = 2 α-MHC^719/+^+ R-pio). All samples were processed, normalized, and analyzed together as detailed in the following sections.

Strand-specific, polyA-enriched RNA sequencing was performed as previously described.^35^ Briefly, RNA was isolated from whole-cell lysates using the AllPrep RNA Kit (Qiagen) and RNA integrity number (RIN) was determined with the Agilent 2100 BioAnalyzer (RNA 6000 Nano Kit, Agilent). For library preparation, one μg of RNA was poly (A) selected, fragmented, and reverse transcribed with the Elute, Prime, and Fragment Mix (Illumina). A-tailing, adaptor ligation, and library enrichment were performed as described in the TruSeq Stranded mRNA Sample Prep Guide (Illumina). RNA libraries were assessed for quality and quantity with the Agilent 2100 BioAnalyzer and the Quant-iT PicoGreen dsDNA Assay Kit (Life Technologies). RNA libraries were sequenced as 100 bp paired-end runs on an Illumina HiSeq4000 platform. The STAR aligner (version 2.4.2a)^36^ with modified parameter settings (–twopassMode = Basic) was used for split-read alignment against the mouse genome assembly mm39 (GRCm39) and UCSC known gene annotation. To quantify the number of reads mapping to annotated genes we used HTseq-count (version 0.6.0).^37^ FPKM (fragments per kilobase of transcript per million fragments mapped) values were calculated using custom scripts. Differential expression analysis was performed using the R Bioconductor package DESeq2 (version 1.42.1).^38^ The level of significance was set at a *P* value <0.05 and an absolute fold change |FC| >1.50. Gene Ontology pathway enrichment analyses were performed using the enrichGO functions of the “clusterProfiler” R package.^39^ A *P* value <0.005 was considered statistically significant. To visualize the enrichment analysis results, the “ggplot2” R package was used.^37^

### Mouse proteomics analysis

For proteomic analysis, left ventricular tissue was obtained from n = 11 WT, n = 11 α-MHC^719/+^, n = 4 α-MHC^719/+^ + pio, and n = 6 α-MHC^719/+^ + R-pio mice, with each sample derived from an individual heart.

Left ventricular tissue was cryo-pulverized (Pulverizer CP02, Covaris) and then lysed using sonication (Sonopuls HD3200, Bandelin) in 8 mol/L Urea/ 0.5 mol/L ammonium bicarbonate. Protein was digested with Lys-C (1:100; enzyme:protein) for 4 hours at 37 °C, then after dilution to 1 mol/L Urea/50 mmol/L ammonium bicarbonate and after addition of trypsin (1:50; enzyme:protein), digested overnight at 37 °C. Samples were analyzed on a Ultimate 3000 RSLC chromatograph coupled to a Q Exactive HF-X mass spectrometer (both Thermo Fisher Scientific). For the pio comparisons, 1.5 μg peptides were loaded onto a trap column (PepMap C18; 2 cm; 100 μm; Thermo Fisher Scientific) and separated (PepMap C18 analytical column; 75 μm; 50 cm; Thermo Fisher Scientific) using a 2-step gradient: first, a ramp from 3% B to 25% B for 160 minutes, followed by a 10-minute ramp to 40% B (A: 0.1% formic acid in water; B: 0.1% formic acid in acetonitrile). The mass spectrometer was run in data dependent mode and per survey scan a maximum of 15 product spectra were acquired. Raw data were then searched with MaxQuant (1.6.7.0)^40^ against all Swiss-Prot mouse entries and the built-in contaminant database. Match between runs and label-free quantification were turned on. For the R-pio samples, 1 μg of peptides were analyzed using the same instruments; however, the gradient comprised an initial ramp from 5% B to 20% B in 80 minutes and a subsequent ramp to 40% B in 9 minutes. The mass spectrometer was run in the data independent acquisition mode, product spectra were acquired utilizing 50 12-m/z wide isolation windows between 400 and 1,000 m/z. Raw data was searched using DIA-NN 1.8.1^41^ and all Swiss-Prot mouse entries. The mass spectrometry proteomics data have been deposited to the ProteomeXchange Consortium^42^ via the PRIDE partner repository^43^ with the data set identifier PXD067090 for the pio data and PXD067096 for the R-pio data. All statistical analyses and data visualization were performed using R statistical software version 4.3.1 (R Core Team, 2023). Proteins having at least 2 peptides detected in at least 2 replicates of each condition were tested for differential abundance using the MS-EmpiRe algorithm.^44^ Multiple testing correction was performed using the Benjamini–Hochberg method, and a false discovery rate <0.05 was considered significant. The STRING enrichment analysis (PMID: 36370105) was used to reveal biological processes associated with differentially abundant proteins (false discovery rate <0.05).

### Nucleic Acid Linked Immuno-Sandwich Assay protein quantification

Nucleic Acid Linked Immuno-Sandwich Assay (NULISA) protein quantification was performed using 25 μL of plasma from each sample (n = 9 WT, n = 10 α-MHC^719/+^, n = 4 α-MHC^719/+^+ R-pio, n = 5 WT+ R-pio). The NULISAseq library pool was generated using the Argo HT and the Mouse Panel 120 (version 2) (#801320, Lot 2502305, Alamar Biosciences). The final library pool was quantified using the 1× dsDNA high sensitivity Qubit kit (#Q33231, Invitrogen). The library pool was diluted in Illumina RSB (resuspension buffer) to a final concentration of 400 pmol/L for sequencing. 20 μL of the 400 pmol/L pool were loaded onto an XLEAP-SBS P2,100 cartridge (part number 20100987, Illumina: NextSeq 1000/2000 P2 XLEAP-SBS Reagent Kit [100 cycles]). The following settings were used to sequence the final pool and generate the fastq files: read 1: 34 nt; read 2: 0 nt; index 1: 0 nt; index 2: 0 nt; custom recipe: “Alamar_NSQ2K_R1Skip7_P2v4(4).xml.”

Protein quantification was performed with the ARGO Command Center (Alamar Biosciences) using the raw FASTQ files. The reads were demultiplexed, the counts were normalized for intraplate and interplate variation, and a log_2_ transformation was applied. The final read out was expressed in NULISA Protein Quantification units.

### NULISA analysis

Analysis of the NULISA data was performed using R statistical software. NULISA Protein Quantification expression values, along with sample annotation and target protein annotation, were imported and stored in an EList object from the limma package (version 3.60.0).^45^

Four samples were excluded based on quality control measures, as they had been flagged with warnings for both low detectability (<90% of targets above the limit of detection) and a low number of reads (<500,000). Other samples were excluded based on phenotypic criteria. The protein target *Hgf* was also excluded, because its expression was not above the limit of detection in at least 5 samples.

Empirical sample quality weights were calculated using the arrayWeights function. A design matrix was constructed using the sample group, and a linear model was fit using the lmFit function, including the sample quality weights. Comparisons were made between groups using the contrasts.fit function. Empirical Bayes moderated Student’s *t*-tests were performed and *P* values calculated using the eBayes function. Differentially expressed protein targets had a Benjamini-Hochberg adjusted *P* value <0.05.

### Statistics

Continuous data are expressed as the mean ± SD or median with 25th-75th percentiles (Q1-Q3). Normality was assessed using the Shapiro-Wilks test, whereas Levene's test was used to assess the equality of variances. Groups were compared using Student's *t*-test or the Mann-Whitney *U* test based on data distribution. IBM SPSS version 29.0.1 (IBM Corp, 2022) was used for statistical analyses, and a *P* value <0.05 was considered statistically significant.

## Results

### Pio and its R-enantiomer attenuate cardiac hypertrophy in vivo

**Figure 1:**
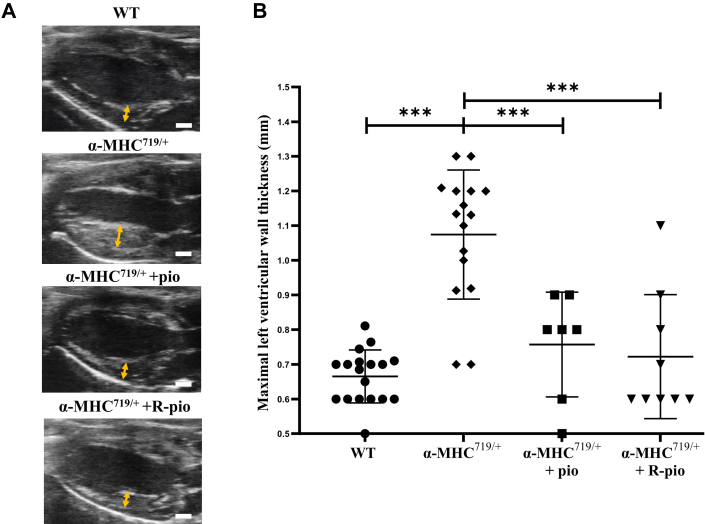
Pio and R-pio Reduce Hypertrophy in α-MHC^719/+^ (A) Representative transthoracic echocardiographic images in parasternal long-axis view showing left ventricular wall morphology across treatment groups. Orange arrows indicate the maximum left ventricular wall thickness. Scale bar = 1 mm. (B) Quantification of maximal left ventricular wall thickness in end-diastole (mean ± SD). Untreated α-MHC^719/+^ mice showed significant hypertrophy compared with untreated WT controls 1.07 ± 0.19 mm vs 0.67 ± 0.08 mm; *P* < 0.001). Treatment with pio reduced wall thickness to 0.76 ± 0.15 mm (29% reduction vs α-MHC^719/+^; *P* < 0.001), while R-pio further reduced it to 0.72 ± 0.18 mm (33% reduction vs α-MHC^719/+^; *P* < 0.001), reaching near-WT levels. Statistical comparisons between treatment groups and the untreated α-MHC^719/^^+^ control were performed using Dunnett's test for multiple comparisons following 1-way analysis of variance. ∗*P <* 0.05, ∗∗*P <* 0.01, ∗∗∗*P <* 0.001. Data information: WT: untreated WT (n = 18); α-MHC^719/+^: untreated α-MHC^719/+^ (n = 16), α-MHC^719/+^ +pio: α-MHC^719/+^ treated with pioglitazone (n = 7); α-MHC^719/+^ +R-pio: α-MHC^719/+^ treated with R-pioglitazone (n = 9).

Transthoracic echocardiography revealed marked left ventricular hypertrophy in untreated α-MHC^719/+^ mice compared with WT controls, as indicated by increased maximal end-diastolic wall thickness (1.07 ± 0.19 mm vs 0.67 ± 0.08 mm; *P* < 0.001), as shown in Figures 1A and 1B. Additional echocardiographic measures, including left ventricular end-diastolic diameter, left ventricular end-systolic diameter, ejection fraction, and fractional shortening, are summarized in Supplemental Table 1.

In contrast, maximal end-diastolic wall thickness in α-MHC^719/+^ mice treated with pio and R-pio was 0.76 ± 0.15 mm and 0.72 ± 0.18 mm, representing ∼29% and 33% reductions compared with untreated α-MHC^719/+^ mice (either *P <* 0.001), and approaching values observed in WT mice. Importantly, to rule out potential off-target effects, we also treated WT mice with pio or R-pio. Neither treatment induced myocardial hypertrophy in healthy hearts (Supplemental Figure 1A).

### Attenuation of Myocardial Fibrosis in α-MHC^719/+^ Mice by pio and R-pio

**Figure 2:**
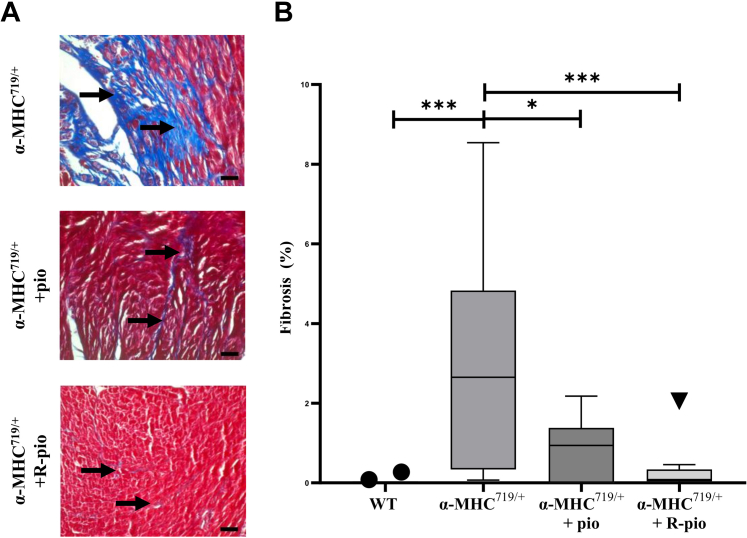
Attenuation of Fibrosis by pio and R-pio in α-MHC^719/+^ Mice (A) Representative Masson’s trichrome-stained transverse sections of the left ventricle. Viable myocardium appears red and collagen-rich fibrotic tissue blue (indicated by arrows). Scale bar = 50 μm. (B) Quantification of fibrotic area (percentage of total left ventricular area) for each group (median and 25th-75th percentiles). Untreated α-MHC^719/+^ mice exhibited a significant increase in fibrosis compared with WT controls (median fibrosis: 2.65% [Q1-Q3: 0.34%-4.82%] vs 0.00% [Q1-Q3: 0.00%-0.01%]; *P* < 0.001). Preventive treatment with pio reduced fibrosis to 0.94% [Q1-Q3: 0.00%-1.38%] (65% reduction vs α-MHC^719/+^) and with R-pio to near-baseline levels 0.08% [Q1-Q3: 0.00%-0.03%]; (>95% reduction vs α-MHC^719^). Statistical comparisons between groups were performed using the Mann-Whitney *U* test. ∗*P <* 0.05, ∗∗*P <* 0.01, ∗∗∗*P <* 0.001. Data information: WT: untreated WT (n = 21); α-MHC^719/+^: untreated α-MHC^719/+^ (n = 21); α-MHC^719/+^+pio: α-MHC^719/+^ treated with pioglitazone (n = 7); α-MHC^719/+^+R-pio: α-MHC^719/+^ treated with R-pioglitazone (n = 9).

Myocardial interstitial fibrosis was assessed using Masson’s Trichrome staining, with viable myocardium appearing red and collagen-rich fibrotic tissue blue (Figure 2A, scale bar: 50 μm).

Quantitative analysis of left ventricular sections (Figure 2B) showed a significant increase in fibrotic area in untreated α-MHC^719/+^ mice compared with WT controls (median fibrosis: 2.65% [Q1-Q3: 0.34%-4.82%] vs. 0.00% [Q1-Q3: 0.00%-0.01%]; *P* < 0.001). In α-MHC^719/+^ mice treated with pio or R-pio, median fibrotic area was 0.94% (Q1-Q3: 0.00%-1.38%) and 0.08% (Q1-Q3: 0.00%-0.03%), reflecting ∼65% and >95% reductions compared with untreated α-MHC^719/+^ mice *(P =* 0.036 and *P <*0.001, respectively). Similarly, Masson’s trichrome staining revealed no induction of interstitial fibrosis in WT mice treated with either pio or R-pio (Supplemental Figure 1B).

### RNA sequencing identifies therapeutic modulation of metabolic, mitochondrial, and inflammatory pathways in the left ventricle of α-MHC^719/+^ mice

To investigate the molecular mechanisms underlying the observed structural improvements, we performed RNA sequencing on left ventricular tissue. A total of 721 differentially expressed transcripts were identified (*P* value < 0.05, |FC| >1.50) comparing untreated α-MHC^719/+^vs untreated. WT mice, as shown in Supplemental Table 2. Pathway enrichment analysis revealed a profound transcriptional reprogramming characterized by the suppression of metabolic function and the simultaneous activation of remodeling and inflammatory pathways.

#### Suppression of mitochondrial and metabolic signaling

**Figure 3:**
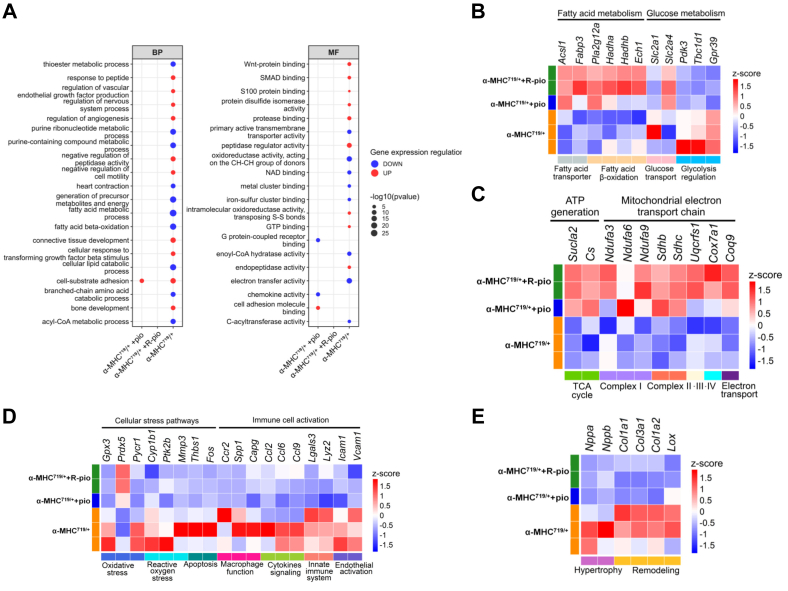
Transcriptional Profiling Reveals Metabolic Defects and Inflammatory Signaling in α-MHC^719/^^+^ Mice, Largely Ameliorated by Treatment With Pio and R-Pio (A) Dot plots of the top enriched Gene Ontology terms for Biological Processes (BP) and Molecular Functions (MF). Untreated α-MHC^719/^^+^ myocardium exhibits a marked suppression of fatty acid metabolic processes (blue circles) and up-regulation of structural remodeling pathways (red circles). Treatment with pio or R-pio largely normalizes these signatures. Circle size reflects enrichment significance (–log_10_ p). (B to E) Heatmaps illustrating normalized expression (Z-scores) of representative differentially expressed genes compared with untreated WT myocardium. (B) Fatty acid and glucose metabolism: Genes governing fatty acid β-oxidation are down-regulated in untreated α-MHC^719/^^+^ hearts and restored by treatment, while glucose metabolism genes show a compensatory up-regulation that is modulated by therapy. (C) Mitochondrial bioenergetics: Key components of the TCA cycle and mitochondrial electron transport chain (Complex I–IV) are suppressed in the disease group and upregulated following treatment. (D) Stress and immune activation: Markers of oxidative stress and immune cell recruitment are elevated in disease. Notably, *Prdx5*, a mitochondrial antioxidant, is downregulated in untreated hearts and restored with therapy. (E) Hypertrophy and fibrosis: The fetal gene program (*Nppa*, *Nppb*) and extracellular matrix components (*Col1a1*, *Col3a1*) are strongly induced in untreated α-MHC^719/^^+^ mice and effectively suppressed by both pio and R-pio. Data information: WT: untreated WT; α-MHC^719/^^+^: untreated α-MHC^719/^^+^; α-MHC^719/^^+^+ pio: α-MHC^719/+^ treated with pioglitazone; α-MHC^719/^^+^+ R-pio: α-MHC^719/+^ treated with R-pioglitazone.

The most prominent molecular feature of untreated α-MHC^719/+^ hearts was a broad down-regulation of pathways essential for cardiac energy homeostasis (Figures 3A to 3C, Supplemental Table 3).

Lipid and glucose metabolism: Genes governing fatty acid uptake (eg, *Fabp3*, *Acsl1*) and β-oxidation (*Hadha*, *Hadhb*, *Ech1*) were consistently suppressed. This was accompanied by a pathological "metabolic switch" in glucose handling, characterized by the up-regulation of the insulin-independent transporter *Slc2a1* (*GLUT1*) and down-regulation of the insulin-dependent *Slc2a4* (*GLUT4*) (Figures 3A and 3B).

Mitochondrial energy production: The metabolic deficit extended to the mitochondrial respiration machinery. Components of the TCA cycle (eg, *Sucla2*, *Cs*) and subunits across the electron transport chain—including Complex I (*Ndufa3*, *Ndufa9*), Complex II (*Sdhb*), and Complexes III/IV (*Uqcrfs1*, *Cox7a1*)—were significantly reduced (Figure 3C). Analysis of molecular functions confirmed this deficit, showing down-regulation of electron transfer and oxidoreductase activities (Figures 3A and 3C).

Treatment with pio or R-pio largely prevented this metabolic depression. As shown in the heatmaps in Figures 3B and 3C, expression levels of key fatty acid oxidation enzymes and mitochondrial respiratory complex subunits were maintained at near-WT levels in both treatment groups.

#### Activation of structural remodeling, hypertrophic stress, and stress pathways

In contrast to the metabolic down-regulation, pathways driving pathological remodeling and cellular stress were markedly up-regulated in untreated α-MHC^719/^^+^ hearts (Figures 3A, 3D, and 3E, Supplemental Table 4).

Structural remodeling: We observed significant enrichment of processes related to extracellular matrix organization (connective tissue development), angiogenesis (regulation of VEGF production), and cell–substrate adhesion (Figure 3A).

Profibrotic and inflammatory signaling: Maladaptive remodeling was associated with the activation of classical pro-hypertrophic signal transduction pathways, including Wnt, MAPK, and SMAD protein binding (Figure 3A). Furthermore, the hearts exhibited a distinct stress signature (Figure 3D), characterized by the up-regulation of oxidative stress markers (*Gpx3*), apoptosis-related genes (*Lgals3*, *Thbs1*), and proinflammatory chemokines (*Ccl2*, *Ccl6*, *Spp1*), alongside a reduction in the antioxidant *Prdx5*.

Both pio and R-pio effectively blunted these maladaptive pathways. The activation of Wnt, MAPK, and SMAD signaling was attenuated, and the expression of hypertrophic markers (*Nppa*, *Myh7*) as well as oxidative stress and inflammatory mediators was normalized to levels comparable to WT controls (Figures 3A, 3E, Supplemental Table 4).

### Proteomic evidence supports transcriptomic findings and indicates therapeutic recovery

To validate the transcriptomic findings at the functional protein level, we performed quantitative proteomics on left ventricular tissue. We identified 487 proteins significantly altered in untreated α-MHC^719/^^+^ hearts compared with WT (Supplemental Table 7). Consistent with the RNA-seq data, the proteomic landscape was characterized by a dichotomy of suppressed bioenergetics vs activated remodeling pathways.

#### Restoration of mitochondrial and metabolic protein networks

**Figure 4:**
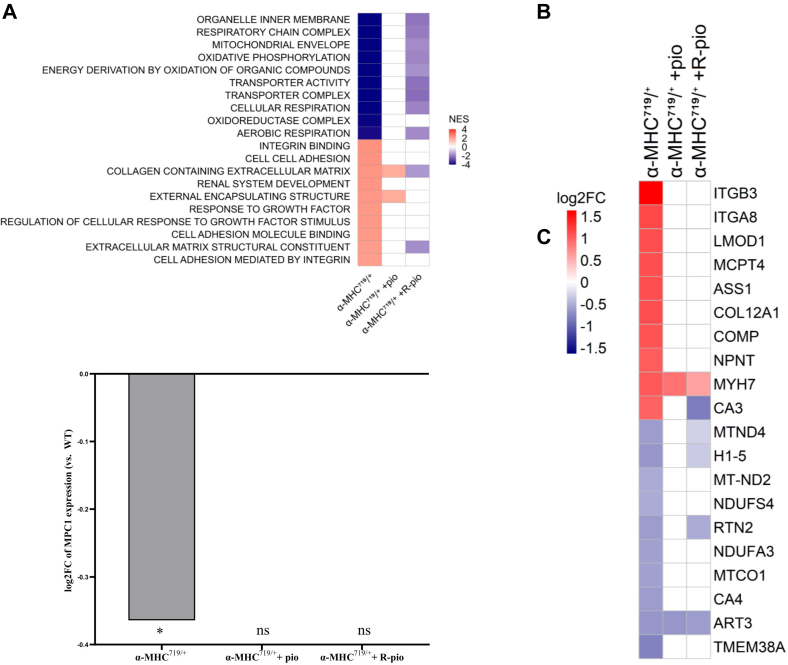
Impact of Treatment With Pio and R-pio on the Proteomic Profiling in α-MHC^719/^^+^ Hearts (A) Gene Ontology enrichment of untreated α-MHC^719/^^+^ vs WT shows suppressed mitochondrial metabolism and activated inflammatory/proliferative pathways, largely normalized by treatment. Normalized enrichment scores (NES) indicate pathway direction and strength.(B) Heatmap of the top 10 up- and down-regulated proteins in untreated α-MHC^719/^^+^ vs WT. Protein abundance relative to the untreated WT controls is expressed as log_2_ fold change (log_2_ FC). Untreated α-MHC^719/^^+^ vs WT controls show increased extracellular matrix/hypertrophy proteins and decreased mitochondrial/redox proteins, largely normalized by treatment. (C) Log_2_ FC of *MPC1* is significantly reduced in α-MHC^719/+^ vs WT (adj. *P <*0.03), but restored to WT levels by both treatments (ns). *P* values were adjusted for multiple testing using the Benjamini-Hochberg method. ∗*P <* 0.05, ∗∗*P <* 0.01, ∗∗∗*P <* 0.001, ns = not significant. Data information: WT: untreated WT, α-MHC^719/^^+^: untreated α-MHC^719/^^+^; α-MHC^719/^^+^+ pio: α-MHC^719/+^ treated with pioglitazone; α-MHC^719/^^+^+ R-pio: α-MHC^719/+^ treated with R-pioglitazone.

The most profound proteomic change was the depletion of proteins essential for mitochondrial respiration (Figure 4A). We observed a broad down-regulation of subunits across the electron transport chain (eg, *NDUFA3*, *MT-CO1*) and ATP synthase (*ATP5PF*) (Figure 4B). Mechanistically relevant to the drugs' mode of action, *MPC1* was significantly reduced in the disease model (Figure 4E, Supplemental Table 5). Importantly, treatment with pio or R-pio restored the abundance of these mitochondrial proteins and *MPC1* toward WT levels (Figure 4E, Supplemental Tables 6 and 7) Abundance levels of *MPC2* and *ACSL4* remained unchanged across all α-MHC^719/+^ groups compared with WT (Supplemental Tables 5 to 7).

#### Validation of inflammatory signaling: Macrophage infiltration and systemic inflammatory tone

To validate the inflammatory signature identified in our transcriptomic and proteomic data at the cellular level, we performed immunohistochemical staining for macrophage markers on left ventricular tissue sections.

**Figure 5:**
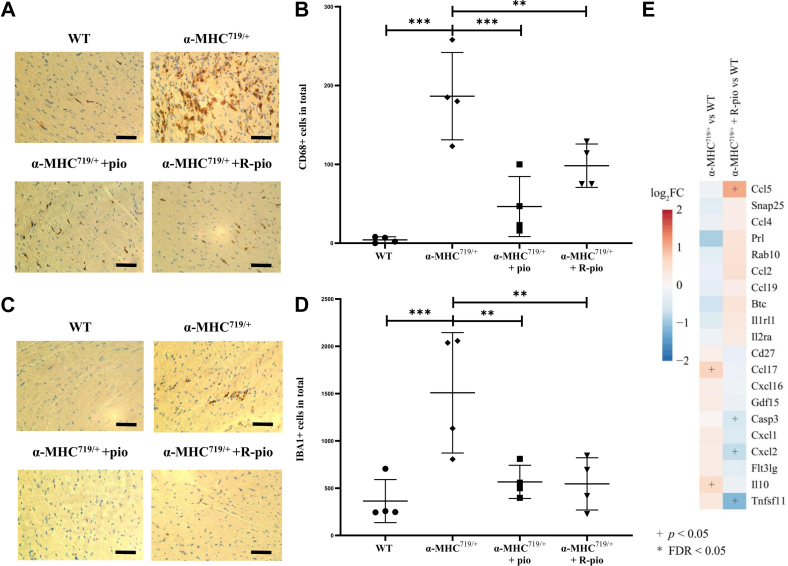
Attenuation of Myocardial Macrophage Infiltration and Systemic Inflammatory Signaling (A to D) Immunohistochemical validation of macrophage infiltration in left ventricular tissue. (A and B) Representative staining (A) and quantification (B) of IBA1-positive macrophages (mean ± SD). Scale bar = 50 μm. Untreated α-MHC^719/^^+^ hearts show a significant accumulation of IBA1^+^ cells compared with WT. Treatment with both pio and R-pio significantly reverses this infiltration. (C and D) Representative staining (C) and quantification (D) of CD68-positive macrophages (mean ± SD). Pan-macrophage numbers are elevated in disease. Pio and R-pio treatment significantly reduces CD68^+^ cell counts. (E) Systemic inflammatory profiling (NULISA) of plasma samples. The heatmap displays the top 10 up- and down-regulated proteins in untreated α-MHC^719/^^+^ vs WT. Scatter plots represent individual biological replicates (n = 4 per group); horizontal lines indicate mean ± SD. Statistical comparisons between treatment groups and the untreated α-MHC^719/^^+^ control were performed using Dunnett's test for multiple comparisons following 1-way analysis of variance. ∗*P <* 0.05, ∗∗*P <* 0.01, ∗∗∗*P <* 0.001. Data information: WT: untreated WT; α-MHC^719/^^+^: untreated α-MHC^719/^^+^; α-MHC^719/^^+^+ pio: α-MHC^719/+^ treated with pioglitazone. α-MHC^719/^^+^+ R-pio: α-MHC^719/+^ treated with R-pioglitazone.

Myocardial macrophage infiltration: As shown in Figures 5A and 5C, untreated α-MHC^719/^^+^ hearts exhibited a dense infiltration of immune cells compared with sparse resident cells in WT controls. Quantitative analysis confirmed a significant accumulation of macrophages:

• *IBA1⁺ cells:* The number of IBA1-positive macrophages was sharply increased in untreated α-MHC^719/^^+^ mice compared with WT (*P <* 0.001). Treatment with both pio *(P =* 0.002) and R-pio *(P =* 0.001) significantly reversed this accumulation, reducing cell numbers toward WT levels (Figure 5B).
• *CD68⁺ cells:* Similarly, pan-macrophage staining (CD68) was significantly elevated in the disease model (*P <* 0.001 vs WT). Pio and R-pio treatment significantly reduced CD68^+^ cell counts (*P <* 0.001 and *P =* 0.001, respectively) (Figure 5D).

Finally, treatment of WT controls with pio or R-pio did not trigger macrophage infiltration or immune cell activation (Supplemental Figures 1C and 1D).

Systemic inflammatory profiling (NULISA): To determine whether this marked local inflammation translated into a broad systemic response, we analyzed plasma samples using the ultra-sensitive NULISA proteomic platform. In contrast to the pronounced myocardial infiltration, the systemic inflammatory footprint was more restricted (Figure 5E). Although many classical proinflammatory markers (eg, *Cxcl1*, *Tnfs11*) showed variable up-regulation patterns without reaching statistical significance, we identified a specific elevation of the chemokine *Ccl17* and the immunomodulatory cytokine *Il10* (*P <* 0.05 compared with WT). Treatment with R-pio appeared to modulate this specific systemic signature. As shown in the right column of Figure 5E, the levels of *Ccl17* and *Il10* in R-pio–treated mice were no longer significantly elevated compared with WT controls.

## Discussion

### Targeting the metabolic-inflammatory axis reverses HCM remodeling

In this study, we demonstrate that pharmacological targeting of mitochondrial and inflammatory pathways using pio or its PPARγ-inactive enantiomer effectively reverses pathological remodeling in a genetic mouse model of HCM. Treatment significantly blunted the hallmarks of the disease: it reduced left ventricular hypertrophy by up to 33% and interstitial fibrosis by >95%, restoring myocardial architecture toward a WT phenotype. These structural improvements were underpinned by a profound molecular recovery. Using a multi-omics approach validated by histology, we show that pio and R-pio normalize the “pathogenic triad” of HCM: 1) they restore suppressed mitochondrial bioenergetics; 2) they silence the transcriptional reactivation of fetal stress markers (*Nppa*, *Myh7*); and 3) they resolve the pronounced local macrophage infiltration. Crucially, R-pio achieved these benefits without the PPARγ-mediated side effects typically limiting thiazolidinedione use, highlighting its potential as a disease-modifying therapy.

### Restoring bioenergetics to counteract mechano-energetic uncoupling

A central finding of our study is the restoration of mitochondrial respiration and fatty acid oxidation pathways by pio and R-pio. Sarcomeric alterations in HCM increase the energetic cost of contraction, leading to a state of “mechano-energetic uncoupling” where ATP demand outstrips supply.^10^ This energy deficit forces the heart to rely on inefficient glucose metabolism (metabolic switch) and impairs mitochondrial respiration, as evidenced by the down-regulation of Complex I/IV subunits and TCA cycle enzymes in our untreated mice. Consistent with the model proposed by Kohlhaas et al^10^ and recent metabolic profiling by Nollet et al,^46^ this mitochondrial dysfunction likely drives the production of ROS, creating a pro-oxidant environment (up-regulation of *Gpx3*, down-regulation of *Prdx5*) that triggers secondary damage. Our proteomic data suggest that pio and R-pio intervene centrally in this cascade. Mechanistically, both agents are known modulators of the MPC. We observed a significant restoration of *MPC1* protein levels in treated hearts. By stabilizing mitochondrial substrate flux and preventing the maladaptive reliance on glycolysis, these agents likely reduce oxidative stress and improve the bioenergetic reserve, breaking the vicious cycle of energy depletion.

### Inflammation in HCM: A local driver mirrored by restricted systemic signals

Although inflammation has long been debated as either a cause or consequence in HCM, our data provide direct evidence of immune cell involvement. We identified a robust infiltration of IBA1+ and CD68+ macrophages in the interstitium of diseased hearts, which was significantly cleared by treatment. Interestingly, our systemic profiling (NULISA) revealed a restricted inflammatory footprint in plasma compared with the massive local infiltration. Although specific markers like *Ccl17* and *Il10* were elevated and normalized by R-pio, the absence of a broad, unspecific cytokine storm suggests that inflammation in this model is primarily a tissue-autonomous response to cardiomyocyte stress and cell death, rather than a systemic autoimmune phenomenon. This supports a model where metabolic stress and ROS in cardiomyocytes stimulate the release of chemokines (eg, *Ccl2*, *Spp1*, up-regulated in our RNA-seq), recruiting macrophages that drive interstitial fibrosis.^47^ By resolving the upstream metabolic defect, pio and R-pio shut down this chemotactic signal, leading to the observed resolution of both inflammation and fibrosis.

### **R-**pio**: Efficacy independent of PPARγ**

Clinically, the use of classical thiazolidinediones is hampered by PPARγ-driven adverse events. A key finding of our work is that R-pio was equipotent (and in fibrosis reduction even superior) to the racemic mixture. Because R-pio lacks significant genomic PPARγ activity,^29^ its efficacy supports the hypothesis that the cardioprotective effects are mediated through noncanonical pathways, likely involving direct mitochondrial stabilization (eg, via *MPC* or *ACSL4* modulation). Furthermore, the fact that treatment did not induce structural or functional alterations in healthy WT mice underscores a favorable safety profile and suggests a disease-specific mechanism of action. This positions R-pio as a promising candidate for clinical translation, decoupling therapeutic efficacy from metabolic toxicity.

### Study limitations

Although this study demonstrates robust structural and molecular improvements with both pio and its R-enantiomer, several limitations should be acknowledged.

First, a limitation of the transcriptomic analysis in the pio cohort was the use of pooled RNA samples. Although pooling is a common strategy in pilot transcriptomic studies to obtain sufficient material and reduce individual noise, it inevitably leads to a loss of information regarding biological variability within the group. Consequently, subtle individual differences in gene expression might be masked. However, to mitigate this, we validated the key transcriptomic findings (e.g., mitochondrial and inflammatory signatures) using individual heart samples in the subsequent R-pio cohort and at the protein level via quantitative proteomics, which confirmed the robustness of our results.

Second, to accelerate disease onset, we employed cyclosporine A as a disease accelerator. Although cyclosporine A does not induce cardiomyopathy in WT animals, its immunomodulatory properties could theoretically confound the assessment of inflammatory markers. However, the fact that we observed a significant macrophage infiltration and inflammatory signature despite the background presence of cyclosporine A suggests that the proinflammatory drive in this HCM model is highly potent and that the observed therapeutic rescue extends beyond mere immunomodulation.

Third, although R-pio is considered PPARγ-inactive, partial in vivo racemization to the S-enantiomer, which possesses classical PPARγ agonist activity, cannot be entirely excluded. However, the distinct proteomic profile of R-pio observed in our study—characterized by a superior reduction of fibrosis and stronger induction of mitochondrial biogenesis compared with racemic pio—argues for a specific mechanism of action that is at least partially independent of canonical PPARγ signaling.

Fourth, our multi-omics profiling revealed an unexpected upregulation of the chemokine CCL5 specifically in response to R-pio. This is notable because classical PPARγ activation typically suppresses proinflammatory chemokines, including CCL5, through transrepression of NF-κB signaling.^48^ The induction of CCL5 suggests the activation of alternative, noncanonical pathways, and its long-term impact on cardiac immune homeostasis warrants further investigation. Fifth, the precise molecular targets and downstream signaling pathways engaged by R-pio remain incompletely defined. Although the compound appears to normalize metabolic, mitochondrial, and inflammatory signatures (including *MPC1* levels), the exact receptor interactions or nongenomic effects responsible for these actions require further mechanistic validation.

Sixth, our findings are based on a single murine model of sarcomeric HCM. Although the α-MHC^719/^^+^ mouse faithfully recapitulates key human disease features, species-specific differences in metabolic regulation and physiology exist. This is particularly relevant when considering previous clinical reports of pio-exacerbated heart failure, which are primarily attributed to PPARγ-mediated renal sodium reabsorption and fluid retention.^49^ Although R-pio is specifically designed to circumvent these PPARγ-driven side effects and we observed no signs of fluid overload in our mice, the risk of heart failure exacerbation remains a critical consideration for clinical translation. Therefore, further validation in human models, such as patient-derived induced pluripotent stem cell cardiomyocytes or human myocardial tissue, will be essential to confirm the translational relevance and safety profile of R-pio in a human genetic background before clinical application.

## Conclusions

We identify a profound metabolic and inflammatory defect in HCM that drives structural progression. Pio and R-pio effectively interrupt this pathogenic spiral by restoring mitochondrial integrity and resolving local inflammation. These findings support a paradigm shift from purely hemodynamic management toward metabolic therapies in HCM.

Perspectives **COMPETENCY IN MEDICAL KNOWLEDGE:** This study reveals that dysregulation of key biological pathways, including myocardial substrate metabolism, mitochondrial energy generation, and inflammatory signaling, constitutes a core pathogenic mechanism in HCM. Pharmacological modulation of these pathways not only corrected the underlying molecular alterations but also led to significant reductions in myocardial fibrosis and hypertrophy, supporting a direct mechanistic link between metabolic dysregulation and phenotypic disease expression.

**TRANSLATIONAL OUTLOOK:** Current pharmacologic therapies for HCM primarily target symptoms or outflow tract obstruction and offer limited options for patients with nonobstructive or early-stage disease. This study provides preclinical evidence that metabolic reprogramming can attenuate key pathological features of HCM upstream of structural remodeling. Clinical trials are warranted to evaluate whether R-pio or similar compounds can serve as safe and effective disease-modifying treatments in patients with HCM.

## Funding Support and Author Disclosures

This work was supported by the Else-Kröner Fresenius Stiftung and Förderverein des Deutschen Herzzentrums (to Dr Wolf), Stiftung Kinderherz (to Dr Wolf), European Research Council—ERCAd Grant 788381 (to Dr Moretti), and German Research Foundation–Transregio Research Units 152 and 267 (to Dr Moretti). Dr Moretti is funded by the European Research Council (ERCAd grant 788381); and is a principal investigator of the Transregio Research Units 152 and 267, funded by the German Research Foundation. Dr Wolf has received honoraria from Novo Nordisk and Bristol-Myers Squibb; has served as a consultant for Day One Biopharmaceuticals, Inc, BioMarin Pharmaceuticals, Adrenomed AG, Pliant Therapeutics, Anacardio, and Rocket Pharmaceuticals; and has ownership interest in Preventage Therapeutics. All other authors have reported that they have no relationships relevant to the contents of this paper to disclose.
