# Supplementary material for: Targeting Inflammation by Pioglitazone and its R-Enantiomer Mitigates Pathological Myocardial Remodeling in Murine Hypertrophic Cardiomyopathy
Source: JACC Basic Transl Sci. 2026 Jun 13;11(7):101574. doi: 10.1016/j.jacbts.2026.101574 (PMC13279018; doi:10.1016/j.jacbts.2026.101574)
Supplement: Supplemental Figure 1 and Supplemental Tables 1-7 [file mmc1.docx]

Contents

[1 Supplemental Figure S1. Safety profile of Pioglitazone and R-Pioglitazone in wild-type mice. 2](#_Toc227576688)

[2 Supplemental Table 1- Echocardiographic parameters. 3](#_Toc227576689)

[3 Supplemental Table 2- Differentially expressed transcripts (p < 0.05 |fold change (FC)| > 1.5) between untreated α-MHC^719/+^ and wildtype mice 5](#_Toc227576691)

[4 Supplemental Table 3- Downregulated pathways (p < 0.05) based on transcriptomic data comparing untreated α-MHC^719/+^ and wildtype mice 22](#_Toc227576692)

[P-values were adjusted using the Benjamini–Hochberg method; q-values indicate the false discovery rate (FDR). 53](#_Toc227576693)

[5 Supplemental Table 4- Upregulated pathways (p < 0.05.) based on transcriptomic data comparing untreated α-MHC^719/+^ and wildtype mice 54](#_Toc227576694)

[6 Supplemental Table 5- Differentially expressed protein (p.adj < 0.05) between untreated α-MHC^719/+^ and wildtype mice 118](#_Toc227576695)

[7 Supplemental Table 6- Differentially expressed protein (p.adj < 0.05)between α-MHC^719/+^ treated with pio and wildtype mice 129](#_Toc227576696)

[8 Supplemental Table 7- Differentially expressed protein (p.adj < 0.05)between α-MHC^719/+^ treated with R-pio and untreated wildtype mice 131](#_Toc227576697)

# Supplemental Figure S1. Safety profile of Pioglitazone and R-Pioglitazone in wild-type mice.


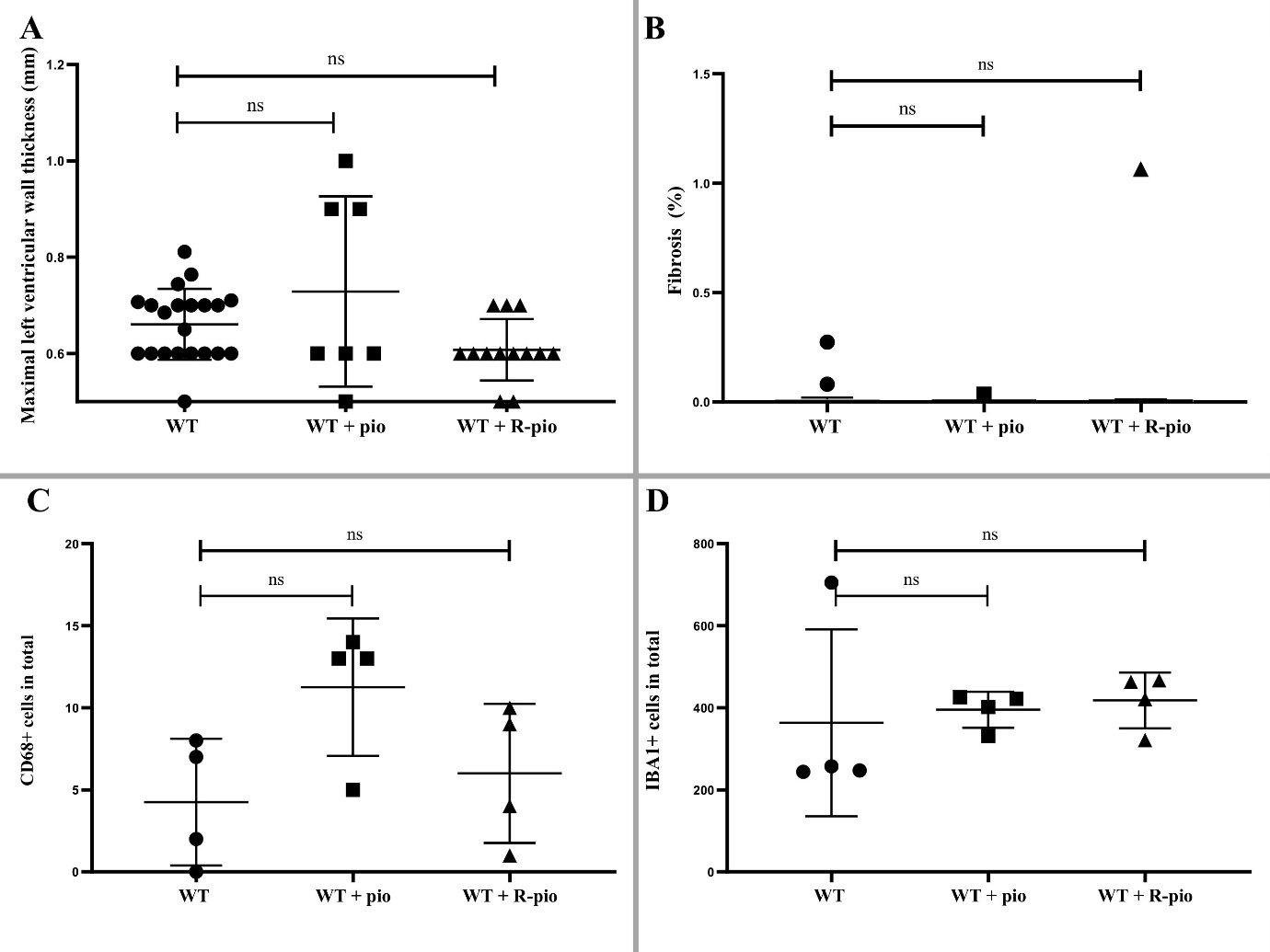


**(A)** Quantification of maximal left ventricular wall thickness in end-diastole (mean ± SD). **(B)** Quantification of myocardial fibrotic area as a percentage of total left ventricular area (median and 25th-75th percentiles). **(C, D)** Quantification of myocardial macrophage infiltration using IBA1 **(C)** and CD68 **(D**) positive cell counts (mean ± SD). As shown, treatment of healthy WT mice with either pio or R-pio did not induce myocardial hypertrophy, interstitial fibrosis, or trigger macrophage infiltration.

Data information: Normality of data distribution was assessed using the Shapiro-Wilk test. Statistical comparisons between the treated WT groups and the untreated WT control were performed using one-way ANOVA followed by Dunnett's test for normally distributed data **(A, C, D)** or the Mann-Whitney U test for non-parametrically distributed data **(B).** ns = not significant; *P < 0.05, **P < 0.01, ***P < 0.001.

WT: untreated wild type ; α-MHC^719/+^: Untreated α-MHC^719/+^; α-MHC^719/+^+pio: α-MHC^719/+^ treated with pioglitazone; α-MHC^719/+^+ R-Pio: α-MHC^719/+^ treated with R-pioglitazone.

# Supplemental Table 1- Echocardiographic parameters.

| **Parameter** | **Unit** | **WT** | **α-MHC^719/+^** | **α-MHC^719/+^ +Pio** | **α-MHC^719/+^ +R-Pio** |
| --- | --- | --- | --- | --- | --- |
| **n** | - | 21 | 22 | 7 | 9 |
| **LVEDD** | mm | 2.7 ± 0.1 | 2.4 ± 0.1, ns | 3.5 ± 0.1, ** | 2.9 ± 0.2, ns |
| **LVESD** | mm | 1.3 ± 0.2 | 1.1 ± 0.1, ns | 2.1 ± 0.2, ns | 1.6 ± 0.2, ns |
| **LVEDD/LVWT**_max_ | - | 4.1 ± 0.2 | 3.6 ± 0.2, ns | 5.2 ± 0.2, ** | 4.3 ± 0.3, ns |
| **EF** | % | 87.1 ± 2.2 | 90.2 ± 1.5, ns | 77.1 ± 3.8, ns | 82.5 ± 3.7, ns |
| **FS** | % | 53.7 ± 3.2 | 56.0 ± 2.3, ns | 41.0 ± 3.4, * | 47.0 ± 4.1, ns |

Data are expressed as mean ± SD. The normality of data distribution was confirmed using the Shapiro-Wilk test. Statistical comparisons were performed between the treated groups (α-MHC⁷¹⁹/⁺ + pio, α-MHC⁷¹⁹/⁺ + R-pio) and the untreated WT control group using one-way ANOVA followed by Dunnett's test for multiple comparisons . Asterisks indicate statistical significance compared to the untreated WT group: ns = not significant; *P < 0.05, **P < 0.01, *P < 0.001.

WT: untreated wild type ; α-MHC^719/+^: Untreated α-MHC^719/+^; α-MHC^719/+^+pio: α-MHC^719/+^ treated with pioglitazone; α-MHC^719/+^+ R-Pio: α-MHC^719/+^ treated with R-pioglitazone.

# Supplemental Table 2- Differentially expressed transcripts (p < 0.05 |fold change (FC)| > 1.5) between untreated α-MHC^719/+^ and wildtype mice

| **gene** | **baseMean** | **log2FC** | **Log2FC SE** | **stat** | **pvalue** |
| --- | --- | --- | --- | --- | --- |
| *Tmem150c* | 24.66578 | -3.10285 | 0.841611 | 14.00094 | 0.002904 |
| *Lgi1* | 73.17065 | -3.05231 | 0.527886 | 37.09992 | 4.38E-08 |
| *Cngb3* | 26.46497 | -2.21383 | 0.827061 | 9.254338 | 0.026094 |
| *Hcn4* | 53.06899 | -2.10655 | 0.568356 | 13.30085 | 0.004029 |
| *Irx1* | 83.96567 | -1.91375 | 0.591002 | 12.27719 | 0.006491 |
| *Iigp1* | 542.6813 | -1.83657 | 0.276126 | 42.86975 | 2.62E-09 |
| *Ky* | 150.4527 | -1.75042 | 0.370736 | 23.78095 | 2.78E-05 |
| *Xist* | 182.8257 | -1.74794 | 0.48343 | 36.18599 | 6.84E-08 |
| *Frmpd3* | 38.48597 | -1.74248 | 0.647155 | 8.777414 | 0.032401 |
| *Snai3* | 41.66182 | -1.74144 | 0.723607 | 8.109696 | 0.043798 |
| *D630045M09Rik* | 31.30197 | -1.71494 | 0.728014 | 8.052329 | 0.044943 |
| *Mylk4* | 785.1542 | -1.71064 | 0.288488 | 39.24472 | 1.54E-08 |
| *Olfr78* | 40.02298 | -1.62687 | 0.694956 | 10.51914 | 0.014632 |
| *Abca4* | 131.2645 | -1.61328 | 0.412195 | 16.89132 | 0.000744 |
| *Slc15a2* | 54.34945 | -1.59247 | 0.704126 | 8.236552 | 0.041368 |
| *Adra1a* | 109.6067 | -1.54416 | 0.488146 | 11.97479 | 0.00747 |
| *Drd2* | 65.09555 | -1.53224 | 0.530063 | 8.815328 | 0.03185 |
| *Cyfip2* | 233.6297 | -1.53128 | 0.315778 | 27.17125 | 5.42E-06 |
| *Ddit4* | 852.2076 | -1.51454 | 0.244928 | 38.45632 | 2.26E-08 |
| *AI507597* | 165.0757 | -1.48245 | 0.384804 | 15.52358 | 0.00142 |
| *3110057O12Rik* | 494.9875 | -1.47855 | 0.268322 | 32.08012 | 5.03E-07 |
| *Igfals* | 65.84994 | -1.47441 | 0.533746 | 8.918787 | 0.03039 |
| *Epn3* | 76.50694 | -1.46972 | 0.483707 | 14.10164 | 0.00277 |
| *Rsad2* | 231.1539 | -1.43498 | 0.406877 | 12.08751 | 0.007089 |
| *Cntn2* | 269.2224 | -1.4311 | 0.396192 | 12.82427 | 0.005032 |
| *Tmem82* | 326.3354 | -1.42275 | 0.330131 | 19.90312 | 0.000178 |
| *AK043886* | 46.231 | -1.40983 | 0.594157 | 7.994266 | 0.04613 |
| *Acot1* | 221.5492 | -1.35464 | 0.44479 | 15.58549 | 0.001379 |
| *Gbp10* | 265.8453 | -1.26346 | 0.337683 | 16.93143 | 0.00073 |
| *Pxmp2* | 186.365 | -1.25891 | 0.362616 | 15.01797 | 0.001801 |
| *8430408G22Rik* | 1276.604 | -1.23481 | 0.279338 | 20.39597 | 0.000141 |
| *Mme* | 77.17377 | -1.20209 | 0.48625 | 9.542304 | 0.022886 |
| *Lgals4* | 248.5601 | -1.18261 | 0.373498 | 12.1623 | 0.006847 |
| *Gbp6* | 553.0968 | -1.17858 | 0.283109 | 20.80253 | 0.000116 |
| *Cryba4* | 76.18783 | -1.16885 | 0.474117 | 10.55672 | 0.014381 |
| *Efnb3* | 1125.574 | -1.16434 | 0.266395 | 20.53487 | 0.000131 |
| *Cdh23* | 90.59831 | -1.1568 | 0.485086 | 8.771415 | 0.03249 |
| *Whrn* | 431.7781 | -1.14696 | 0.293085 | 20.90493 | 0.00011 |
| *Tcp11l2* | 1180.578 | -1.1422 | 0.215751 | 28.38335 | 3.02E-06 |
| *Ehhadh* | 127.206 | -1.1382 | 0.387942 | 8.593981 | 0.035206 |
| *Ptprn2* | 226.1627 | -1.1359 | 0.311121 | 14.24641 | 0.002588 |
| *Timp4* | 883.9224 | -1.13494 | 0.225753 | 35.88636 | 7.91E-08 |
| *Scn4a* | 178.8933 | -1.13141 | 0.426167 | 8.986643 | 0.029469 |
| *Gstm7* | 348.1785 | -1.12134 | 0.309354 | 15.7638 | 0.001268 |
| *Gpr22* | 210.8746 | -1.113 | 0.341365 | 19.50883 | 0.000215 |
| *Tcea3* | 368.7285 | -1.10412 | 0.34134 | 11.2288 | 0.010551 |
| *Ephb1* | 349.7115 | -1.10183 | 0.304559 | 14.73013 | 0.002062 |
| *Asb14* | 689.6651 | -1.10069 | 0.288317 | 18.03605 | 0.000432 |
| *Cpeb3* | 1057.998 | -1.09785 | 0.282937 | 20.81691 | 0.000115 |
| *Aqp7* | 326.8071 | -1.08846 | 0.277044 | 18.30117 | 0.000381 |
| *Fitm1* | 929.5487 | -1.07892 | 0.233469 | 29.61771 | 1.66E-06 |
| *mt-tRNA-Met* | 1185.437 | -1.06337 | 0.230627 | 24.22029 | 2.25E-05 |
| *Trim7* | 357.2395 | -1.06189 | 0.273888 | 23.09911 | 3.85E-05 |
| *Slc25a34* | 2601.387 | -1.05978 | 0.247098 | 21.17913 | 9.66E-05 |
| *Art5* | 165.2482 | -1.05327 | 0.390188 | 9.591534 | 0.022377 |
| *Abcc9* | 2419.974 | -1.05043 | 0.209483 | 29.53639 | 1.73E-06 |
| *mt-16SrRNA* | 144959.8 | -1.01301 | 0.211492 | 35.34892 | 1.03E-07 |
| *mt-Nd2* | 130204.9 | -1.01233 | 0.216871 | 24.16157 | 2.31E-05 |
| *Cdkn1c* | 287.7316 | -1.01093 | 0.286249 | 12.74601 | 0.005219 |
| *Asb15* | 647.2361 | -1.01 | 0.239825 | 21.72124 | 7.45E-05 |
| *1190002H23Rik* | 345.9291 | -1.00966 | 0.306234 | 12.6299 | 0.005509 |
| *Gpt* | 375.5058 | -1.00939 | 0.333197 | 13.19695 | 0.004229 |
| *Pdp2* | 105.2462 | -1.00885 | 0.420997 | 7.934032 | 0.047395 |
| *Sh3rf2* | 726.4125 | -1.00089 | 0.238249 | 25.41896 | 1.26E-05 |
| *Magix* | 299.9394 | -0.99968 | 0.30389 | 13.58668 | 0.003525 |
| *Dusp18* | 1058.198 | -0.99461 | 0.265334 | 15.55293 | 0.0014 |
| *Kcnv2* | 193.8069 | -0.98934 | 0.364591 | 13.00194 | 0.004632 |
| *Wfdc1* | 198.3739 | -0.98869 | 0.332783 | 10.8963 | 0.0123 |
| *Ano10* | 649.0126 | -0.98197 | 0.271728 | 18.70793 | 0.000314 |
| *9030617O03Rik* | 369.331 | -0.9754 | 0.347863 | 8.857897 | 0.031241 |
| *Myh7b* | 2089.803 | -0.97468 | 0.226456 | 32.07247 | 5.05E-07 |
| *Sorcs2* | 198.2568 | -0.97071 | 0.367053 | 9.458986 | 0.023772 |
| *Etl4* | 450.1658 | -0.97028 | 0.253751 | 18.1774 | 0.000404 |
| *Acot2* | 1116.399 | -0.96875 | 0.274441 | 17.58396 | 0.000536 |
| *4930534B04Rik* | 153.7148 | -0.96228 | 0.409324 | 9.112306 | 0.027834 |
| *mt-tRNA-Glu* | 9767.831 | -0.961 | 0.226 | 22.47782 | 5.19E-05 |
| *Plekhh1* | 86.35217 | -0.95925 | 0.468883 | 8.22515 | 0.041581 |
| *Adhfe1* | 351.7776 | -0.959 | 0.350725 | 7.963043 | 0.046782 |
| *Rbfox1* | 522.6653 | -0.95669 | 0.27782 | 14.05577 | 0.00283 |
| *2310050B05Rik* | 648.339 | -0.95448 | 0.257951 | 13.4812 | 0.003704 |
| *Klhl24* | 1672.302 | -0.94786 | 0.207919 | 23.99224 | 2.51E-05 |
| *Tmod4* | 304.7769 | -0.94691 | 0.285541 | 18.63727 | 0.000325 |
| *Klhdc1* | 279.0484 | -0.94496 | 0.291889 | 12.73687 | 0.005242 |
| *AK170106* | 1502.063 | -0.94424 | 0.247739 | 18.04408 | 0.000431 |
| *Rpl3l* | 2905.92 | -0.93802 | 0.21427 | 29.70779 | 1.59E-06 |
| *Ift81* | 749.459 | -0.9308 | 0.274026 | 19.29717 | 0.000237 |
| *AK041267* | 459.585 | -0.93008 | 0.267244 | 15.90589 | 0.001185 |
| *Atp1a2* | 6224.782 | -0.92472 | 0.208702 | 24.6242 | 1.85E-05 |
| *2310010M20Rik* | 197.6245 | -0.9245 | 0.369934 | 8.18102 | 0.042415 |
| *Kcna5* | 295.6332 | -0.92377 | 0.287358 | 12.6896 | 0.005358 |
| *Auh* | 891.2738 | -0.92342 | 0.239929 | 17.39002 | 0.000587 |
| *Fzd4* | 188.4929 | -0.92287 | 0.361414 | 9.709733 | 0.021202 |
| *mt-12SrRNA* | 14650.24 | -0.92032 | 0.208217 | 31.18717 | 7.76E-07 |
| *Car4* | 512.5835 | -0.91829 | 0.257345 | 12.66578 | 0.005418 |
| *Pln* | 40567.54 | -0.90533 | 0.185009 | 27.20998 | 5.32E-06 |
| *Pnrc1* | 1386.141 | -0.90238 | 0.236271 | 15.97405 | 0.001148 |
| *Ppm1k* | 1001.556 | -0.89502 | 0.253288 | 14.02161 | 0.002876 |
| *Cd24a* | 60.3065 | -0.89026 | 0.676793 | 8.4295 | 0.037921 |
| *Hopx* | 2391.251 | -0.88929 | 0.223893 | 22.61658 | 4.85E-05 |
| *Nampt* | 2680.978 | -0.88757 | 0.246766 | 17.90545 | 0.00046 |
| *Cecr2* | 213.9501 | -0.8859 | 0.320135 | 8.599448 | 0.035119 |
| *Rbp7* | 267.1479 | -0.88446 | 0.313567 | 9.40113 | 0.024407 |
| *Yipf7* | 1008.778 | -0.87347 | 0.222029 | 22.95638 | 4.12E-05 |
| *Art1* | 1696.183 | -0.87329 | 0.260093 | 15.4766 | 0.001452 |
| *mt-tRNA-Asn* | 820.7084 | -0.87051 | 0.238063 | 17.97242 | 0.000446 |
| *Pdpr* | 197.5815 | -0.86941 | 0.34275 | 8.91486 | 0.030445 |
| *mt-Nd1* | 309518.3 | -0.86715 | 0.186986 | 27.50434 | 4.61E-06 |
| *Gm19277* | 82.11621 | -0.86465 | 0.588838 | 9.328524 | 0.025227 |
| *mt-Cytb* | 224076.8 | -0.86242 | 0.214317 | 17.80304 | 0.000483 |
| *Lrrc15* | 621.0616 | -0.86106 | 0.326048 | 10.64028 | 0.013839 |
| *Irs1* | 637.2931 | -0.85968 | 0.319689 | 9.896207 | 0.019469 |
| *mt-tRNA-Pro* | 320.5743 | -0.85725 | 0.31946 | 9.834654 | 0.020025 |
| *Bckdha* | 2424.422 | -0.85449 | 0.248254 | 14.46208 | 0.002339 |
| *Ppip5k2* | 702.5216 | -0.84525 | 0.295187 | 13.43214 | 0.003789 |
| *D8Ertd82e* | 205.6753 | -0.84312 | 0.347722 | 7.826834 | 0.049729 |
| *Tmem143* | 1158.136 | -0.84129 | 0.258734 | 12.39492 | 0.006146 |
| *Rtn2* | 512.5159 | -0.84044 | 0.308182 | 11.334 | 0.01005 |
| *Idh2* | 23281.87 | -0.83733 | 0.188494 | 24.50152 | 1.96E-05 |
| *B3gnt8* | 2145.842 | -0.83703 | 0.251247 | 13.32951 | 0.003976 |
| *Eci1* | 3457.438 | -0.83685 | 0.219823 | 19.5946 | 0.000206 |
| *mt-Nd5* | 149194.3 | -0.83546 | 0.180188 | 22.54558 | 5.02E-05 |
| *mt-Nd6* | 90592.07 | -0.83379 | 0.213034 | 17.09711 | 0.000675 |
| *Itpkb* | 772.6018 | -0.83172 | 0.257441 | 13.37788 | 0.003887 |
| *Lpl* | 40387.92 | -0.82683 | 0.212547 | 20.24641 | 0.000151 |
| *Sesn1* | 1743.493 | -0.82659 | 0.247125 | 12.40134 | 0.006127 |
| *AK155917* | 19548.4 | -0.82292 | 0.214088 | 22.72266 | 4.61E-05 |
| *Hfe2* | 2198.837 | -0.82171 | 0.224583 | 18.25921 | 0.000389 |
| *Rasgrp3* | 579.0905 | -0.8217 | 0.244579 | 13.85706 | 0.003106 |
| *Acadm* | 8441.602 | -0.81933 | 0.215608 | 18.83431 | 0.000296 |
| *Aldh6a1* | 1579.689 | -0.81648 | 0.261195 | 11.92363 | 0.007649 |
| *Arhgap20* | 402.5887 | -0.80781 | 0.284202 | 9.490188 | 0.023436 |
| *A630019I02Rik* | 600.8625 | -0.80776 | 0.238553 | 13.52318 | 0.003632 |
| *Pcdh12* | 343.6003 | -0.80354 | 0.28136 | 9.607356 | 0.022216 |
| *Ttll1* | 611.2944 | -0.80093 | 0.275146 | 16.72996 | 0.000803 |
| *mt-Co1* | 749275.8 | -0.80034 | 0.211534 | 15.8654 | 0.001208 |
| *Lyrm5* | 836.7774 | -0.79985 | 0.22277 | 13.8794 | 0.003074 |
| *mt-tRNA-Tyr* | 1714.568 | -0.79965 | 0.231345 | 17.00967 | 0.000704 |
| *mt-Nd4* | 145579.9 | -0.7986 | 0.181289 | 22.74636 | 4.56E-05 |
| *Gramd1b* | 121.4956 | -0.79577 | 0.389407 | 8.491616 | 0.036872 |
| *Pde7a* | 507.932 | -0.79544 | 0.288784 | 11.55825 | 0.00906 |
| *Etfdh* | 5753.995 | -0.79522 | 0.222654 | 17.19228 | 0.000645 |
| *Agtr1a* | 423.773 | -0.7926 | 0.270168 | 10.21542 | 0.016821 |
| *C1qtnf9* | 442.9422 | -0.79173 | 0.272433 | 8.654211 | 0.03426 |
| *Acaa2* | 11796.01 | -0.79003 | 0.221502 | 21.40969 | 8.65E-05 |
| *Nudt7* | 332.8903 | -0.7893 | 0.27305 | 8.821927 | 0.031755 |
| *Sult1a1* | 393.1608 | -0.78876 | 0.2969 | 8.477656 | 0.037106 |
| *Abhd1* | 497.2822 | -0.78717 | 0.272137 | 12.40871 | 0.006107 |
| *Cpt2* | 3045.407 | -0.78271 | 0.211715 | 17.7243 | 0.000501 |
| *Ivd* | 2467.646 | -0.77884 | 0.277679 | 9.919913 | 0.019259 |
| *mmu-mir-805* | 319.2241 | -0.7779 | 0.28492 | 10.13622 | 0.017443 |
| *Ech1* | 12463.23 | -0.77761 | 0.217509 | 21.52313 | 8.20E-05 |
| *Kcnj2* | 457.9264 | -0.77746 | 0.291165 | 8.271672 | 0.040718 |
| *Msrb2* | 492.4346 | -0.77613 | 0.275761 | 11.34578 | 0.009996 |
| *Cox8b* | 1646.275 | -0.77407 | 0.215169 | 19.15558 | 0.000254 |
| *mt-tRNA-Cys* | 1240.141 | -0.77118 | 0.233198 | 15.76917 | 0.001264 |
| *Plxnb1* | 798.3651 | -0.77047 | 0.322066 | 10.06132 | 0.018052 |
| *Myl3* | 39838.05 | -0.76939 | 0.193778 | 20.19242 | 0.000155 |
| *Hrc* | 12980.29 | -0.7693 | 0.215453 | 17.62401 | 0.000526 |
| *Lrrc4b* | 661.275 | -0.76795 | 0.268532 | 8.600563 | 0.035101 |
| *Rps6ka2* | 542.7139 | -0.76587 | 0.246105 | 10.23649 | 0.016659 |
| *Fam131a* | 274.0099 | -0.76267 | 0.304732 | 8.352475 | 0.039262 |
| *mt-tRNA-Trp* | 335.3552 | -0.76156 | 0.282106 | 12.04021 | 0.007247 |
| *Eepd1* | 280.7036 | -0.7613 | 0.2914 | 7.977004 | 0.046489 |
| *mt-tRNA-SerchrM-* | 676.381 | -0.75844 | 0.28 | 8.566329 | 0.035649 |
| *Cpox* | 397.3854 | -0.75833 | 0.263527 | 8.819244 | 0.031793 |
| *Nnt* | 5178.847 | -0.75774 | 0.214044 | 15.94377 | 0.001164 |
| *Ccdc21* | 613.3148 | -0.75767 | 0.236009 | 13.96573 | 0.002952 |
| *Adck3* | 7070.973 | -0.75604 | 0.215862 | 15.19679 | 0.001656 |
| *Fhl2* | 14928.1 | -0.75437 | 0.224834 | 16.93286 | 0.00073 |
| *Slc25a42* | 1254.268 | -0.75107 | 0.274801 | 9.142467 | 0.027455 |
| *Bckdhb* | 387.6949 | -0.74927 | 0.294667 | 8.26906 | 0.040766 |
| *Decr1* | 4164.832 | -0.74382 | 0.211185 | 15.32609 | 0.001558 |
| *Ephx2* | 2864.694 | -0.74331 | 0.238684 | 12.25085 | 0.006571 |
| *Klhl23* | 597.5735 | -0.74199 | 0.241134 | 11.22916 | 0.010549 |
| *Fabp3* | 10010.85 | -0.73953 | 0.19073 | 21.04005 | 0.000103 |
| *Mut* | 1478.153 | -0.73707 | 0.232872 | 12.55861 | 0.005695 |
| *Hadha* | 14588.97 | -0.73656 | 0.232808 | 13.81504 | 0.003168 |
| *Kdr* | 1529.003 | -0.73609 | 0.21751 | 13.45852 | 0.003743 |
| *Tesc* | 1173.612 | -0.73451 | 0.240644 | 13.38052 | 0.003882 |
| *Armcx4* | 312.0214 | -0.73371 | 0.327692 | 8.091907 | 0.04415 |
| *Pfkfb2* | 1452.857 | -0.73341 | 0.219757 | 15.63482 | 0.001347 |
| *1810011O10Rik* | 474.1309 | -0.73131 | 0.26505 | 8.34379 | 0.039416 |
| *Cox7a1* | 1819.288 | -0.7294 | 0.223453 | 16.15244 | 0.001055 |
| *Gbas* | 4119.371 | -0.7228 | 0.198119 | 17.71548 | 0.000503 |
| *mt-tRNA-Ile* | 3676.235 | -0.72026 | 0.264053 | 16.93203 | 0.00073 |
| *Rxrg* | 988.4161 | -0.71631 | 0.255278 | 12.80586 | 0.005076 |
| *Inadl* | 274.6383 | -0.71532 | 0.303945 | 8.404816 | 0.038346 |
| *Rtn4r* | 183.4034 | -0.7148 | 0.414884 | 7.927785 | 0.047528 |
| *Fyco1* | 3768.874 | -0.71424 | 0.217429 | 12.73527 | 0.005246 |
| *Ndufa3* | 1264.033 | -0.71352 | 0.214973 | 16.27922 | 0.000994 |
| *Hadhb* | 13330.76 | -0.71226 | 0.203117 | 15.70562 | 0.001303 |
| *Acss1* | 4393.856 | -0.71189 | 0.239324 | 12.7621 | 0.00518 |
| *Pcca* | 799.2008 | -0.71116 | 0.24999 | 9.793173 | 0.020409 |
| *Itga11* | 52.31078 | -0.71077 | 0.57589 | 14.25394 | 0.002579 |
| *Tecr* | 4719.215 | -0.7096 | 0.206241 | 16.9409 | 0.000727 |
| *Cacna1c* | 1982.308 | -0.70939 | 0.260876 | 10.30971 | 0.016109 |
| *Slc25a20* | 1911.11 | -0.70718 | 0.224911 | 11.82934 | 0.007991 |
| *Ndufs6* | 1727.434 | -0.70609 | 0.213561 | 14.57424 | 0.002219 |
| *Mlycdchr8+* | 1741.17 | -0.70536 | 0.255262 | 10.82264 | 0.012725 |
| *Gcdh* | 678.7679 | -0.70336 | 0.2767 | 8.318481 | 0.039868 |
| *Dhrs3* | 1144.489 | -0.70255 | 0.254098 | 7.870331 | 0.048769 |
| *mt-tRNA-Val* | 171.1677 | -0.70201 | 0.38709 | 8.717493 | 0.033293 |
| *Myl2* | 30573.93 | -0.70184 | 0.1962 | 18.53677 | 0.000341 |
| *Kif26a* | 351.0325 | -0.70163 | 0.273171 | 8.554928 | 0.035833 |
| *AK201505* | 349.8024 | -0.70104 | 0.267515 | 9.171446 | 0.027096 |
| *Asb11* | 1879.786 | -0.6973 | 0.214525 | 15.74773 | 0.001277 |
| *Pdk2* | 9074.698 | -0.69692 | 0.259522 | 8.745473 | 0.032874 |
| *Vldlr* | 4803.237 | -0.696 | 0.214387 | 13.56974 | 0.003553 |
| *Acat1* | 5285.145 | -0.69315 | 0.208502 | 14.67342 | 0.002118 |
| *AK085891* | 334.1626 | -0.69217 | 0.296079 | 8.586835 | 0.03532 |
| *Klhl7* | 732.1447 | -0.69083 | 0.236869 | 12.4642 | 0.005951 |
| *Mlycdchr8-* | 1516.691 | -0.69017 | 0.254738 | 12.08173 | 0.007108 |
| *Echs1* | 2669.956 | -0.68854 | 0.212778 | 15.82102 | 0.001234 |
| *Gpr116* | 3293.352 | -0.68773 | 0.196718 | 14.5653 | 0.002228 |
| *Fam134b* | 2043.843 | -0.68762 | 0.218802 | 10.94323 | 0.012037 |
| *Gpcpd1* | 1767.579 | -0.68744 | 0.230882 | 13.53918 | 0.003604 |
| *Actr3b* | 507.5456 | -0.686 | 0.270054 | 11.28751 | 0.010269 |
| *Tnfaip8* | 204.5293 | -0.68535 | 0.318015 | 7.97077 | 0.04662 |
| *D9Ertd402e* | 541.9687 | -0.68426 | 0.241606 | 12.53858 | 0.005749 |
| *Hsdl2* | 1845.843 | -0.68391 | 0.209143 | 12.73865 | 0.005237 |
| *Aqp1* | 7070.694 | -0.68297 | 0.195265 | 20.96902 | 0.000107 |
| *mt-tRNA-Gln* | 2023.88 | -0.67976 | 0.269966 | 15.53643 | 0.001411 |
| *0610009O20Rik* | 2096.231 | -0.67895 | 0.243799 | 8.918864 | 0.030389 |
| *Lclat1* | 1658.886 | -0.67652 | 0.211593 | 12.69813 | 0.005337 |
| *D16H22S680E* | 2426.186 | -0.67347 | 0.217741 | 10.49616 | 0.014787 |
| *Zfp46* | 413.9506 | -0.67198 | 0.267272 | 8.575495 | 0.035501 |
| *Hemk1* | 624.7931 | -0.66854 | 0.236853 | 10.13449 | 0.017457 |
| *Slc2a4* | 5134.52 | -0.66405 | 0.222228 | 11.5565 | 0.009068 |
| *Sdhc* | 4478.272 | -0.66066 | 0.193585 | 15.62193 | 0.001355 |
| *Aldh5a1* | 426.2098 | -0.65947 | 0.284194 | 7.926717 | 0.047551 |
| *Dnajc15* | 383.4404 | -0.65861 | 0.271145 | 7.994597 | 0.046124 |
| *Oxct1* | 16404.06 | -0.656 | 0.198808 | 14.31686 | 0.002504 |
| *Coq9* | 5643.645 | -0.6557 | 0.205668 | 13.93792 | 0.002991 |
| *Aplnr* | 665.8732 | -0.65544 | 0.276381 | 18.27328 | 0.000386 |
| *Pnpla2* | 8416.284 | -0.65512 | 0.249594 | 8.744836 | 0.032883 |
| *Asb10* | 2053.488 | -0.65484 | 0.215722 | 10.68845 | 0.013536 |
| *Psme1* | 1705.578 | -0.65414 | 0.209381 | 12.56877 | 0.005668 |
| *Adk* | 1808.062 | -0.65343 | 0.212236 | 13.33759 | 0.003961 |
| *Sbk1* | 1948.3 | -0.65284 | 0.210342 | 13.67106 | 0.003389 |
| *Etfb* | 4662.893 | -0.65136 | 0.20498 | 18.18643 | 0.000403 |
| *Fech* | 2217.183 | -0.65045 | 0.221551 | 8.821189 | 0.031765 |
| *mt-tRNA-Leu* | 2525.759 | -0.65022 | 0.222849 | 14.6522 | 0.002139 |
| *Mlf1* | 849.0498 | -0.65006 | 0.246633 | 9.36841 | 0.024773 |
| *Ldhb* | 19139.68 | -0.65002 | 0.194605 | 16.77083 | 0.000788 |
| *Atp5g3* | 9178.249 | -0.64945 | 0.193293 | 16.94015 | 0.000727 |
| *Aldh9a1* | 637.6792 | -0.64916 | 0.264182 | 10.56549 | 0.014323 |
| *3425401B19Rik* | 1325.424 | -0.64747 | 0.213169 | 13.71547 | 0.003319 |
| *Gpam* | 1019.733 | -0.64722 | 0.228749 | 15.0001 | 0.001817 |
| *Trp53i11* | 846.9753 | -0.64708 | 0.221712 | 9.373721 | 0.024713 |
| *Map3k5* | 545.8643 | -0.64532 | 0.264181 | 9.180453 | 0.026985 |
| *Acsl1* | 7066.982 | -0.64316 | 0.202609 | 12.89101 | 0.004878 |
| *Sucla2* | 4964.968 | -0.64058 | 0.202431 | 14.72253 | 0.00207 |
| *Tmem38a* | 7091.828 | -0.64055 | 0.19129 | 16.42538 | 0.000928 |
| *mt-tRNA-Ala* | 485.5775 | -0.63874 | 0.269521 | 10.66898 | 0.013657 |
| *Smoc2* | 1251.384 | -0.63791 | 0.211219 | 18.07787 | 0.000424 |
| *Bcat2* | 2519.05 | -0.63656 | 0.234337 | 11.78699 | 0.00815 |
| *Gpr157* | 638.1379 | -0.63629 | 0.251929 | 8.61291 | 0.034906 |
| *Ckm* | 24127.43 | -0.63578 | 0.205705 | 12.35214 | 0.006269 |
| *Cd300lg* | 1525.317 | -0.63572 | 0.20402 | 11.90224 | 0.007726 |
| *Sdhb* | 8637.153 | -0.63535 | 0.18672 | 17.43293 | 0.000576 |
| *Acads* | 2843.259 | -0.63501 | 0.210316 | 11.97303 | 0.007476 |
| *Mtfp1* | 1915.208 | -0.63022 | 0.21157 | 13.26411 | 0.004099 |
| *Etfa* | 3972.487 | -0.62984 | 0.198887 | 13.63251 | 0.003451 |
| *Ppa2* | 656.5594 | -0.62873 | 0.237541 | 8.930389 | 0.030231 |
| *Rgs5* | 1452.011 | -0.62797 | 0.219189 | 11.94108 | 0.007588 |
| *Dhrs7c* | 1569.565 | -0.62761 | 0.232446 | 11.39232 | 0.009783 |
| *Herpud1* | 2758.871 | -0.62554 | 0.254525 | 8.311632 | 0.039992 |
| *A930016O22Rik* | 12642.52 | -0.62427 | 0.20614 | 12.80695 | 0.005073 |
| *Ablim1* | 4803.141 | -0.62351 | 0.209236 | 10.69206 | 0.013513 |
| *Mgst3* | 3325.331 | -0.62172 | 0.192556 | 15.77611 | 0.00126 |
| *Pla2g12a* | 413.838 | -0.61907 | 0.282979 | 8.510309 | 0.036562 |
| *Prdx5* | 1778.861 | -0.61808 | 0.213406 | 11.83897 | 0.007956 |
| *Retsat* | 731.2761 | -0.61742 | 0.26923 | 8.7365 | 0.033007 |
| *Txlnb* | 4757.431 | -0.61731 | 0.210088 | 8.993195 | 0.029381 |
| *Ndufv3* | 1098.251 | -0.61692 | 0.224867 | 10.76581 | 0.013062 |
| *Lrrc39* | 1499.569 | -0.61657 | 0.20369 | 11.76558 | 0.008231 |
| *Uqcrfs1* | 8329.067 | -0.61485 | 0.192677 | 14.76143 | 0.002032 |
| *Ephb3* | 343.9242 | -0.61454 | 0.272128 | 8.089925 | 0.044189 |
| *Macrod1* | 1550.245 | -0.61279 | 0.218006 | 12.18125 | 0.006787 |
| *Ndufa9* | 3140.857 | -0.61003 | 0.1959 | 13.82864 | 0.003148 |
| *Acadsb* | 1306.175 | -0.60777 | 0.215068 | 8.743101 | 0.032909 |
| *Cs* | 17766.19 | -0.60734 | 0.221928 | 9.076794 | 0.028287 |
| *Prepl* | 1196.68 | -0.60678 | 0.229899 | 7.92971 | 0.047487 |
| *Hdac9* | 196.8255 | -0.60508 | 0.32709 | 8.754168 | 0.032744 |
| *Rab12* | 1811.596 | -0.6013 | 0.220804 | 9.830869 | 0.02006 |
| *Dhrs4* | 887.8143 | -0.60088 | 0.218921 | 10.36371 | 0.015715 |
| *Car14* | 2108.118 | -0.59897 | 0.231091 | 10.53776 | 0.014507 |
| *Cyc1* | 10910.3 | -0.5982 | 0.18611 | 14.98116 | 0.001833 |
| *Cox6a2* | 5256.192 | -0.59752 | 0.196125 | 17.30326 | 0.000612 |
| *Sdha* | 14656.03 | -0.59471 | 0.197848 | 12.90607 | 0.004844 |
| *Ndufs2* | 11437.18 | -0.59345 | 0.19681 | 12.93605 | 0.004777 |
| *2810405K02Rik* | 446.7886 | -0.5925 | 0.262946 | 9.134614 | 0.027554 |
| *Tnni3k* | 1514.333 | -0.59112 | 0.247932 | 7.894213 | 0.048249 |
| *Uqcrq* | 2213.927 | -0.59096 | 0.199476 | 14.79151 | 0.002004 |
| *Ppif* | 3147.1 | -0.5902 | 0.207131 | 11.76964 | 0.008215 |
| *Uqcrc1* | 26525.42 | -0.58982 | 0.205543 | 13.86904 | 0.003089 |
| *Grb14* | 1994.533 | -0.58953 | 0.205482 | 9.660706 | 0.021682 |
| *Mgll* | 5623.524 | -0.58884 | 0.199129 | 10.24361 | 0.016605 |
| *Tecrl* | 826.6064 | -0.58838 | 0.225814 | 8.033172 | 0.045331 |
| *Ndufa5* | 1427.186 | -0.58635 | 0.212832 | 13.47337 | 0.003717 |
| *Plin5* | 2780.363 | -0.58539 | 0.254824 | 9.689568 | 0.021398 |
| *H2-Q6* | 112.4416 | -0.58514 | 0.53795 | 11.32961 | 0.010071 |
| *Ckmt2* | 15804.98 | -0.58463 | 0.201153 | 16.24753 | 0.001009 |
| *Coq10a* | 2151.112 | -0.58417 | 0.201699 | 13.09043 | 0.004445 |
| *Bsg* | 13078.54 | -0.58388 | 0.208794 | 10.83887 | 0.01263 |
| *Sgcg* | 2924.509 | -0.58204 | 0.193063 | 14.70564 | 0.002086 |
| *Ndufab1* | 1583.258 | -0.58067 | 0.206377 | 9.930625 | 0.019165 |
| *Atp5e* | 849.6731 | -0.58037 | 0.230447 | 9.844905 | 0.019932 |
| *Col6a2* | 2460.094 | 0.581187 | 0.232937 | 29.02355 | 2.21E-06 |
| *Stat3* | 2367.677 | 0.58218 | 0.203866 | 9.37167 | 0.024737 |
| *Hyou1* | 961.0055 | 0.585731 | 0.218026 | 10.09852 | 0.017747 |
| *Copg* | 1463.292 | 0.588944 | 0.203166 | 11.55253 | 0.009084 |
| *Lrrc32* | 431.2343 | 0.589845 | 0.282022 | 8.214698 | 0.041777 |
| *Zyx* | 2018.777 | 0.59181 | 0.206486 | 12.24012 | 0.006604 |
| *Sec22b* | 448.503 | 0.59421 | 0.259044 | 8.569775 | 0.035593 |
| *AK196308* | 927.735 | 0.600837 | 0.222993 | 14.45086 | 0.002351 |
| *Angptl2* | 1649.377 | 0.602846 | 0.251212 | 8.044738 | 0.045096 |
| *Cdv3* | 1778.039 | 0.608888 | 0.235665 | 8.054773 | 0.044893 |
| *Gfpt2* | 389.8397 | 0.613354 | 0.274756 | 9.079427 | 0.028253 |
| *Icam1* | 326.2681 | 0.617239 | 0.290703 | 10.71081 | 0.013397 |
| *Chpf* | 719.969 | 0.617703 | 0.235735 | 16.24429 | 0.00101 |
| *Flna* | 3673.512 | 0.621624 | 0.225389 | 8.194614 | 0.042156 |
| *Nudt4* | 3907.121 | 0.624727 | 0.231349 | 11.57347 | 0.008997 |
| *Mvp* | 934.9596 | 0.62629 | 0.227788 | 8.378108 | 0.038811 |
| *Pdia6* | 1198.391 | 0.629747 | 0.240829 | 12.83835 | 0.005 |
| *Oaf* | 217.2078 | 0.631009 | 0.340021 | 8.874946 | 0.031001 |
| *Arhgap31* | 966.5437 | 0.632696 | 0.26882 | 9.626577 | 0.022022 |
| *Cyp2e1* | 70.00966 | 0.634946 | 0.636582 | 10.57086 | 0.014288 |
| *Tuba1b* | 1866.26 | 0.635104 | 0.207593 | 16.58759 | 0.000859 |
| *Rfxank* | 515.0753 | 0.638367 | 0.263392 | 9.500128 | 0.02333 |
| *Col14a1* | 414.2577 | 0.638388 | 0.353468 | 32.67262 | 3.78E-07 |
| *Tbrg1* | 631.2005 | 0.639697 | 0.251848 | 9.394717 | 0.024478 |
| *Dlgap4* | 876.6136 | 0.639716 | 0.224306 | 9.982397 | 0.018716 |
| *Tgm2* | 5968.082 | 0.640887 | 0.220751 | 10.79315 | 0.012899 |
| *Fam129b* | 783.8731 | 0.642658 | 0.229343 | 10.43223 | 0.015228 |
| *Ctsz* | 702.7064 | 0.643252 | 0.282011 | 8.71517 | 0.033328 |
| *Col6a1* | 2842.847 | 0.643373 | 0.251655 | 27.12629 | 5.54E-06 |
| *Mmp2* | 1907.274 | 0.643743 | 0.23598 | 57.7983 | 1.74E-12 |
| *Trf* | 617.1365 | 0.644358 | 0.24876 | 13.02492 | 0.004583 |
| *AK217614* | 902.5917 | 0.645433 | 0.263225 | 8.304381 | 0.040123 |
| *Lyz2* | 1834.445 | 0.646077 | 0.20884 | 28.01031 | 3.61E-06 |
| *Csf2rb* | 65.53002 | 0.647744 | 0.509859 | 13.14046 | 0.004342 |
| *Xbp1* | 1199.677 | 0.649855 | 0.248227 | 9.621305 | 0.022075 |
| *Fat1* | 353.8778 | 0.65444 | 0.281534 | 9.290888 | 0.025663 |
| *Acta2* | 4483.772 | 0.656265 | 0.248813 | 13.50064 | 0.00367 |
| *Mical2* | 1781.469 | 0.65937 | 0.199127 | 12.21622 | 0.006678 |
| *Igfbp7* | 2458.258 | 0.66069 | 0.252819 | 14.56634 | 0.002227 |
| *Xirp1* | 5643.537 | 0.66145 | 0.234295 | 13.5856 | 0.003527 |
| *Tuba1a* | 1518.519 | 0.662088 | 0.226356 | 13.89845 | 0.003047 |
| *Colec12* | 505.555 | 0.662174 | 0.266693 | 9.927709 | 0.019191 |
| *Col20a1* | 82.36178 | 0.663857 | 0.451217 | 10.86655 | 0.01247 |
| *Tubb5* | 2579.282 | 0.669828 | 0.207352 | 13.43651 | 0.003782 |
| *Col5a1* | 993.2353 | 0.671496 | 0.276625 | 25.77553 | 1.06E-05 |
| *Loxl1* | 909.9947 | 0.671752 | 0.240527 | 26.9259 | 6.10E-06 |
| *Morf4l2* | 818.9343 | 0.677662 | 0.266432 | 8.245175 | 0.041207 |
| *Igf1* | 227.4828 | 0.67806 | 0.363416 | 7.970132 | 0.046633 |
| *Manf* | 295.4652 | 0.685348 | 0.288612 | 11.19461 | 0.010719 |
| *Eif5* | 1907.41 | 0.68646 | 0.246181 | 9.643913 | 0.021849 |
| *Numbl* | 165.4779 | 0.686895 | 0.349292 | 11.84052 | 0.00795 |
| *Sec61a1* | 1007.981 | 0.693674 | 0.245654 | 10.15798 | 0.01727 |
| *Col11a1* | 19.82896 | 0.695497 | 1.089446 | 10.07235 | 0.017961 |
| *Ctss* | 163.984 | 0.7003 | 0.346712 | 14.99115 | 0.001824 |
| *Fbln1* | 2611.118 | 0.703648 | 0.273643 | 14.42582 | 0.002379 |
| *Lrrc59* | 795.2707 | 0.705029 | 0.245671 | 9.847612 | 0.019907 |
| *Pi16* | 1584.401 | 0.709665 | 0.236548 | 25.29873 | 1.34E-05 |
| *Cfp* | 295.3442 | 0.711107 | 0.291231 | 8.564933 | 0.035671 |
| *Lrp1* | 2406.684 | 0.712115 | 0.208617 | 23.00087 | 4.04E-05 |
| *Srxn1* | 437.0925 | 0.712411 | 0.29277 | 8.161326 | 0.042793 |
| *Tgfb3* | 478.383 | 0.713635 | 0.264265 | 16.43116 | 0.000925 |
| *6430548M08Rik* | 685.3065 | 0.715118 | 0.258636 | 9.097979 | 0.028016 |
| *Thbs3* | 187.1376 | 0.719349 | 0.377296 | 12.7387 | 0.005237 |
| *Gas7* | 201.066 | 0.719797 | 0.374119 | 8.322723 | 0.039792 |
| *Pkdcc* | 980.062 | 0.728736 | 0.225985 | 13.88433 | 0.003067 |
| *AK204572* | 1685.648 | 0.729548 | 0.221238 | 16.66864 | 0.000827 |
| *Cd63* | 1780.105 | 0.731847 | 0.248317 | 9.150715 | 0.027353 |
| *Fibin* | 152.9377 | 0.732114 | 0.466897 | 10.77305 | 0.013019 |
| *Pdia4* | 575.3746 | 0.734211 | 0.254802 | 10.86815 | 0.012461 |
| *Lyve1* | 279.3733 | 0.745014 | 0.355943 | 12.97418 | 0.004693 |
| *Serpinh1* | 3163.464 | 0.746574 | 0.250202 | 10.60377 | 0.014073 |
| *AW555464* | 353.6865 | 0.747371 | 0.268615 | 9.128538 | 0.02763 |
| *AK196015* | 505.9259 | 0.748628 | 0.295642 | 8.673162 | 0.033967 |
| *Tgfb1i1* | 399.3365 | 0.751022 | 0.266589 | 9.504492 | 0.023284 |
| *Prss23* | 198.2517 | 0.755456 | 0.387432 | 8.040588 | 0.04518 |
| *Eef1a1* | 12840.49 | 0.756169 | 0.207892 | 21.55843 | 8.06E-05 |
| *C1ra* | 193.6423 | 0.759574 | 0.380057 | 8.618166 | 0.034823 |
| *Itga5* | 1623.077 | 0.762709 | 0.219584 | 13.62467 | 0.003463 |
| *Col15a1* | 1656.08 | 0.768234 | 0.287821 | 21.44181 | 8.52E-05 |
| *Tuba1c* | 685.4498 | 0.768369 | 0.256645 | 14.64968 | 0.002142 |
| *Entpd1* | 379.8255 | 0.768545 | 0.272221 | 8.059345 | 0.044801 |
| *Lsp1* | 582.2873 | 0.77328 | 0.275292 | 10.94861 | 0.012007 |
| *Kcnj5* | 1642.084 | 0.773894 | 0.279645 | 10.75527 | 0.013126 |
| *Sh3pxd2b* | 227.7787 | 0.784991 | 0.313932 | 18.37931 | 0.000367 |
| *Parp3* | 506.5925 | 0.785035 | 0.308602 | 8.110801 | 0.043776 |
| *S1pr2* | 154.3755 | 0.785365 | 0.380808 | 8.82124 | 0.031764 |
| *Myo5a* | 197.5806 | 0.785381 | 0.347942 | 8.394432 | 0.038526 |
| *Sort1* | 944.4447 | 0.787161 | 0.272581 | 17.53455 | 0.000549 |
| *Itgbl1* | 211.8524 | 0.788689 | 0.342647 | 24.70416 | 1.78E-05 |
| *Ckap4* | 714.4948 | 0.789289 | 0.251726 | 10.75897 | 0.013103 |
| *Kdelr2* | 391.8551 | 0.789726 | 0.274226 | 11.29393 | 0.010238 |
| *Ndrg4* | 1389.496 | 0.79022 | 0.213868 | 17.31528 | 0.000609 |
| *Fkbp5* | 612.4092 | 0.790424 | 0.254687 | 14.17661 | 0.002674 |
| *Pf4* | 96.69237 | 0.790798 | 0.429784 | 9.485792 | 0.023483 |
| *C3* | 1455.398 | 0.792388 | 0.239278 | 39.27237 | 1.52E-08 |
| *Scd1* | 318.6752 | 0.794338 | 0.336909 | 24.65267 | 1.83E-05 |
| *Fzd2* | 179.3984 | 0.795785 | 0.399631 | 8.746536 | 0.032858 |
| *C3ar1* | 129.2582 | 0.796922 | 0.403468 | 8.939545 | 0.030106 |
| *1810055G02Rik* | 164.3834 | 0.797043 | 0.377915 | 9.741884 | 0.020893 |
| *Plekho1* | 781.1491 | 0.7972 | 0.255486 | 12.07661 | 0.007125 |
| *Cpxm2* | 420.8243 | 0.799173 | 0.256065 | 23.32304 | 3.46E-05 |
| *Tmem39a* | 214.0672 | 0.801185 | 0.31719 | 8.348917 | 0.039325 |
| *Tspan9* | 1667.577 | 0.803731 | 0.282552 | 10.2636 | 0.016453 |
| *AK181697* | 549.012 | 0.80775 | 0.276194 | 13.08142 | 0.004464 |
| *Slc1a5* | 332.0366 | 0.808389 | 0.300806 | 12.75706 | 0.005193 |
| *Maged1* | 1823.814 | 0.809427 | 0.205809 | 19.44494 | 0.000221 |
| *Tmed3* | 364.7749 | 0.811062 | 0.279195 | 9.657133 | 0.021717 |
| *Cd9* | 443.6888 | 0.811115 | 0.264592 | 12.51504 | 0.005812 |
| *Sdc2* | 410.3171 | 0.8123 | 0.309763 | 8.921369 | 0.030355 |
| *Creld2* | 325.1403 | 0.820757 | 0.283842 | 12.25322 | 0.006564 |
| *Actn1* | 667.7526 | 0.827508 | 0.234139 | 15.4825 | 0.001447 |
| *Mettl1* | 154.8575 | 0.828977 | 0.349857 | 8.627792 | 0.034672 |
| *Mmp14* | 409.6738 | 0.831267 | 0.26284 | 26.36571 | 8.00E-06 |
| *Nab2* | 217.0076 | 0.832026 | 0.311215 | 8.753935 | 0.032748 |
| *Wisp2* | 217.6577 | 0.832505 | 0.339865 | 19.88585 | 0.000179 |
| *Adcyap1r1* | 175.9436 | 0.833417 | 0.369978 | 8.046514 | 0.04506 |
| *Pcolce* | 956.4951 | 0.835617 | 0.234252 | 17.28543 | 0.000617 |
| *Cercam* | 56.03414 | 0.835875 | 0.586177 | 9.324771 | 0.02527 |
| *Emilin1* | 582.6081 | 0.836263 | 0.27041 | 14.0662 | 0.002816 |
| *Pfkp* | 924.9549 | 0.839407 | 0.300463 | 8.510429 | 0.03656 |
| *Cspg4* | 573.0379 | 0.841486 | 0.289547 | 8.697425 | 0.033596 |
| *Uap1* | 476.5632 | 0.844113 | 0.301104 | 11.14342 | 0.010975 |
| *Vim* | 4298.5 | 0.844767 | 0.25964 | 15.51284 | 0.001427 |
| *Txndc5* | 924.1611 | 0.856039 | 0.242244 | 17.55707 | 0.000543 |
| *Gltp* | 236.3311 | 0.856416 | 0.347453 | 8.911497 | 0.030491 |
| *Dbn1* | 163.467 | 0.85756 | 0.394814 | 9.803335 | 0.020314 |
| *Arfgap3* | 253.986 | 0.859806 | 0.311758 | 8.161503 | 0.042789 |
| *Mtap1a* | 549.5017 | 0.861332 | 0.295487 | 14.30982 | 0.002512 |
| *Ctsk* | 111.4591 | 0.864473 | 0.5091 | 14.13689 | 0.002725 |
| *Pprc1* | 306.6806 | 0.864746 | 0.289033 | 10.30575 | 0.016138 |
| *Frem1* | 19.84312 | 0.869173 | 0.962226 | 8.90433 | 0.03059 |
| *Nr4a1* | 326.2515 | 0.869743 | 0.326183 | 10.18172 | 0.017083 |
| *Pla1a* | 242.4601 | 0.87199 | 0.404515 | 7.823348 | 0.049807 |
| *Ctsh* | 206.3518 | 0.873057 | 0.330589 | 8.719215 | 0.033267 |
| *Antxr1* | 254.1032 | 0.874481 | 0.380752 | 16.22535 | 0.00102 |
| *Enah* | 1310.18 | 0.877154 | 0.235181 | 15.3364 | 0.001551 |
| *Clic1* | 486.5461 | 0.877693 | 0.302199 | 11.38899 | 0.009798 |
| *Serpinf1* | 640.9817 | 0.878571 | 0.314882 | 22.65759 | 4.76E-05 |
| *Hspa1l* | 285.637 | 0.888568 | 0.309258 | 13.71314 | 0.003323 |
| *Dnm1* | 318.1834 | 0.890471 | 0.340721 | 8.511638 | 0.036541 |
| *Rpl3* | 840.372 | 0.891791 | 0.252712 | 15.96462 | 0.001153 |
| *Ecm1* | 1040.448 | 0.892469 | 0.266191 | 20.68908 | 0.000122 |
| *F2r* | 501.0186 | 0.892518 | 0.266113 | 15.82859 | 0.00123 |
| *Bgn* | 8356.872 | 0.893301 | 0.248256 | 31.16495 | 7.85E-07 |
| *Fn1* | 1007.405 | 0.898787 | 0.247858 | 43.04676 | 2.41E-09 |
| *Ctla2a* | 381.07 | 0.899168 | 0.330753 | 13.28563 | 0.004058 |
| *Srm* | 282.9122 | 0.913205 | 0.315401 | 10.04147 | 0.018217 |
| *AK214641* | 762.8639 | 0.925649 | 0.323742 | 10.82212 | 0.012728 |
| *S100a11* | 242.0397 | 0.926368 | 0.355192 | 8.282908 | 0.040513 |
| *Gja5* | 263.7649 | 0.926598 | 0.301275 | 13.84134 | 0.003129 |
| *Col16a1* | 257.6018 | 0.933151 | 0.383567 | 16.36004 | 0.000957 |
| *Fzd7* | 151.2842 | 0.933313 | 0.360436 | 12.78053 | 0.005136 |
| *Skap2* | 102.5473 | 0.935929 | 0.410769 | 8.400706 | 0.038417 |
| *AI464131* | 291.0292 | 0.947802 | 0.314905 | 13.16845 | 0.004286 |
| *Gsg1l* | 304.5702 | 0.948889 | 0.320877 | 8.794145 | 0.032157 |
| *Prkag2* | 440.6257 | 0.952281 | 0.288961 | 18.69514 | 0.000316 |
| *Ccl9* | 162.768 | 0.955672 | 0.356419 | 13.54685 | 0.003592 |
| *Mpeg1* | 117.3097 | 0.956077 | 0.400362 | 23.44803 | 3.26E-05 |
| *Sfrp2* | 58.0249 | 0.962154 | 0.741499 | 11.53965 | 0.009139 |
| *Olfml3* | 415.7622 | 0.9635 | 0.308794 | 12.83631 | 0.005004 |
| *Fstl1* | 2203.404 | 0.96464 | 0.275484 | 24.52122 | 1.94E-05 |
| *Adamts2* | 606.5599 | 0.970388 | 0.275128 | 33.67419 | 2.32E-07 |
| *Igfbp4* | 2035.41 | 0.971604 | 0.257855 | 19.67417 | 0.000198 |
| *Tmem176b* | 813.2216 | 0.972046 | 0.261241 | 19.49915 | 0.000216 |
| *Slc5a3* | 119.0491 | 0.982121 | 0.388001 | 10.34895 | 0.015821 |
| *Plaur* | 36.66406 | 0.984234 | 0.66283 | 7.858665 | 0.049025 |
| *Dpysl3* | 201.3186 | 0.985963 | 0.387245 | 8.605737 | 0.035019 |
| *1700003D09Rik* | 17.12647 | 0.996478 | 1.076907 | 11.40266 | 0.009736 |
| *Slc7a5* | 243.8267 | 1.011083 | 0.346279 | 10.72704 | 0.013297 |
| *Tubb6* | 512.5421 | 1.018926 | 0.301802 | 14.47927 | 0.00232 |
| *Lcn2* | 76.43662 | 1.018985 | 0.494737 | 8.33674 | 0.039542 |
| *Ccdc80* | 2473.495 | 1.026325 | 0.534239 | 8.816952 | 0.031826 |
| *Vwf* | 1453.406 | 1.032764 | 0.292603 | 18.14967 | 0.00041 |
| *Svep1* | 289.4967 | 1.036444 | 0.322538 | 30.8414 | 9.18E-07 |
| *Lbp* | 95.41389 | 1.038155 | 0.456469 | 10.72022 | 0.013339 |
| *Emp1* | 862.3012 | 1.038818 | 0.31005 | 16.85101 | 0.000758 |
| *Cytl1* | 275.5582 | 1.043512 | 0.393283 | 11.77715 | 0.008187 |
| *Renbp* | 104.4058 | 1.049292 | 0.431522 | 7.961069 | 0.046823 |
| *P4ha3* | 26.39163 | 1.054603 | 0.805207 | 10.02932 | 0.018319 |
| *Col1a2* | 4345.426 | 1.060405 | 0.269569 | 50.72081 | 5.61E-11 |
| *Fras1* | 33.94649 | 1.061492 | 0.832917 | 14.94357 | 0.001866 |
| *Rasl11b* | 589.4477 | 1.066124 | 0.290347 | 13.59123 | 0.003518 |
| *Col27a1* | 113.11 | 1.073616 | 0.456045 | 10.16863 | 0.017186 |
| *Tmem176a* | 256.0158 | 1.075897 | 0.320281 | 17.8794 | 0.000466 |
| *Anxa1* | 501.6811 | 1.088555 | 0.31928 | 19.39143 | 0.000227 |
| *Sdf2l1* | 109.1737 | 1.089986 | 0.429946 | 11.42369 | 0.009642 |
| *Sulf1* | 318.1258 | 1.090034 | 0.289074 | 21.66553 | 7.66E-05 |
| *Fzd1* | 234.543 | 1.094411 | 0.36648 | 14.94581 | 0.001864 |
| *Upp1* | 126.9025 | 1.095303 | 0.411446 | 8.579192 | 0.035442 |
| *Col5a2* | 733.7832 | 1.099525 | 0.589606 | 8.515075 | 0.036484 |
| *Bhlhe40* | 696.7832 | 1.099765 | 0.324997 | 13.17684 | 0.004269 |
| *Slc2a1* | 257.1481 | 1.109639 | 0.377272 | 11.29803 | 0.010219 |
| *Phlda3* | 210.3586 | 1.112135 | 0.372605 | 13.80098 | 0.003189 |
| *Vcam1* | 184.638 | 1.11741 | 0.40449 | 12.09544 | 0.007063 |
| *Junb* | 232.7819 | 1.118103 | 0.381225 | 9.656996 | 0.021719 |
| *Creb3l2* | 928.0967 | 1.120105 | 0.2698 | 21.68557 | 7.58E-05 |
| *Cfh* | 2226.932 | 1.12026 | 0.249555 | 39.60252 | 1.29E-08 |
| *Mbp* | 93.20696 | 1.12268 | 0.535193 | 8.260307 | 0.040927 |
| *Pamr1* | 121.3991 | 1.125954 | 0.481832 | 25.72347 | 1.09E-05 |
| *Meox1* | 202.17 | 1.127998 | 0.428273 | 11.49488 | 0.00933 |
| *Eno2* | 54.44483 | 1.129994 | 0.545767 | 8.894467 | 0.030727 |
| *Creb3l1* | 254.5601 | 1.141265 | 0.310022 | 14.41671 | 0.002389 |
| *Ccdc136* | 113.3464 | 1.142928 | 0.44751 | 9.759121 | 0.020729 |
| *Psd3* | 492.0842 | 1.147104 | 0.33056 | 18.06846 | 0.000426 |
| *Pi15* | 83.52516 | 1.149418 | 0.472718 | 11.29389 | 0.010238 |
| *Gadd45g* | 198.8622 | 1.153842 | 0.337368 | 20.30679 | 0.000147 |
| *Tsku* | 64.27251 | 1.160583 | 0.518612 | 8.469023 | 0.037251 |
| *Anxa2* | 1685.19 | 1.163019 | 0.288014 | 20.36274 | 0.000143 |
| *Rhou* | 192.2159 | 1.179773 | 0.324293 | 18.8374 | 0.000295 |
| *Pdlim4* | 151.7485 | 1.186938 | 0.461974 | 12.12041 | 0.006982 |
| *Lox* | 72.39761 | 1.188837 | 0.554216 | 8.297776 | 0.040242 |
| *S100a6* | 200.4639 | 1.197663 | 0.421193 | 8.934782 | 0.030171 |
| *Mtap1b* | 522.3027 | 1.203526 | 0.276653 | 27.74463 | 4.11E-06 |
| *Mtap6* | 72.14867 | 1.206283 | 0.487367 | 7.922388 | 0.047643 |
| *B4galt5* | 325.2514 | 1.207082 | 0.349638 | 14.18571 | 0.002663 |
| *Sbno2* | 399.3355 | 1.208211 | 0.304776 | 16.50336 | 0.000894 |
| *Tpm2* | 429.52 | 1.210015 | 0.337938 | 22.83214 | 4.38E-05 |
| *Col8a2* | 36.69793 | 1.213773 | 0.746164 | 20.01408 | 0.000169 |
| *Adam33* | 87.75966 | 1.21697 | 0.641344 | 9.71107 | 0.021189 |
| *Slit3* | 203.1232 | 1.22108 | 0.376592 | 18.00731 | 0.000438 |
| *Camk1d* | 147.6539 | 1.237552 | 0.51299 | 12.58659 | 0.005622 |
| *Col1a1* | 3032.174 | 1.239351 | 0.267123 | 79.39328 | 4.14E-17 |
| *Cyp1b1* | 129.1693 | 1.247385 | 0.397373 | 13.03475 | 0.004562 |
| *Krt8* | 13.24828 | 1.251647 | 1.175854 | 9.361424 | 0.024852 |
| *Sdc1* | 142.3943 | 1.256673 | 0.395854 | 13.26588 | 0.004096 |
| *Apc2* | 238.4086 | 1.257096 | 0.424039 | 10.84529 | 0.012593 |
| *Akr1b8* | 67.47901 | 1.262491 | 0.52416 | 10.81377 | 0.012777 |
| *Flrt1* | 35.71917 | 1.274745 | 0.691638 | 8.658987 | 0.034186 |
| *Pkhd1l1* | 90.4727 | 1.2859 | 0.471339 | 9.736045 | 0.020948 |
| *Serpine2* | 516.3592 | 1.286573 | 0.287989 | 25.40662 | 1.27E-05 |
| *Ptn* | 92.43565 | 1.287346 | 0.539533 | 42.53288 | 3.09E-09 |
| *Nkd2* | 72.93688 | 1.289746 | 0.50162 | 13.71041 | 0.003327 |
| *Pdk3* | 65.87946 | 1.293676 | 0.572057 | 7.932449 | 0.047429 |
| *Ntsr2* | 167.3985 | 1.297898 | 0.397657 | 13.98399 | 0.002927 |
| *Cacna2d3* | 29.35756 | 1.304631 | 0.827529 | 8.795359 | 0.032139 |
| *Ttc9* | 80.84239 | 1.312782 | 0.513162 | 8.273641 | 0.040682 |
| *Lsr* | 57.59833 | 1.313958 | 0.553072 | 8.93766 | 0.030131 |
| *Ccl6* | 238.8622 | 1.323281 | 0.31752 | 40.5483 | 8.15E-09 |
| *Fam46b* | 91.84063 | 1.324834 | 0.455738 | 9.016062 | 0.029078 |
| *Asns* | 186.0794 | 1.332886 | 0.334513 | 19.97315 | 0.000172 |
| *Synm* | 355.0596 | 1.33394 | 0.287959 | 26.38448 | 7.92E-06 |
| *Rcn3* | 461.887 | 1.335784 | 0.291795 | 21.89031 | 6.88E-05 |
| *Dap* | 377.7674 | 1.339328 | 0.308618 | 23.70625 | 2.88E-05 |
| *Fxyd5* | 320.732 | 1.345171 | 0.344767 | 19.46565 | 0.000219 |
| *Frzb* | 132.047 | 1.347628 | 0.436048 | 15.00439 | 0.001813 |
| *Msr1* | 44.16599 | 1.351609 | 0.612584 | 9.195341 | 0.026803 |
| *Col6a3* | 740.8394 | 1.353106 | 0.591478 | 8.708184 | 0.033433 |
| *Hp* | 75.13986 | 1.356159 | 0.571684 | 23.72388 | 2.85E-05 |
| *Capg* | 242.481 | 1.360117 | 0.409644 | 15.17915 | 0.00167 |
| *Adam8* | 34.54251 | 1.36322 | 0.69609 | 10.96392 | 0.011923 |
| *Gng2* | 101.5889 | 1.36345 | 0.600405 | 12.3679 | 0.006223 |
| *Zfp579* | 228.5925 | 1.365857 | 0.374473 | 16.79523 | 0.000779 |
| *Trim47* | 1007.463 | 1.374602 | 0.26311 | 30.65398 | 1.01E-06 |
| *Ptgis* | 321.8559 | 1.378314 | 0.307242 | 31.61877 | 6.30E-07 |
| *Igfbp6* | 301.4197 | 1.383902 | 0.330562 | 28.6265 | 2.68E-06 |
| *Prss57* | 248.6509 | 1.411355 | 0.346294 | 19.19294 | 0.000249 |
| *Il33* | 77.99659 | 1.411892 | 0.603412 | 8.876302 | 0.030982 |
| *Tmem100* | 49.93847 | 1.41513 | 0.577909 | 11.35727 | 0.009943 |
| *Gnao1* | 841.9597 | 1.428545 | 0.309899 | 33.97364 | 2.01E-07 |
| *Trim46* | 44.24265 | 1.431641 | 0.623181 | 8.954878 | 0.029897 |
| *Kif1a* | 56.96073 | 1.434481 | 0.64267 | 9.611363 | 0.022176 |
| *Fstl3* | 300.471 | 1.440848 | 0.330064 | 21.98595 | 6.57E-05 |
| *Uchl1* | 78.60883 | 1.451487 | 0.494126 | 10.77452 | 0.01301 |
| *Col3a1* | 7336.834 | 1.475759 | 0.70675 | 11.65047 | 0.008682 |
| *Ms4a6d* | 73.76483 | 1.47638 | 0.562969 | 9.445274 | 0.023921 |
| *AK014660* | 253.2037 | 1.477198 | 0.313027 | 26.78567 | 6.53E-06 |
| *Nmrk2* | 1661.293 | 1.483125 | 0.216753 | 48.46964 | 1.69E-10 |
| *Olfm1* | 200.8908 | 1.485626 | 0.393051 | 21.39227 | 8.73E-05 |
| *Clu* | 18663.77 | 1.487694 | 0.207965 | 61.38251 | 2.98E-13 |
| *Pdgfc* | 70.08554 | 1.491403 | 0.513461 | 11.59871 | 0.008892 |
| *Dpep2* | 22.02488 | 1.500682 | 0.877641 | 10.35611 | 0.015769 |
| *Aebp1* | 612.3714 | 1.500833 | 0.286825 | 43.42289 | 2.00E-09 |
| *Cilp* | 478.0154 | 1.514862 | 0.810927 | 17.38025 | 0.00059 |
| *Mthfd2* | 66.76594 | 1.520154 | 0.531772 | 8.95895 | 0.029842 |
| *Sytl2* | 41.86115 | 1.521271 | 0.843401 | 9.791623 | 0.020423 |
| *Fam114a1* | 245.5957 | 1.522751 | 0.321007 | 29.25193 | 1.98E-06 |
| *Pam* | 17356.07 | 1.525518 | 0.260651 | 51.56961 | 3.70E-11 |
| *Cnn1* | 138.1388 | 1.53225 | 0.452597 | 17.09939 | 0.000674 |
| *Gpx3* | 8583.469 | 1.545048 | 0.608384 | 11.47078 | 0.009434 |
| *Serpina3g* | 40.34393 | 1.562594 | 0.636095 | 13.79698 | 0.003195 |
| *Lrrn2* | 187.2316 | 1.587521 | 0.437257 | 20.06536 | 0.000165 |
| *Cd300lb* | 23.6835 | 1.593919 | 0.822021 | 13.38223 | 0.003879 |
| *Ptprv* | 40.41575 | 1.604437 | 0.731976 | 13.45035 | 0.003757 |
| *Pla2g4a* | 56.64 | 1.606294 | 0.637105 | 11.23342 | 0.010528 |
| *Gm13889* | 86.77617 | 1.621925 | 0.61032 | 9.970028 | 0.018823 |
| *Stard10* | 1424.472 | 1.632319 | 0.929859 | 8.664278 | 0.034104 |
| *Enpp6* | 47.58177 | 1.640411 | 0.598975 | 10.90422 | 0.012255 |
| *Col6a5* | 39.62691 | 1.662197 | 0.738932 | 9.749417 | 0.020821 |
| *Ptk2b* | 137.5123 | 1.668283 | 0.506638 | 18.16986 | 0.000406 |
| *Serpina3n* | 1482.44 | 1.669532 | 0.281848 | 47.5488 | 2.66E-10 |
| *Mmp3* | 70.51098 | 1.675106 | 0.632725 | 11.76901 | 0.008218 |
| *AK047676* | 14.70533 | 1.679029 | 1.212395 | 8.747406 | 0.032845 |
| *Camkk1* | 49.97824 | 1.68731 | 0.571496 | 9.03129 | 0.028878 |
| *Kdelr3* | 235.8722 | 1.715813 | 0.376842 | 23.96068 | 2.55E-05 |
| *Mgp* | 2253.393 | 1.725245 | 0.66234 | 8.989642 | 0.029429 |
| *Pycr1* | 54.54985 | 1.726057 | 0.565179 | 9.767059 | 0.020654 |
| *Tmem119* | 130.8065 | 1.736612 | 0.453146 | 23.20408 | 3.66E-05 |
| *Rpp25* | 67.61268 | 1.737701 | 0.554114 | 14.77129 | 0.002023 |
| *Rxfp1* | 24.68714 | 1.744574 | 0.897468 | 14.57258 | 0.002221 |
| *Cgref1* | 27.68124 | 1.751893 | 0.806424 | 8.306481 | 0.040085 |
| *Panx1* | 54.24734 | 1.756592 | 0.668644 | 9.228418 | 0.026403 |
| *Clec11a* | 61.7555 | 1.764177 | 0.671796 | 13.39918 | 0.003848 |
| *Inhba* | 80.21806 | 1.765711 | 0.565805 | 11.81179 | 0.008056 |
| *Tmem108* | 77.13779 | 1.773926 | 0.608705 | 11.63461 | 0.008746 |
| *Egr1* | 238.473 | 1.798489 | 0.37558 | 24.24521 | 2.22E-05 |
| *Gdf15* | 88.29391 | 1.806173 | 0.519513 | 16.63248 | 0.000841 |
| *Ctgf* | 3740.474 | 1.812621 | 0.259777 | 58.1976 | 1.43E-12 |
| *Pzp* | 27.09141 | 1.831774 | 0.751924 | 10.71292 | 0.013384 |
| *Sbk2* | 253.6052 | 1.863561 | 0.891726 | 11.19011 | 0.010741 |
| *Kctd11* | 102.0827 | 1.867948 | 0.497816 | 15.99816 | 0.001135 |
| *Slc7a7* | 124.9057 | 1.869968 | 0.470556 | 21.8943 | 6.86E-05 |
| *Serpinb1a* | 54.92325 | 1.871664 | 0.624429 | 12.02824 | 0.007287 |
| *Trim67* | 92.85553 | 1.887504 | 0.563762 | 19.02496 | 0.00027 |
| *Lgals3* | 84.53743 | 1.89572 | 0.531857 | 31.0789 | 8.18E-07 |
| *Lypd1* | 61.50738 | 1.921537 | 0.56798 | 14.1091 | 0.00276 |
| *Tbc1d1* | 377.5539 | 1.921801 | 0.375013 | 39.33505 | 1.47E-08 |
| *Plvap* | 273.4611 | 1.922314 | 0.363433 | 37.02297 | 4.55E-08 |
| *Slc17a7* | 373.9917 | 1.927852 | 0.677043 | 15.01309 | 0.001805 |
| *Gpr39* | 62.45166 | 1.93395 | 0.562121 | 13.55213 | 0.003583 |
| *Efhd2* | 936.6358 | 1.990928 | 0.287481 | 56.63421 | 3.08E-12 |
| *Sypl2* | 25.98705 | 1.995353 | 0.836591 | 8.181075 | 0.042414 |
| *Dok5* | 20.15011 | 1.995623 | 0.851842 | 10.14437 | 0.017378 |
| *Podn* | 653.6885 | 1.99667 | 0.71752 | 12.18077 | 0.006789 |
| *Ccr2* | 34.43761 | 1.997906 | 0.716871 | 13.82022 | 0.00316 |
| *AK145379* | 378.2131 | 2.00086 | 0.369256 | 53.5053 | 1.43E-11 |
| *H19* | 378.2131 | 2.00086 | 0.369256 | 53.5053 | 1.43E-11 |
| *Serpina3m* | 61.50152 | 2.012815 | 0.522154 | 17.30572 | 0.000611 |
| *Fbln7* | 43.69776 | 2.032275 | 0.782973 | 15.46144 | 0.001462 |
| *Slc17a9* | 28.45813 | 2.04626 | 0.778048 | 10.0439 | 0.018197 |
| *Tmem163* | 140.5699 | 2.047465 | 0.494095 | 34.89034 | 1.29E-07 |
| *Gpnmb* | 141.1576 | 2.05129 | 0.489459 | 61.36087 | 3.01E-13 |
| *Sln* | 1184.208 | 2.056373 | 2.063367 | 8.459719 | 0.037407 |
| *Wisp1* | 110.6569 | 2.067516 | 0.437734 | 21.72553 | 7.44E-05 |
| *Ccl2* | 22.22994 | 2.074295 | 0.952102 | 7.827475 | 0.049715 |
| *Fam38b* | 29.51043 | 2.093317 | 0.853808 | 12.08438 | 0.0071 |
| *Socs3* | 115.5178 | 2.104456 | 0.476847 | 23.47962 | 3.21E-05 |
| *Npr3* | 278.6448 | 2.111761 | 0.357681 | 39.79199 | 1.18E-08 |
| *Smoc1* | 147.9435 | 2.114054 | 0.499447 | 30.70374 | 9.81E-07 |
| *Mt3* | 14.60035 | 2.130658 | 1.059602 | 8.548501 | 0.035937 |
| *Ltbp2* | 439.5086 | 2.137539 | 0.904771 | 9.730908 | 0.020998 |
| *Gm1078* | 675.7621 | 2.146669 | 1.150806 | 10.82564 | 0.012707 |
| *Myl9* | 2775.452 | 2.149917 | 0.681237 | 14.68423 | 0.002107 |
| *Cntfr* | 114.5458 | 2.155177 | 0.56609 | 24.52459 | 1.94E-05 |
| *Aif1l* | 71.29332 | 2.157639 | 0.591436 | 18.32419 | 0.000377 |
| *Fos* | 56.39828 | 2.164438 | 0.743261 | 12.77203 | 0.005156 |
| *Cthrc1* | 11.77878 | 2.18934 | 1.210234 | 9.364538 | 0.024817 |
| *Rab27b* | 29.18525 | 2.189875 | 0.837604 | 8.12458 | 0.043506 |
| *AK046026* | 19.23162 | 2.244584 | 1.027813 | 10.83233 | 0.012668 |
| *Rab15* | 36.72734 | 2.248653 | 0.723735 | 16.01376 | 0.001127 |
| *Shisa3* | 52.68215 | 2.250345 | 0.687232 | 14.86692 | 0.001934 |
| *Srpx* | 142.7736 | 2.271167 | 0.407662 | 33.69875 | 2.29E-07 |
| *Camk1g* | 20.65973 | 2.284921 | 0.882736 | 13.33306 | 0.003969 |
| *Cdh4* | 30.42057 | 2.298148 | 0.748208 | 17.0793 | 0.000681 |
| *Ankrd1* | 30518.27 | 2.307292 | 0.808172 | 8.923519 | 0.030325 |
| *Sds* | 51.48464 | 2.30868 | 0.775184 | 16.46424 | 0.000911 |
| *Slc12a8* | 30.25075 | 2.312068 | 0.804082 | 8.986131 | 0.029476 |
| *Sost* | 32.99952 | 2.339365 | 1.017357 | 16.5217 | 0.000886 |
| *Fgf12* | 111.6445 | 2.367493 | 1.735201 | 11.16703 | 0.010856 |
| *Ildr2* | 45.04657 | 2.373989 | 0.791575 | 19.22565 | 0.000246 |
| *A930001C03Rik* | 54.89713 | 2.391516 | 0.711693 | 11.32258 | 0.010104 |
| *Mybphl* | 993.1268 | 2.419401 | 2.147086 | 9.507333 | 0.023253 |
| *AK045483* | 22.66518 | 2.420754 | 0.946332 | 10.75305 | 0.013139 |
| *Adcy1* | 184.8876 | 2.448937 | 0.450125 | 54.60329 | 8.34E-12 |
| *Nppb* | 1941.915 | 2.483498 | 0.861653 | 9.790036 | 0.020438 |
| *Vsig4* | 28.51561 | 2.507852 | 0.920073 | 14.49845 | 0.0023 |
| *Slc39a8* | 23.52826 | 2.513384 | 1.013259 | 9.191088 | 0.026855 |
| *Crlf1* | 143.5954 | 2.514503 | 1.052688 | 7.924528 | 0.047598 |
| *Cpne7* | 18.47539 | 2.517817 | 0.997463 | 11.2622 | 0.010389 |
| *Thbs1* | 240.4611 | 2.554797 | 0.810495 | 9.897251 | 0.01946 |
| *Sall1* | 18.74255 | 2.568879 | 0.932597 | 8.602548 | 0.03507 |
| *AK149453* | 34.14414 | 2.574619 | 0.718099 | 25.46404 | 1.23E-05 |
| *Pak3* | 27.7097 | 2.594388 | 0.85821 | 12.02433 | 0.0073 |
| *Krt18* | 44.86134 | 2.614465 | 0.654767 | 18.08144 | 0.000423 |
| *C530008M17Rik* | 87.76778 | 2.649798 | 1.169296 | 8.797733 | 0.032105 |
| *Egr2* | 29.93992 | 2.701273 | 0.851386 | 12.45074 | 0.005988 |
| *AI646023* | 27.90637 | 2.704642 | 0.855592 | 13.15681 | 0.004309 |
| *Dkk3* | 1718.646 | 2.735407 | 1.351296 | 8.241493 | 0.041276 |
| *Hspa1a* | 260.236 | 2.757606 | 1.081513 | 10.58459 | 0.014198 |
| *Ahsg* | 218.9476 | 2.762123 | 0.402078 | 72.92164 | 1.01E-15 |
| *Psca* | 50.16939 | 2.786523 | 0.675728 | 29.91133 | 1.44E-06 |
| *Lypd6* | 42.69337 | 2.813242 | 0.759672 | 20.45301 | 0.000137 |
| *Ptgs2* | 20.62607 | 2.922717 | 0.961921 | 11.16424 | 0.01087 |
| *Upk1b* | 13.92025 | 2.930511 | 1.182655 | 10.47124 | 0.014957 |
| *Grin2c* | 69.45601 | 2.9347 | 0.504937 | 46.74607 | 3.94E-10 |
| *Egr3* | 46.89623 | 2.957592 | 0.752607 | 20.08432 | 0.000163 |
| *Vgll2* | 32.9293 | 2.975162 | 0.81161 | 24.70057 | 1.78E-05 |
| *Msln* | 26.44506 | 3.007866 | 0.901629 | 21.22654 | 9.45E-05 |
| *Ngef* | 30.89143 | 3.071961 | 0.897826 | 14.99289 | 0.001823 |
| *Ch25h* | 21.18161 | 3.155447 | 1.043344 | 9.339705 | 0.025099 |
| *Itgax* | 25.07262 | 3.168875 | 1.035767 | 21.96377 | 6.64E-05 |
| *Ryr3* | 134.0484 | 3.198638 | 0.535133 | 51.07415 | 4.72E-11 |
| *Krt19* | 14.38701 | 3.30238 | 1.079924 | 16.86228 | 0.000754 |
| *Hspa1b* | 303.9995 | 3.317489 | 1.267458 | 10.51066 | 0.014689 |
| *Tnc* | 122.0024 | 3.333301 | 0.543779 | 44.43013 | 1.22E-09 |
| *Serpinb1c* | 29.43169 | 3.449008 | 0.971006 | 13.272 | 0.004084 |
| *Krt7* | 9.653507 | 3.46576 | 1.447876 | 11.06495 | 0.01138 |
| *Has1* | 22.21753 | 3.479739 | 1.065519 | 15.48625 | 0.001445 |
| *1700055N04Rik* | 8.374887 | 3.536391 | 1.597653 | 9.544645 | 0.022861 |
| *Lrp8* | 63.06689 | 3.544327 | 0.648358 | 39.95214 | 1.09E-08 |
| *Unc5a* | 9.721354 | 3.655936 | 1.42588 | 9.624591 | 0.022042 |
| *Nppa* | 47726.72 | 3.696375 | 0.888852 | 19.15356 | 0.000254 |
| *Timp1* | 200.5925 | 3.875216 | 1.373696 | 8.246123 | 0.04119 |
| *Lrrn4* | 10.75995 | 3.891076 | 1.380955 | 12.76447 | 0.005175 |
| *Gadl1* | 46.06778 | 4.073804 | 0.705619 | 50.17532 | 7.33E-11 |
| *Clec4d* | 11.84015 | 4.285333 | 1.282955 | 13.47802 | 0.003709 |
| *Kcnf1* | 9.451901 | 4.467961 | 1.663311 | 10.04344 | 0.0182 |
| *Cldn23* | 16.81608 | 4.486711 | 1.122818 | 16.69163 | 0.000818 |
| *Ereg* | 9.118742 | 4.506527 | 1.555579 | 11.75898 | 0.008256 |
| *Mmp12* | 45.45422 | 5.247824 | 1.584121 | 35.39126 | 1.01E-07 |
| *Spp1* | 272.6506 | 5.795523 | 1.572673 | 17.44104 | 0.000573 |
| *Sprr1a* | 68.47901 | 6.104149 | 1.580638 | 16.252 | 0.001007 |
| *Gm14420* | 90.2332 | 6.623974 | 2.220483 | 8.277776 | 0.040607 |
| *Bmp10* | 247.3243 | 9.893955 | 1.466619 | 64.95679 | 5.12E-14 |

# Supplemental Table 3- Downregulated pathways (p < 0.05) based on transcriptomic data comparing untreated α-MHC^719/+^ and wildtype mice

| ONTOLOGY | ID | Description | pvalue | p.adjust | qvalue | Count |
| --- | --- | --- | --- | --- | --- | --- |
| BP | GO:0006631 | fatty acid metabolic process | 1.43E-27 | 3.98E-24 | 3.22E-24 | 39 |
| BP | GO:0046395 | carboxylic acid catabolic process | 1.24E-25 | 1.50E-22 | 1.21E-22 | 29 |
| BP | GO:0016054 | organic acid catabolic process | 1.61E-25 | 1.50E-22 | 1.21E-22 | 29 |
| BP | GO:0009062 | fatty acid catabolic process | 8.91E-25 | 6.22E-22 | 5.02E-22 | 22 |
| BP | GO:0006091 | generation of precursor metabolites and energy | 7.47E-23 | 4.17E-20 | 3.37E-20 | 35 |
| BP | GO:0006635 | fatty acid beta-oxidation | 9.69E-23 | 4.51E-20 | 3.64E-20 | 19 |
| BP | GO:0072329 | monocarboxylic acid catabolic process | 1.46E-22 | 5.81E-20 | 4.69E-20 | 22 |
| BP | GO:0044282 | small molecule catabolic process | 2.05E-22 | 7.15E-20 | 5.78E-20 | 31 |
| BP | GO:0019395 | fatty acid oxidation | 6.37E-22 | 1.97E-19 | 1.59E-19 | 21 |
| BP | GO:0044242 | cellular lipid catabolic process | 9.66E-22 | 2.70E-19 | 2.18E-19 | 26 |
| BP | GO:0034440 | lipid oxidation | 2.70E-21 | 6.86E-19 | 5.54E-19 | 21 |
| BP | GO:0045333 | cellular respiration | 6.15E-21 | 1.43E-18 | 1.16E-18 | 26 |
| BP | GO:0015980 | energy derivation by oxidation of organic compounds | 1.12E-20 | 2.41E-18 | 1.95E-18 | 29 |
| BP | GO:0016042 | lipid catabolic process | 1.22E-20 | 2.43E-18 | 1.97E-18 | 29 |
| BP | GO:0009060 | aerobic respiration | 4.14E-20 | 7.70E-18 | 6.22E-18 | 23 |
| BP | GO:0006119 | oxidative phosphorylation | 5.94E-19 | 1.04E-16 | 8.37E-17 | 20 |
| BP | GO:0009150 | purine ribonucleotide metabolic process | 6.45E-19 | 1.06E-16 | 8.55E-17 | 30 |
| BP | GO:0030258 | lipid modification | 1.88E-18 | 2.91E-16 | 2.35E-16 | 23 |
| BP | GO:0009259 | ribonucleotide metabolic process | 2.32E-18 | 3.41E-16 | 2.76E-16 | 30 |
| BP | GO:0072521 | purine-containing compound metabolic process | 2.47E-18 | 3.45E-16 | 2.79E-16 | 31 |
| BP | GO:0006163 | purine nucleotide metabolic process | 2.98E-18 | 3.96E-16 | 3.20E-16 | 30 |
| BP | GO:0019693 | ribose phosphate metabolic process | 4.57E-18 | 5.80E-16 | 4.68E-16 | 30 |
| BP | GO:0022900 | electron transport chain | 4.25E-17 | 5.16E-15 | 4.17E-15 | 17 |
| BP | GO:0022904 | respiratory electron transport chain | 1.64E-16 | 1.91E-14 | 1.54E-14 | 16 |
| BP | GO:0006637 | acyl-CoA metabolic process | 2.61E-14 | 2.80E-12 | 2.26E-12 | 14 |
| BP | GO:0035383 | thioester metabolic process | 2.61E-14 | 2.80E-12 | 2.26E-12 | 14 |
| BP | GO:0042775 | mitochondrial ATP synthesis coupled electron transport | 3.30E-14 | 3.41E-12 | 2.75E-12 | 13 |
| BP | GO:0042773 | ATP synthesis coupled electron transport | 4.82E-14 | 4.81E-12 | 3.89E-12 | 13 |
| BP | GO:0019646 | aerobic electron transport chain | 5.48E-14 | 5.27E-12 | 4.26E-12 | 12 |
| BP | GO:0009152 | purine ribonucleotide biosynthetic process | 1.35E-13 | 1.26E-11 | 1.02E-11 | 18 |
| BP | GO:0009165 | nucleotide biosynthetic process | 1.51E-13 | 1.36E-11 | 1.10E-11 | 20 |
| BP | GO:1901293 | nucleoside phosphate biosynthetic process | 2.14E-13 | 1.87E-11 | 1.51E-11 | 20 |
| BP | GO:0009260 | ribonucleotide biosynthetic process | 3.70E-13 | 3.13E-11 | 2.52E-11 | 18 |
| BP | GO:0006164 | purine nucleotide biosynthetic process | 5.51E-13 | 4.53E-11 | 3.65E-11 | 18 |
| BP | GO:0033865 | nucleoside bisphosphate metabolic process | 6.31E-13 | 4.76E-11 | 3.85E-11 | 14 |
| BP | GO:0033875 | ribonucleoside bisphosphate metabolic process | 6.31E-13 | 4.76E-11 | 3.85E-11 | 14 |
| BP | GO:0034032 | purine nucleoside bisphosphate metabolic process | 6.31E-13 | 4.76E-11 | 3.85E-11 | 14 |
| BP | GO:0015986 | proton motive force-driven ATP synthesis | 7.47E-13 | 5.39E-11 | 4.35E-11 | 12 |
| BP | GO:0046390 | ribose phosphate biosynthetic process | 7.53E-13 | 5.39E-11 | 4.35E-11 | 18 |
| BP | GO:0072522 | purine-containing compound biosynthetic process | 1.02E-12 | 7.14E-11 | 5.76E-11 | 18 |
| BP | GO:0009083 | branched-chain amino acid catabolic process | 2.80E-12 | 1.91E-10 | 1.54E-10 | 8 |
| BP | GO:0033539 | fatty acid beta-oxidation using acyl-CoA dehydrogenase | 4.83E-12 | 3.21E-10 | 2.59E-10 | 7 |
| BP | GO:0042776 | proton motive force-driven mitochondrial ATP synthesis | 7.99E-12 | 5.19E-10 | 4.19E-10 | 11 |
| BP | GO:0009081 | branched-chain amino acid metabolic process | 1.59E-11 | 1.01E-09 | 8.14E-10 | 8 |
| BP | GO:0009145 | purine nucleoside triphosphate biosynthetic process | 2.78E-11 | 1.72E-09 | 1.39E-09 | 13 |
| BP | GO:0060047 | heart contraction | 3.52E-11 | 2.13E-09 | 1.72E-09 | 17 |
| BP | GO:0009063 | cellular amino acid catabolic process | 3.61E-11 | 2.15E-09 | 1.73E-09 | 12 |
| BP | GO:0003015 | heart process | 7.52E-11 | 4.37E-09 | 3.53E-09 | 17 |
| BP | GO:0009142 | nucleoside triphosphate biosynthetic process | 1.16E-10 | 6.59E-09 | 5.32E-09 | 13 |
| BP | GO:0006754 | ATP biosynthetic process | 1.18E-10 | 6.59E-09 | 5.32E-09 | 12 |
| BP | GO:1903522 | regulation of blood circulation | 1.74E-10 | 9.50E-09 | 7.67E-09 | 17 |
| BP | GO:0008016 | regulation of heart contraction | 1.92E-10 | 1.03E-08 | 8.34E-09 | 15 |
| BP | GO:0009206 | purine ribonucleoside triphosphate biosynthetic process | 3.79E-10 | 1.99E-08 | 1.61E-08 | 12 |
| BP | GO:0009201 | ribonucleoside triphosphate biosynthetic process | 6.18E-10 | 3.19E-08 | 2.58E-08 | 12 |
| BP | GO:0009144 | purine nucleoside triphosphate metabolic process | 7.95E-10 | 4.04E-08 | 3.26E-08 | 16 |
| BP | GO:0046034 | ATP metabolic process | 1.00E-09 | 4.99E-08 | 4.03E-08 | 15 |
| BP | GO:0009141 | nucleoside triphosphate metabolic process | 2.12E-09 | 1.04E-07 | 8.37E-08 | 16 |
| BP | GO:0009205 | purine ribonucleoside triphosphate metabolic process | 4.75E-09 | 2.29E-07 | 1.85E-07 | 15 |
| BP | GO:0006790 | sulfur compound metabolic process | 5.17E-09 | 2.45E-07 | 1.98E-07 | 17 |
| BP | GO:0009199 | ribonucleoside triphosphate metabolic process | 6.53E-09 | 3.04E-07 | 2.46E-07 | 15 |
| BP | GO:0060048 | cardiac muscle contraction | 1.87E-08 | 8.57E-07 | 6.92E-07 | 11 |
| BP | GO:0006941 | striated muscle contraction | 3.30E-08 | 1.49E-06 | 1.20E-06 | 12 |
| BP | GO:0006942 | regulation of striated muscle contraction | 5.76E-08 | 2.55E-06 | 2.06E-06 | 9 |
| BP | GO:0006936 | muscle contraction | 1.05E-07 | 4.58E-06 | 3.70E-06 | 15 |
| BP | GO:0061337 | cardiac conduction | 1.46E-07 | 6.26E-06 | 5.05E-06 | 8 |
| BP | GO:0003012 | muscle system process | 2.07E-07 | 8.74E-06 | 7.06E-06 | 17 |
| BP | GO:0006099 | tricarboxylic acid cycle | 3.24E-07 | 1.35E-05 | 1.09E-05 | 6 |
| BP | GO:0006084 | acetyl-CoA metabolic process | 4.68E-07 | 1.92E-05 | 1.55E-05 | 6 |
| BP | GO:0002027 | regulation of heart rate | 5.54E-07 | 2.24E-05 | 1.81E-05 | 9 |
| BP | GO:1904062 | regulation of cation transmembrane transport | 6.03E-07 | 2.41E-05 | 1.94E-05 | 16 |
| BP | GO:0010880 | regulation of release of sequestered calcium ion into cytosol by sarcoplasmic reticulum | 1.26E-06 | 4.97E-05 | 4.02E-05 | 5 |
| BP | GO:0006813 | potassium ion transport | 1.35E-06 | 5.24E-05 | 4.23E-05 | 12 |
| BP | GO:0014808 | release of sequestered calcium ion into cytosol by sarcoplasmic reticulum | 1.97E-06 | 7.53E-05 | 6.08E-05 | 5 |
| BP | GO:0086003 | cardiac muscle cell contraction | 2.03E-06 | 7.65E-05 | 6.18E-05 | 7 |
| BP | GO:1903514 | release of sequestered calcium ion into cytosol by endoplasmic reticulum | 2.42E-06 | 9.01E-05 | 7.28E-05 | 5 |
| BP | GO:0055117 | regulation of cardiac muscle contraction | 2.46E-06 | 9.05E-05 | 7.31E-05 | 7 |
| BP | GO:0006520 | cellular amino acid metabolic process | 2.61E-06 | 9.48E-05 | 7.65E-05 | 12 |
| BP | GO:0035384 | thioester biosynthetic process | 2.95E-06 | 0.0001043 | 8.42E-05 | 5 |
| BP | GO:0071616 | acyl-CoA biosynthetic process | 2.95E-06 | 0.0001043 | 8.42E-05 | 5 |
| BP | GO:0010876 | lipid localization | 3.12E-06 | 0.000109 | 8.80E-05 | 16 |
| BP | GO:0006122 | mitochondrial electron transport; ubiquinol to cytochrome c | 3.43E-06 | 0.0001182 | 9.54E-05 | 4 |
| BP | GO:0097553 | calcium ion transmembrane import into cytosol | 4.51E-06 | 0.0001537 | 0.0001241 | 10 |
| BP | GO:0002026 | regulation of the force of heart contraction | 6.04E-06 | 0.0002032 | 0.0001641 | 5 |
| BP | GO:0010889 | regulation of sequestering of triglyceride | 6.46E-06 | 0.0002147 | 0.0001734 | 4 |
| BP | GO:0070296 | sarcoplasmic reticulum calcium ion transport | 7.11E-06 | 0.0002335 | 0.0001886 | 5 |
| BP | GO:1903169 | regulation of calcium ion transmembrane transport | 9.38E-06 | 0.0003045 | 0.0002459 | 10 |
| BP | GO:0010257 | NADH dehydrogenase complex assembly | 9.92E-06 | 0.0003147 | 0.0002541 | 6 |
| BP | GO:0032981 | mitochondrial respiratory chain complex I assembly | 9.92E-06 | 0.0003147 | 0.0002541 | 6 |
| BP | GO:0010959 | regulation of metal ion transport | 1.01E-05 | 0.0003166 | 0.0002557 | 15 |
| BP | GO:0035637 | multicellular organismal signaling | 1.15E-05 | 0.0003562 | 0.0002877 | 9 |
| BP | GO:0006937 | regulation of muscle contraction | 1.39E-05 | 0.000426 | 0.000344 | 9 |
| BP | GO:0019755 | one-carbon compound transport | 1.42E-05 | 0.000426 | 0.000344 | 4 |
| BP | GO:0030730 | sequestering of triglyceride | 1.42E-05 | 0.000426 | 0.000344 | 4 |
| BP | GO:0090257 | regulation of muscle system process | 1.44E-05 | 0.0004277 | 0.0003454 | 11 |
| BP | GO:0033866 | nucleoside bisphosphate biosynthetic process | 1.49E-05 | 0.0004277 | 0.0003454 | 5 |
| BP | GO:0034030 | ribonucleoside bisphosphate biosynthetic process | 1.49E-05 | 0.0004277 | 0.0003454 | 5 |
| BP | GO:0034033 | purine nucleoside bisphosphate biosynthetic process | 1.49E-05 | 0.0004277 | 0.0003454 | 5 |
| BP | GO:0019932 | second-messenger-mediated signaling | 1.50E-05 | 0.0004277 | 0.0003454 | 12 |
| BP | GO:0086004 | regulation of cardiac muscle cell contraction | 1.70E-05 | 0.0004795 | 0.0003872 | 5 |
| BP | GO:0070252 | actin-mediated cell contraction | 1.97E-05 | 0.0005508 | 0.0004448 | 7 |
| BP | GO:0055074 | calcium ion homeostasis | 2.40E-05 | 0.0006622 | 0.0005348 | 12 |
| BP | GO:0008217 | regulation of blood pressure | 2.58E-05 | 0.0007072 | 0.0005711 | 10 |
| BP | GO:0001976 | nervous system process involved in regulation of systemic arterial blood pressure | 2.72E-05 | 0.0007364 | 0.0005947 | 4 |
| BP | GO:0033674 | positive regulation of kinase activity | 2.74E-05 | 0.0007364 | 0.0005947 | 14 |
| BP | GO:1901568 | fatty acid derivative metabolic process | 3.15E-05 | 0.0008374 | 0.0006763 | 5 |
| BP | GO:1903115 | regulation of actin filament-based movement | 4.39E-05 | 0.001157 | 0.0009344 | 5 |
| BP | GO:0006874 | cellular calcium ion homeostasis | 4.49E-05 | 0.0011716 | 0.0009461 | 11 |
| BP | GO:0051481 | negative regulation of cytosolic calcium ion concentration | 4.73E-05 | 0.0012238 | 0.0009883 | 4 |
| BP | GO:0019722 | calcium-mediated signaling | 4.92E-05 | 0.001261 | 0.0010184 | 9 |
| BP | GO:0046394 | carboxylic acid biosynthetic process | 5.39E-05 | 0.0013687 | 0.0011053 | 11 |
| BP | GO:0006633 | fatty acid biosynthetic process | 5.46E-05 | 0.0013726 | 0.0011085 | 8 |
| BP | GO:0016053 | organic acid biosynthetic process | 5.56E-05 | 0.0013853 | 0.0011187 | 11 |
| BP | GO:1901606 | alpha-amino acid catabolic process | 5.87E-05 | 0.0014506 | 0.0011715 | 6 |
| BP | GO:0032868 | response to insulin | 6.06E-05 | 0.0014704 | 0.0011875 | 10 |
| BP | GO:0098657 | import into cell | 6.06E-05 | 0.0014704 | 0.0011875 | 10 |
| BP | GO:0072507 | divalent inorganic cation homeostasis | 6.45E-05 | 0.0015531 | 0.0012542 | 12 |
| BP | GO:0051279 | regulation of release of sequestered calcium ion into cytosol | 7.24E-05 | 0.0017279 | 0.0013954 | 6 |
| BP | GO:0051209 | release of sequestered calcium ion into cytosol | 7.74E-05 | 0.0018322 | 0.0014796 | 7 |
| BP | GO:0006875 | cellular metal ion homeostasis | 7.90E-05 | 0.0018539 | 0.0014971 | 13 |
| BP | GO:0072330 | monocarboxylic acid biosynthetic process | 8.07E-05 | 0.0018675 | 0.0015081 | 9 |
| BP | GO:0007411 | axon guidance | 8.12E-05 | 0.0018675 | 0.0015081 | 10 |
| BP | GO:0051283 | negative regulation of sequestering of calcium ion | 8.16E-05 | 0.0018675 | 0.0015081 | 7 |
| BP | GO:0043434 | response to peptide hormone | 8.37E-05 | 0.0018885 | 0.0015251 | 12 |
| BP | GO:0097485 | neuron projection guidance | 8.39E-05 | 0.0018885 | 0.0015251 | 10 |
| BP | GO:0046460 | neutral lipid biosynthetic process | 8.76E-05 | 0.0019415 | 0.0015679 | 5 |
| BP | GO:0046463 | acylglycerol biosynthetic process | 8.76E-05 | 0.0019415 | 0.0015679 | 5 |
| BP | GO:0032869 | cellular response to insulin stimulus | 9.00E-05 | 0.0019737 | 0.0015939 | 9 |
| BP | GO:0051282 | regulation of sequestering of calcium ion | 9.05E-05 | 0.0019737 | 0.0015939 | 7 |
| BP | GO:0019229 | regulation of vasoconstriction | 9.45E-05 | 0.0020456 | 0.0016519 | 6 |
| BP | GO:0051208 | sequestering of calcium ion | 0.0001001 | 0.0021483 | 0.0017349 | 7 |
| BP | GO:0043648 | dicarboxylic acid metabolic process | 0.0001008 | 0.0021483 | 0.0017349 | 6 |
| BP | GO:0072503 | cellular divalent inorganic cation homeostasis | 0.0001016 | 0.0021483 | 0.0017349 | 11 |
| BP | GO:0006120 | mitochondrial electron transport; NADH to ubiquinone | 0.0001023 | 0.0021483 | 0.0017349 | 4 |
| BP | GO:0055067 | monovalent inorganic cation homeostasis | 0.0001136 | 0.0023559 | 0.0019025 | 8 |
| BP | GO:0050848 | regulation of calcium-mediated signaling | 0.0001144 | 0.0023559 | 0.0019025 | 6 |
| BP | GO:0001666 | response to hypoxia | 0.0001152 | 0.0023559 | 0.0019025 | 9 |
| BP | GO:0030048 | actin filament-based movement | 0.0001162 | 0.0023559 | 0.0019025 | 7 |
| BP | GO:0070588 | calcium ion transmembrane transport | 0.0001164 | 0.0023559 | 0.0019025 | 11 |
| BP | GO:0003014 | renal system process | 0.000122 | 0.0024499 | 0.0019785 | 7 |
| BP | GO:0055088 | lipid homeostasis | 0.0001441 | 0.0028739 | 0.0023209 | 8 |
| BP | GO:0071375 | cellular response to peptide hormone stimulus | 0.0001451 | 0.0028739 | 0.0023209 | 10 |
| BP | GO:0033108 | mitochondrial respiratory chain complex assembly | 0.0001547 | 0.0030415 | 0.0024562 | 6 |
| BP | GO:0009437 | carnitine metabolic process | 0.0001629 | 0.0031803 | 0.0025683 | 3 |
| BP | GO:0051235 | maintenance of location | 0.0001769 | 0.0034306 | 0.0027704 | 11 |
| BP | GO:0000038 | very long-chain fatty acid metabolic process | 0.0001933 | 0.003718 | 0.0030025 | 4 |
| BP | GO:0042632 | cholesterol homeostasis | 0.0001944 | 0.003718 | 0.0030025 | 6 |
| BP | GO:0055092 | sterol homeostasis | 0.0002055 | 0.0039033 | 0.0031522 | 6 |
| BP | GO:1905952 | regulation of lipid localization | 0.0002099 | 0.0039591 | 0.0031972 | 8 |
| BP | GO:0032412 | regulation of ion transmembrane transporter activity | 0.0002217 | 0.0041542 | 0.0033548 | 10 |
| BP | GO:0051924 | regulation of calcium ion transport | 0.0002405 | 0.0044765 | 0.0036151 | 10 |
| BP | GO:0006577 | amino-acid betaine metabolic process | 0.0002559 | 0.0047004 | 0.0037959 | 3 |
| BP | GO:0031000 | response to caffeine | 0.0002559 | 0.0047004 | 0.0037959 | 3 |
| BP | GO:0006869 | lipid transport | 0.0002712 | 0.0049487 | 0.0039965 | 12 |
| BP | GO:0036293 | response to decreased oxygen levels | 0.0002743 | 0.0049725 | 0.0040157 | 9 |
| BP | GO:0044262 | cellular carbohydrate metabolic process | 0.0002821 | 0.005082 | 0.004104 | 10 |
| BP | GO:0046496 | nicotinamide nucleotide metabolic process | 0.000287 | 0.0051366 | 0.0041481 | 5 |
| BP | GO:0022898 | regulation of transmembrane transporter activity | 0.0002896 | 0.0051505 | 0.0041594 | 10 |
| BP | GO:0070482 | response to oxygen levels | 0.0003051 | 0.0053915 | 0.004354 | 10 |
| BP | GO:0098739 | import across plasma membrane | 0.0003088 | 0.0054221 | 0.0043788 | 8 |
| BP | GO:0035970 | peptidyl-threonine dephosphorylation | 0.000313 | 0.0054614 | 0.0044105 | 3 |
| BP | GO:0019362 | pyridine nucleotide metabolic process | 0.0003287 | 0.0056996 | 0.0046029 | 5 |
| BP | GO:0086091 | regulation of heart rate by cardiac conduction | 0.0003316 | 0.0057144 | 0.0046148 | 4 |
| BP | GO:1901652 | response to peptide | 0.0003425 | 0.0058667 | 0.0047378 | 12 |
| BP | GO:0042310 | vasoconstriction | 0.0003461 | 0.0058928 | 0.0047589 | 6 |
| BP | GO:0046503 | glycerolipid catabolic process | 0.0003511 | 0.0059417 | 0.0047984 | 5 |
| BP | GO:0014074 | response to purine-containing compound | 0.0003635 | 0.006082 | 0.0049116 | 6 |
| BP | GO:0019432 | triglyceride biosynthetic process | 0.000366 | 0.006082 | 0.0049116 | 4 |
| BP | GO:1901020 | negative regulation of calcium ion transmembrane transporter activity | 0.000366 | 0.006082 | 0.0049116 | 4 |
| BP | GO:0030003 | cellular cation homeostasis | 0.0003701 | 0.0061149 | 0.0049382 | 13 |
| BP | GO:0006744 | ubiquinone biosynthetic process | 0.0003777 | 0.0061304 | 0.0049507 | 3 |
| BP | GO:0035337 | fatty-acyl-CoA metabolic process | 0.0003777 | 0.0061304 | 0.0049507 | 3 |
| BP | GO:1901569 | fatty acid derivative catabolic process | 0.0003777 | 0.0061304 | 0.0049507 | 3 |
| BP | GO:0008344 | adult locomotory behavior | 0.0003816 | 0.0061589 | 0.0049737 | 6 |
| BP | GO:0032409 | regulation of transporter activity | 0.0004133 | 0.0066289 | 0.0053533 | 10 |
| BP | GO:0071805 | potassium ion transmembrane transport | 0.0004155 | 0.0066289 | 0.0053533 | 8 |
| BP | GO:1901663 | quinone biosynthetic process | 0.0004504 | 0.0071443 | 0.0057695 | 3 |
| BP | GO:0046486 | glycerolipid metabolic process | 0.0004638 | 0.007316 | 0.0059082 | 11 |
| BP | GO:1901653 | cellular response to peptide | 0.0004669 | 0.0073238 | 0.0059145 | 10 |
| BP | GO:0051926 | negative regulation of calcium ion transport | 0.0004814 | 0.007509 | 0.006064 | 5 |
| BP | GO:0086065 | cell communication involved in cardiac conduction | 0.0004844 | 0.007514 | 0.0060681 | 4 |
| BP | GO:0006816 | calcium ion transport | 0.0005031 | 0.0077613 | 0.0062678 | 12 |
| BP | GO:0046461 | neutral lipid catabolic process | 0.0005293 | 0.0080206 | 0.0064772 | 4 |
| BP | GO:0046464 | acylglycerol catabolic process | 0.0005293 | 0.0080206 | 0.0064772 | 4 |
| BP | GO:0006085 | acetyl-CoA biosynthetic process | 0.0005315 | 0.0080206 | 0.0064772 | 3 |
| BP | GO:0015669 | gas transport | 0.0005315 | 0.0080206 | 0.0064772 | 3 |
| BP | GO:0120254 | olefinic compound metabolic process | 0.0005678 | 0.0085225 | 0.0068825 | 7 |
| BP | GO:0072524 | pyridine-containing compound metabolic process | 0.0005753 | 0.0085894 | 0.0069366 | 5 |
| BP | GO:0006743 | ubiquinone metabolic process | 0.0006213 | 0.0091785 | 0.0074123 | 3 |
| BP | GO:0060314 | regulation of ryanodine-sensitive calcium-release channel activity | 0.0006213 | 0.0091785 | 0.0074123 | 3 |
| BP | GO:0098659 | inorganic cation import across plasma membrane | 0.0006306 | 0.0092175 | 0.0074438 | 6 |
| BP | GO:0099587 | inorganic ion import across plasma membrane | 0.0006306 | 0.0092175 | 0.0074438 | 6 |
| BP | GO:0001676 | long-chain fatty acid metabolic process | 0.0006583 | 0.0095722 | 0.0077303 | 6 |
| BP | GO:0007628 | adult walking behavior | 0.0006818 | 0.009863 | 0.0079651 | 4 |
| BP | GO:1905954 | positive regulation of lipid localization | 0.0006869 | 0.0098856 | 0.0079833 | 6 |
| BP | GO:0035296 | regulation of tube diameter | 0.000725 | 0.010327 | 0.0083398 | 7 |
| BP | GO:0097746 | blood vessel diameter maintenance | 0.000725 | 0.010327 | 0.0083398 | 7 |
| BP | GO:0009306 | protein secretion | 0.0007359 | 0.0104186 | 0.0084137 | 11 |
| BP | GO:1990573 | potassium ion import across plasma membrane | 0.0007389 | 0.0104186 | 0.0084137 | 4 |
| BP | GO:0035150 | regulation of tube size | 0.00075 | 0.0104824 | 0.0084653 | 7 |
| BP | GO:0035592 | establishment of protein localization to extracellular region | 0.0007509 | 0.0104824 | 0.0084653 | 11 |
| BP | GO:0034219 | carbohydrate transmembrane transport | 0.0007786 | 0.0108151 | 0.008734 | 6 |
| BP | GO:0071692 | protein localization to extracellular region | 0.0008463 | 0.0116967 | 0.0094459 | 11 |
| BP | GO:0090659 | walking behavior | 0.000863 | 0.0118689 | 0.009585 | 4 |
| BP | GO:0008038 | neuron recognition | 0.0009302 | 0.0127312 | 0.0102813 | 4 |
| BP | GO:1902600 | proton transmembrane transport | 0.0010403 | 0.014168 | 0.0114416 | 5 |
| BP | GO:1901605 | alpha-amino acid metabolic process | 0.0010729 | 0.014541 | 0.0117429 | 7 |
| BP | GO:0042180 | cellular ketone metabolic process | 0.001119 | 0.0150929 | 0.0121886 | 8 |
| BP | GO:0007409 | axonogenesis | 0.0011303 | 0.0151726 | 0.0122529 | 12 |
| BP | GO:0010883 | regulation of lipid storage | 0.001154 | 0.0154164 | 0.0124499 | 4 |
| BP | GO:0010884 | positive regulation of lipid storage | 0.0012149 | 0.0161519 | 0.0130438 | 3 |
| BP | GO:0031331 | positive regulation of cellular catabolic process | 0.0012395 | 0.0164016 | 0.0132454 | 11 |
| BP | GO:0032410 | negative regulation of transporter activity | 0.0012641 | 0.0166483 | 0.0134447 | 5 |
| BP | GO:0044272 | sulfur compound biosynthetic process | 0.0013252 | 0.0173713 | 0.0140286 | 5 |
| BP | GO:0071496 | cellular response to external stimulus | 0.0013603 | 0.0176407 | 0.0142461 | 9 |
| BP | GO:0002082 | regulation of oxidative phosphorylation | 0.0013648 | 0.0176407 | 0.0142461 | 3 |
| BP | GO:0071880 | adenylate cyclase-activating adrenergic receptor signaling pathway | 0.0013648 | 0.0176407 | 0.0142461 | 3 |
| BP | GO:0001895 | retina homeostasis | 0.0014131 | 0.0180977 | 0.0146151 | 4 |
| BP | GO:1903170 | negative regulation of calcium ion transmembrane transport | 0.0014131 | 0.0180977 | 0.0146151 | 4 |
| BP | GO:0021954 | central nervous system neuron development | 0.001454 | 0.0185368 | 0.0149698 | 5 |
| BP | GO:2001257 | regulation of cation channel activity | 0.0014961 | 0.0189868 | 0.0153332 | 7 |
| BP | GO:0015908 | fatty acid transport | 0.0015217 | 0.0191889 | 0.0154964 | 5 |
| BP | GO:0071549 | cellular response to dexamethasone stimulus | 0.0015258 | 0.0191889 | 0.0154964 | 3 |
| BP | GO:1901019 | regulation of calcium ion transmembrane transporter activity | 0.0015917 | 0.0199287 | 0.0160938 | 5 |
| BP | GO:0086001 | cardiac muscle cell action potential | 0.0016067 | 0.0200266 | 0.0161729 | 4 |
| BP | GO:0006639 | acylglycerol metabolic process | 0.0016546 | 0.0205314 | 0.0165805 | 6 |
| BP | GO:0045822 | negative regulation of heart contraction | 0.0016982 | 0.0208871 | 0.0168678 | 3 |
| BP | GO:0070293 | renal absorption | 0.0016982 | 0.0208871 | 0.0168678 | 3 |
| BP | GO:0001508 | action potential | 0.0017125 | 0.0209701 | 0.0169348 | 6 |
| BP | GO:0006638 | neutral lipid metabolic process | 0.0017719 | 0.0216028 | 0.0174458 | 6 |
| BP | GO:0046471 | phosphatidylglycerol metabolic process | 0.0018823 | 0.0228499 | 0.0184529 | 3 |
| BP | GO:0050873 | brown fat cell differentiation | 0.0019307 | 0.0233351 | 0.0188447 | 4 |
| BP | GO:0071548 | response to dexamethasone | 0.0020785 | 0.0246938 | 0.019942 | 3 |
| BP | GO:0086019 | cell-cell signaling involved in cardiac conduction | 0.0020785 | 0.0246938 | 0.019942 | 3 |
| BP | GO:1900273 | positive regulation of long-term synaptic potentiation | 0.0020785 | 0.0246938 | 0.019942 | 3 |
| BP | GO:1903523 | negative regulation of blood circulation | 0.0020785 | 0.0246938 | 0.019942 | 3 |
| BP | GO:0008643 | carbohydrate transport | 0.0022326 | 0.026413 | 0.0213303 | 6 |
| BP | GO:0086010 | membrane depolarization during action potential | 0.0022868 | 0.0268268 | 0.0216645 | 3 |
| BP | GO:0090208 | positive regulation of triglyceride metabolic process | 0.0022868 | 0.0268268 | 0.0216645 | 3 |
| BP | GO:1904063 | negative regulation of cation transmembrane transport | 0.0023327 | 0.0272502 | 0.0220065 | 5 |
| BP | GO:0006081 | cellular aldehyde metabolic process | 0.0024293 | 0.0280274 | 0.0226341 | 4 |
| BP | GO:0015909 | long-chain fatty acid transport | 0.0024293 | 0.0280274 | 0.0226341 | 4 |
| BP | GO:0045776 | negative regulation of blood pressure | 0.0024293 | 0.0280274 | 0.0226341 | 4 |
| BP | GO:0034764 | positive regulation of transmembrane transport | 0.0024436 | 0.0280759 | 0.0226733 | 8 |
| BP | GO:0045017 | glycerolipid biosynthetic process | 0.0024576 | 0.0281213 | 0.0227099 | 7 |
| BP | GO:0010259 | multicellular organism aging | 0.0025077 | 0.0282315 | 0.0227989 | 3 |
| BP | GO:0019433 | triglyceride catabolic process | 0.0025077 | 0.0282315 | 0.0227989 | 3 |
| BP | GO:0034389 | lipid droplet organization | 0.0025077 | 0.0282315 | 0.0227989 | 3 |
| BP | GO:0071875 | adrenergic receptor signaling pathway | 0.0025077 | 0.0282315 | 0.0227989 | 3 |
| BP | GO:0010634 | positive regulation of epithelial cell migration | 0.0025335 | 0.0284076 | 0.0229412 | 6 |
| BP | GO:0098900 | regulation of action potential | 0.002709 | 0.0302514 | 0.0244302 | 4 |
| BP | GO:0006641 | triglyceride metabolic process | 0.0027317 | 0.0302514 | 0.0244302 | 5 |
| BP | GO:0050996 | positive regulation of lipid catabolic process | 0.0027413 | 0.0302514 | 0.0244302 | 3 |
| BP | GO:2001014 | regulation of skeletal muscle cell differentiation | 0.0027413 | 0.0302514 | 0.0244302 | 3 |
| BP | GO:0001523 | retinoid metabolic process | 0.0028568 | 0.0314025 | 0.0253598 | 4 |
| BP | GO:0010906 | regulation of glucose metabolic process | 0.0029488 | 0.0321603 | 0.0259717 | 5 |
| BP | GO:0043266 | regulation of potassium ion transport | 0.0029488 | 0.0321603 | 0.0259717 | 5 |
| BP | GO:0048013 | ephrin receptor signaling pathway | 0.0029878 | 0.0324594 | 0.0262133 | 3 |
| BP | GO:0021826 | substrate-independent telencephalic tangential migration | 0.0030953 | 0.0327353 | 0.0264361 | 2 |
| BP | GO:0021843 | substrate-independent telencephalic tangential interneuron migration | 0.0030953 | 0.0327353 | 0.0264361 | 2 |
| BP | GO:0072070 | loop of Henle development | 0.0030953 | 0.0327353 | 0.0264361 | 2 |
| BP | GO:1901856 | negative regulation of cellular respiration | 0.0030953 | 0.0327353 | 0.0264361 | 2 |
| BP | GO:1902224 | ketone body metabolic process | 0.0030953 | 0.0327353 | 0.0264361 | 2 |
| BP | GO:1903589 | positive regulation of blood vessel endothelial cell proliferation involved in sprouting angiogenesis | 0.0030953 | 0.0327353 | 0.0264361 | 2 |
| BP | GO:1903596 | regulation of gap junction assembly | 0.0030953 | 0.0327353 | 0.0264361 | 2 |
| BP | GO:0010675 | regulation of cellular carbohydrate metabolic process | 0.003132 | 0.0329985 | 0.0266486 | 6 |
| BP | GO:0016101 | diterpenoid metabolic process | 0.0031688 | 0.0332603 | 0.02686 | 4 |
| BP | GO:0034766 | negative regulation of ion transmembrane transport | 0.0032974 | 0.0344803 | 0.0278453 | 5 |
| BP | GO:0042886 | amide transport | 0.0034001 | 0.035422 | 0.0286058 | 9 |
| BP | GO:1903715 | regulation of aerobic respiration | 0.0035209 | 0.0365436 | 0.0295115 | 3 |
| BP | GO:0060537 | muscle tissue development | 0.0035572 | 0.0367844 | 0.029706 | 11 |
| BP | GO:0014706 | striated muscle tissue development | 0.0036022 | 0.0371118 | 0.0299704 | 8 |
| BP | GO:0002016 | regulation of blood volume by renin-angiotensin | 0.003762 | 0.0381948 | 0.030845 | 2 |
| BP | GO:0021892 | cerebral cortex GABAergic interneuron differentiation | 0.003762 | 0.0381948 | 0.030845 | 2 |
| BP | GO:0048563 | post-embryonic animal organ morphogenesis | 0.003762 | 0.0381948 | 0.030845 | 2 |
| BP | GO:0071415 | cellular response to purine-containing compound | 0.003762 | 0.0381948 | 0.030845 | 2 |
| BP | GO:0090075 | relaxation of muscle | 0.0038077 | 0.0385186 | 0.0311065 | 3 |
| BP | GO:0051651 | maintenance of location in cell | 0.0038369 | 0.0386733 | 0.0312314 | 7 |
| BP | GO:0007204 | positive regulation of cytosolic calcium ion concentration | 0.0040203 | 0.0403769 | 0.0326072 | 7 |
| BP | GO:0045860 | positive regulation of protein kinase activity | 0.0040784 | 0.0408135 | 0.0329597 | 9 |
| BP | GO:0051482 | positive regulation of cytosolic calcium ion concentration involved in phospholipase C-activating G protein-coupled signaling pathway | 0.0041084 | 0.0408818 | 0.033015 | 3 |
| BP | GO:0003018 | vascular process in circulatory system | 0.0041145 | 0.0408818 | 0.033015 | 7 |
| BP | GO:0016239 | positive regulation of macroautophagy | 0.0042424 | 0.0420027 | 0.0339201 | 4 |
| BP | GO:1901890 | positive regulation of cell junction assembly | 0.0043708 | 0.0431207 | 0.034823 | 5 |
| BP | GO:0042832 | defense response to protozoan | 0.0044231 | 0.0432202 | 0.0349034 | 3 |
| BP | GO:1901661 | quinone metabolic process | 0.0044231 | 0.0432202 | 0.0349034 | 3 |
| BP | GO:0006721 | terpenoid metabolic process | 0.0044424 | 0.0432202 | 0.0349034 | 4 |
| BP | GO:0062012 | regulation of small molecule metabolic process | 0.0044551 | 0.0432202 | 0.0349034 | 9 |
| BP | GO:0032000 | positive regulation of fatty acid beta-oxidation | 0.0044892 | 0.0432202 | 0.0349034 | 2 |
| BP | GO:0060073 | micturition | 0.0044892 | 0.0432202 | 0.0349034 | 2 |
| BP | GO:0086013 | membrane repolarization during cardiac muscle cell action potential | 0.0044892 | 0.0432202 | 0.0349034 | 2 |
| BP | GO:0021680 | cerebellar Purkinje cell layer development | 0.0047521 | 0.0454379 | 0.0366943 | 3 |
| BP | GO:0042398 | cellular modified amino acid biosynthetic process | 0.0047521 | 0.0454379 | 0.0366943 | 3 |
| BP | GO:0003073 | regulation of systemic arterial blood pressure | 0.0049916 | 0.047565 | 0.0384121 | 5 |
| BP | GO:0046879 | hormone secretion | 0.0050273 | 0.0477425 | 0.0385554 | 9 |
| BP | GO:0007568 | aging | 0.0050802 | 0.0480814 | 0.0388291 | 4 |
| BP | GO:0043010 | camera-type eye development | 0.0051135 | 0.048233 | 0.0389516 | 9 |
| BP | GO:0042761 | very long-chain fatty acid biosynthetic process | 0.0052758 | 0.049052 | 0.0396129 | 2 |
| BP | GO:0086015 | SA node cell action potential | 0.0052758 | 0.049052 | 0.0396129 | 2 |
| BP | GO:0086018 | SA node cell to atrial cardiac muscle cell signaling | 0.0052758 | 0.049052 | 0.0396129 | 2 |
| BP | GO:0086070 | SA node cell to atrial cardiac muscle cell communication | 0.0052758 | 0.049052 | 0.0396129 | 2 |
| BP | GO:0043279 | response to alkaloid | 0.0053058 | 0.049052 | 0.0396129 | 4 |
| BP | GO:0050709 | negative regulation of protein secretion | 0.0053058 | 0.049052 | 0.0396129 | 4 |
| BP | GO:0043271 | negative regulation of ion transport | 0.0054243 | 0.0499822 | 0.0403641 | 6 |
| CC | GO:1990204 | oxidoreductase complex | 9.43E-22 | 3.04E-19 | 2.26E-19 | 21 |
| CC | GO:0098798 | mitochondrial protein-containing complex | 2.97E-20 | 4.77E-18 | 3.56E-18 | 27 |
| CC | GO:0070469 | respirasome | 4.52E-18 | 4.85E-16 | 3.61E-16 | 17 |
| CC | GO:0005746 | mitochondrial respirasome | 1.88E-17 | 1.51E-15 | 1.13E-15 | 16 |
| CC | GO:0098803 | respiratory chain complex | 2.79E-17 | 1.79E-15 | 1.34E-15 | 16 |
| CC | GO:0098800 | inner mitochondrial membrane protein complex | 3.12E-16 | 1.67E-14 | 1.25E-14 | 18 |
| CC | GO:0005759 | mitochondrial matrix | 9.29E-14 | 4.28E-12 | 3.19E-12 | 21 |
| CC | GO:1902495 | transmembrane transporter complex | 1.20E-12 | 4.82E-11 | 3.59E-11 | 22 |
| CC | GO:1990351 | transporter complex | 3.72E-12 | 1.33E-10 | 9.92E-11 | 22 |
| CC | GO:0030016 | myofibril | 7.60E-09 | 2.45E-07 | 1.82E-07 | 14 |
| CC | GO:0016528 | sarcoplasm | 1.11E-08 | 3.25E-07 | 2.42E-07 | 10 |
| CC | GO:0043292 | contractile fiber | 1.56E-08 | 4.03E-07 | 3.00E-07 | 14 |
| CC | GO:0016529 | sarcoplasmic reticulum | 1.63E-08 | 4.03E-07 | 3.00E-07 | 9 |
| CC | GO:0030017 | sarcomere | 1.34E-07 | 3.08E-06 | 2.30E-06 | 12 |
| CC | GO:0042383 | sarcolemma | 1.83E-07 | 3.66E-06 | 2.73E-06 | 11 |
| CC | GO:0005747 | mitochondrial respiratory chain complex I | 2.05E-07 | 3.66E-06 | 2.73E-06 | 7 |
| CC | GO:0030964 | NADH dehydrogenase complex | 2.05E-07 | 3.66E-06 | 2.73E-06 | 7 |
| CC | GO:0045271 | respiratory chain complex I | 2.05E-07 | 3.66E-06 | 2.73E-06 | 7 |
| CC | GO:0005777 | peroxisome | 5.94E-07 | 9.57E-06 | 7.13E-06 | 10 |
| CC | GO:0042579 | microbody | 5.94E-07 | 9.57E-06 | 7.13E-06 | 10 |
| CC | GO:0070069 | cytochrome complex | 6.33E-07 | 9.71E-06 | 7.23E-06 | 6 |
| CC | GO:0030315 | T-tubule | 2.13E-06 | 3.11E-05 | 2.32E-05 | 7 |
| CC | GO:0005750 | mitochondrial respiratory chain complex III | 2.32E-06 | 3.11E-05 | 2.32E-05 | 4 |
| CC | GO:0045275 | respiratory chain complex III | 2.32E-06 | 3.11E-05 | 2.32E-05 | 4 |
| CC | GO:0031674 | I band | 3.95E-06 | 5.09E-05 | 3.79E-05 | 9 |
| CC | GO:0030018 | Z disc | 1.62E-05 | 0.0002007 | 0.0001496 | 8 |
| CC | GO:0045121 | membrane raft | 3.39E-05 | 0.0004002 | 0.0002983 | 13 |
| CC | GO:0098857 | membrane microdomain | 3.48E-05 | 0.0004002 | 0.0002983 | 13 |
| CC | GO:0034703 | cation channel complex | 0.0001291 | 0.0014332 | 0.0010682 | 9 |
| CC | GO:0045239 | tricarboxylic acid cycle enzyme complex | 0.0003695 | 0.003966 | 0.002956 | 3 |
| CC | GO:0097730 | non-motile cilium | 0.0005431 | 0.0056411 | 0.0042045 | 7 |
| CC | GO:0046658 | anchored component of plasma membrane | 0.0006237 | 0.0062757 | 0.0046775 | 5 |
| CC | GO:0031362 | anchored component of external side of plasma membrane | 0.000927 | 0.0087795 | 0.0065437 | 3 |
| CC | GO:0033017 | sarcoplasmic reticulum membrane | 0.000927 | 0.0087795 | 0.0065437 | 3 |
| CC | GO:0034702 | ion channel complex | 0.0009572 | 0.0088067 | 0.006564 | 9 |
| CC | GO:0034705 | potassium channel complex | 0.0010062 | 0.0089999 | 0.006708 | 5 |
| CC | GO:0045277 | respiratory chain complex IV | 0.0011891 | 0.010348 | 0.0077128 | 3 |
| CC | GO:0031233 | intrinsic component of external side of plasma membrane | 0.0016624 | 0.0140863 | 0.0104991 | 3 |
| CC | GO:0005811 | lipid droplet | 0.0023509 | 0.0194103 | 0.0144673 | 5 |
| CC | GO:0043209 | myelin sheath | 0.0024191 | 0.0194739 | 0.0145147 | 7 |
| CC | GO:0045240 | dihydrolipoyl dehydrogenase complex | 0.0030504 | 0.0239567 | 0.0178559 | 2 |
| CC | GO:0030062 | mitochondrial tricarboxylic acid cycle enzyme complex | 0.0037076 | 0.0277636 | 0.0206934 | 2 |
| CC | GO:0032593 | insulin-responsive compartment | 0.0037076 | 0.0277636 | 0.0206934 | 2 |
| CC | GO:0008076 | voltage-gated potassium channel complex | 0.0039427 | 0.0288532 | 0.0215055 | 4 |
| CC | GO:0098936 | intrinsic component of postsynaptic membrane | 0.0043551 | 0.0300111 | 0.0223686 | 6 |
| CC | GO:0043230 | extracellular organelle | 0.0043805 | 0.0300111 | 0.0223686 | 5 |
| CC | GO:0065010 | extracellular membrane-bounded organelle | 0.0043805 | 0.0300111 | 0.0223686 | 5 |
| CC | GO:0090533 | cation-transporting ATPase complex | 0.0060329 | 0.0404709 | 0.0301647 | 2 |
| CC | GO:0031225 | anchored component of membrane | 0.0068673 | 0.0435102 | 0.03243 | 6 |
| CC | GO:0005778 | peroxisomal membrane | 0.0068914 | 0.0435102 | 0.03243 | 3 |
| CC | GO:0031903 | microbody membrane | 0.0068914 | 0.0435102 | 0.03243 | 3 |
| CC | GO:0030667 | secretory granule membrane | 0.0074481 | 0.0461208 | 0.0343757 | 4 |
| CC | GO:0098533 | ATPase dependent transmembrane transport complex | 0.0078678 | 0.0469152 | 0.0349678 | 2 |
| CC | GO:0098691 | dopaminergic synapse | 0.0078678 | 0.0469152 | 0.0349678 | 2 |
| CC | GO:0098862 | cluster of actin-based cell projections | 0.0080822 | 0.0473178 | 0.0352679 | 6 |
| CC | GO:0019867 | outer membrane | 0.0088421 | 0.0497514 | 0.0370818 | 6 |
| CC | GO:0031968 | organelle outer membrane | 0.0088421 | 0.0497514 | 0.0370818 | 6 |
| CC | GO:0005901 | caveola | 0.0089614 | 0.0497514 | 0.0370818 | 4 |
| MF | GO:0009055 | electron transfer activity | 9.33E-16 | 4.55E-13 | 3.40E-13 | 14 |
| MF | GO:0016627 | oxidoreductase activity; acting on the CH-CH group of donors | 1.53E-14 | 3.73E-12 | 2.78E-12 | 13 |
| MF | GO:0015399 | primary active transmembrane transporter activity | 1.10E-08 | 1.39E-06 | 1.04E-06 | 12 |
| MF | GO:0004300 | enoyl-CoA hydratase activity | 1.14E-08 | 1.39E-06 | 1.04E-06 | 5 |
| MF | GO:0003995 | acyl-CoA dehydrogenase activity | 3.53E-08 | 2.87E-06 | 2.14E-06 | 5 |
| MF | GO:0052890 | oxidoreductase activity; acting on the CH-CH group of donors; with a flavin as acceptor | 3.53E-08 | 2.87E-06 | 2.14E-06 | 5 |
| MF | GO:0015078 | proton transmembrane transporter activity | 5.17E-08 | 3.60E-06 | 2.69E-06 | 10 |
| MF | GO:0051287 | NAD binding | 9.32E-08 | 5.68E-06 | 4.24E-06 | 8 |
| MF | GO:0015453 | oxidoreduction-driven active transmembrane transporter activity | 2.11E-07 | 1.15E-05 | 8.56E-06 | 7 |
| MF | GO:0016836 | hydro-lyase activity | 1.37E-06 | 6.68E-05 | 4.99E-05 | 7 |
| MF | GO:0022853 | active ion transmembrane transporter activity | 2.29E-06 | 0.0001016 | 7.58E-05 | 12 |
| MF | GO:0016835 | carbon-oxygen lyase activity | 6.77E-06 | 0.0002751 | 0.0002053 | 7 |
| MF | GO:0016829 | lyase activity | 9.25E-06 | 0.0003432 | 0.0002561 | 10 |
| MF | GO:0015079 | potassium ion transmembrane transporter activity | 9.85E-06 | 0.0003432 | 0.0002561 | 9 |
| MF | GO:0050660 | flavin adenine dinucleotide binding | 1.09E-05 | 0.0003539 | 0.0002641 | 7 |
| MF | GO:0022804 | active transmembrane transporter activity | 1.28E-05 | 0.0003895 | 0.0002907 | 14 |
| MF | GO:0015267 | channel activity | 2.33E-05 | 0.0005429 | 0.0004052 | 15 |
| MF | GO:0022803 | passive transmembrane transporter activity | 2.33E-05 | 0.0005429 | 0.0004052 | 15 |
| MF | GO:0016903 | oxidoreductase activity; acting on the aldehyde or oxo group of donors | 2.34E-05 | 0.0005429 | 0.0004052 | 6 |
| MF | GO:0051536 | iron-sulfur cluster binding | 2.34E-05 | 0.0005429 | 0.0004052 | 6 |
| MF | GO:0051540 | metal cluster binding | 2.34E-05 | 0.0005429 | 0.0004052 | 6 |
| MF | GO:0022836 | gated channel activity | 3.24E-05 | 0.0007179 | 0.0005358 | 12 |
| MF | GO:0016408 | C-acyltransferase activity | 3.55E-05 | 0.0007537 | 0.0005625 | 4 |
| MF | GO:0005261 | cation channel activity | 3.86E-05 | 0.0007845 | 0.0005855 | 12 |
| MF | GO:0016616 | oxidoreductase activity; acting on the CH-OH group of donors; NAD or NADP as acceptor | 5.94E-05 | 0.0011298 | 0.0008432 | 8 |
| MF | GO:0016863 | intramolecular oxidoreductase activity; transposing C=C bonds | 6.02E-05 | 0.0011298 | 0.0008432 | 4 |
| MF | GO:0000062 | fatty-acyl-CoA binding | 7.07E-05 | 0.001277 | 0.0009531 | 4 |
| MF | GO:1901567 | fatty acid derivative binding | 8.24E-05 | 0.001429 | 0.0010665 | 4 |
| MF | GO:0016614 | oxidoreductase activity; acting on CH-OH group of donors | 8.49E-05 | 0.001429 | 0.0010665 | 8 |
| MF | GO:0005267 | potassium channel activity | 9.16E-05 | 0.0014907 | 0.0011126 | 7 |
| MF | GO:0005221 | intracellular cyclic nucleotide activated cation channel activity | 0.0001006 | 0.001534 | 0.0011449 | 3 |
| MF | GO:0043855 | cyclic nucleotide-gated ion channel activity | 0.0001006 | 0.001534 | 0.0011449 | 3 |
| MF | GO:0016790 | thiolester hydrolase activity | 0.0001047 | 0.0015478 | 0.0011552 | 5 |
| MF | GO:0120227 | acyl-CoA binding | 0.00011 | 0.0015786 | 0.0011781 | 4 |
| MF | GO:0005216 | ion channel activity | 0.0001312 | 0.0018289 | 0.0013649 | 13 |
| MF | GO:0016289 | CoA hydrolase activity | 0.0001437 | 0.0019484 | 0.0014542 | 4 |
| MF | GO:1901681 | sulfur compound binding | 0.0002004 | 0.0026427 | 0.0019723 | 10 |
| MF | GO:0102991 | myristoyl-CoA hydrolase activity | 0.0002704 | 0.0034719 | 0.0025912 | 3 |
| MF | GO:0051539 | 4 iron; 4 sulfur cluster binding | 0.0003559 | 0.0044531 | 0.0033235 | 4 |
| MF | GO:0004129 | cytochrome-c oxidase activity | 0.0004756 | 0.0056612 | 0.0042251 | 3 |
| MF | GO:0016290 | palmitoyl-CoA hydrolase activity | 0.0004756 | 0.0056612 | 0.0042251 | 3 |
| MF | GO:0016675 | oxidoreductase activity; acting on a heme group of donors | 0.0005612 | 0.0063691 | 0.0047535 | 3 |
| MF | GO:0048038 | quinone binding | 0.0005612 | 0.0063691 | 0.0047535 | 3 |
| MF | GO:0052689 | carboxylic ester hydrolase activity | 0.0007045 | 0.0078139 | 0.0058318 | 7 |
| MF | GO:0019829 | ATPase-coupled cation transmembrane transporter activity | 0.0010727 | 0.0115251 | 0.0086016 | 4 |
| MF | GO:0042578 | phosphoric ester hydrolase activity | 0.0010864 | 0.0115251 | 0.0086016 | 10 |
| MF | GO:0004722 | protein serine/threonine phosphatase activity | 0.0011876 | 0.0123305 | 0.0092027 | 5 |
| MF | GO:0046873 | metal ion transmembrane transporter activity | 0.0012156 | 0.0123587 | 0.0092237 | 11 |
| MF | GO:0016620 | oxidoreductase activity; acting on the aldehyde or oxo group of donors; NAD or NADP as acceptor | 0.0013243 | 0.0131892 | 0.0098435 | 4 |
| MF | GO:0051537 | 2 iron; 2 sulfur cluster binding | 0.00144 | 0.0140542 | 0.0104891 | 3 |
| MF | GO:0016746 | acyltransferase activity | 0.0017539 | 0.0167825 | 0.0125253 | 8 |
| MF | GO:0005217 | intracellular ligand-gated ion channel activity | 0.0017914 | 0.0168115 | 0.0125469 | 3 |
| MF | GO:0016684 | oxidoreductase activity; acting on peroxide as acceptor | 0.0019461 | 0.0173013 | 0.0129125 | 4 |
| MF | GO:0016860 | intramolecular oxidoreductase activity | 0.0019461 | 0.0173013 | 0.0129125 | 4 |
| MF | GO:0016878 | acid-thiol ligase activity | 0.0019854 | 0.0173013 | 0.0129125 | 3 |
| MF | GO:0047617 | acyl-CoA hydrolase activity | 0.0019854 | 0.0173013 | 0.0129125 | 3 |
| MF | GO:0033218 | amide binding | 0.0020846 | 0.0178475 | 0.0133201 | 11 |
| MF | GO:0015662 | P-type ion transporter activity | 0.002192 | 0.0181303 | 0.0135313 | 3 |
| MF | GO:0140358 | P-type transmembrane transporter activity | 0.002192 | 0.0181303 | 0.0135313 | 3 |
| MF | GO:0042626 | ATPase-coupled transmembrane transporter activity | 0.0022365 | 0.0181902 | 0.013576 | 5 |
| MF | GO:0016791 | phosphatase activity | 0.0023356 | 0.0186852 | 0.0139454 | 8 |
| MF | GO:0000287 | magnesium ion binding | 0.0026559 | 0.0209043 | 0.0156015 | 7 |
| MF | GO:0016874 | ligase activity | 0.0029547 | 0.0228875 | 0.0170817 | 6 |
| MF | GO:0005391 | P-type sodium:potassium-exchanging transporter activity | 0.0032112 | 0.0241085 | 0.0179929 | 2 |
| MF | GO:0008554 | P-type sodium transporter activity | 0.0032112 | 0.0241085 | 0.0179929 | 2 |
| MF | GO:0016747 | acyltransferase activity; transferring groups other than amino-acyl groups | 0.0035934 | 0.0264496 | 0.0197402 | 7 |
| MF | GO:0004721 | phosphoprotein phosphatase activity | 0.0037443 | 0.0264496 | 0.0197402 | 6 |
| MF | GO:0016853 | isomerase activity | 0.0037443 | 0.0264496 | 0.0197402 | 6 |
| MF | GO:0008556 | P-type potassium transmembrane transporter activity | 0.0039024 | 0.0264496 | 0.0197402 | 2 |
| MF | GO:0015250 | water channel activity | 0.0039024 | 0.0264496 | 0.0197402 | 2 |
| MF | GO:0048495 | Roundabout binding | 0.0039024 | 0.0264496 | 0.0197402 | 2 |
| MF | GO:1901612 | cardiolipin binding | 0.0039024 | 0.0264496 | 0.0197402 | 2 |
| MF | GO:0016651 | oxidoreductase activity; acting on NAD(P)H | 0.0041254 | 0.0275783 | 0.0205826 | 4 |
| MF | GO:0015144 | carbohydrate transmembrane transporter activity | 0.0043288 | 0.0285464 | 0.0213052 | 3 |
| MF | GO:0016298 | lipase activity | 0.0044162 | 0.0287344 | 0.0214455 | 5 |
| MF | GO:0030955 | potassium ion binding | 0.0046562 | 0.0298979 | 0.0223138 | 2 |
| MF | GO:0016877 | ligase activity; forming carbon-sulfur bonds | 0.0050058 | 0.0317252 | 0.0236776 | 3 |
| MF | GO:0086008 | voltage-gated potassium channel activity involved in cardiac muscle cell action potential repolarization | 0.0054715 | 0.0342321 | 0.0255486 | 2 |
| MF | GO:0005005 | transmembrane-ephrin receptor activity | 0.0063472 | 0.0373183 | 0.0278519 | 2 |
| MF | GO:0005372 | water transmembrane transporter activity | 0.0063472 | 0.0373183 | 0.0278519 | 2 |
| MF | GO:0047372 | acylglycerol lipase activity | 0.0063472 | 0.0373183 | 0.0278519 | 2 |
| MF | GO:0052650 | NADP-retinol dehydrogenase activity | 0.0063472 | 0.0373183 | 0.0278519 | 2 |
| MF | GO:1901611 | phosphatidylglycerol binding | 0.0063472 | 0.0373183 | 0.0278519 | 2 |
| MF | GO:0098631 | cell adhesion mediator activity | 0.0069654 | 0.0404659 | 0.030201 | 3 |
| MF | GO:0004089 | carbonate dehydratase activity | 0.0072821 | 0.0417414 | 0.031153 | 2 |
| MF | GO:0022834 | ligand-gated channel activity | 0.0073561 | 0.0417414 | 0.031153 | 5 |
| MF | GO:0005244 | voltage-gated ion channel activity | 0.0075813 | 0.0425253 | 0.031738 | 6 |
| MF | GO:0022832 | voltage-gated channel activity | 0.0077632 | 0.0430505 | 0.03213 | 6 |
| MF | GO:0005003 | ephrin receptor activity | 0.0082751 | 0.0448696 | 0.0334877 | 2 |
| MF | GO:0070403 | NAD+ binding | 0.0082751 | 0.0448696 | 0.0334877 | 2 |

P-values were adjusted using the Benjamini–Hochberg method; q-values indicate the false discovery rate (FDR).

# Supplemental Table 4- Upregulated pathways (p < 0.05.) based on transcriptomic data comparing untreated α-MHC^719/+^ and wildtype mice

| ONTOLOGY | ID | Description | pvalue | p.adjust | Count |
| --- | --- | --- | --- | --- | --- |
| BP | GO:0030198 | extracellular matrix organization | 4.18E-25 | 6.98E-22 | 38 |
| BP | GO:0043062 | extracellular structure organization | 4.68E-25 | 6.98E-22 | 38 |
| BP | GO:0045229 | external encapsulating structure organization | 5.24E-25 | 6.98E-22 | 38 |
| BP | GO:0030199 | collagen fibril organization | 4.08E-17 | 4.08E-14 | 16 |
| BP | GO:0031589 | cell-substrate adhesion | 1.87E-15 | 1.49E-12 | 30 |
| BP | GO:0032963 | collagen metabolic process | 2.89E-15 | 1.92E-12 | 19 |
| BP | GO:0042060 | wound healing | 3.56E-15 | 2.03E-12 | 30 |
| BP | GO:0061448 | connective tissue development | 4.28E-15 | 2.14E-12 | 27 |
| BP | GO:0001503 | ossification | 1.13E-14 | 5.03E-12 | 31 |
| BP | GO:0007178 | transmembrane receptor protein serine/threonine kinase signaling pathway | 2.20E-14 | 8.80E-12 | 30 |
| BP | GO:0052547 | regulation of peptidase activity | 2.26E-13 | 8.22E-11 | 30 |
| BP | GO:0048771 | tissue remodeling | 2.96E-13 | 9.87E-11 | 21 |
| BP | GO:0010466 | negative regulation of peptidase activity | 7.06E-13 | 2.17E-10 | 23 |
| BP | GO:0010951 | negative regulation of endopeptidase activity | 6.17E-12 | 1.76E-09 | 19 |
| BP | GO:0045861 | negative regulation of proteolysis | 1.34E-11 | 3.57E-09 | 25 |
| BP | GO:0051346 | negative regulation of hydrolase activity | 1.80E-11 | 4.50E-09 | 25 |
| BP | GO:0060249 | anatomical structure homeostasis | 3.35E-11 | 7.89E-09 | 24 |
| BP | GO:0050900 | leukocyte migration | 5.33E-11 | 1.18E-08 | 25 |
| BP | GO:0051216 | cartilage development | 7.04E-11 | 1.48E-08 | 19 |
| BP | GO:0001558 | regulation of cell growth | 8.35E-11 | 1.67E-08 | 27 |
| BP | GO:0032964 | collagen biosynthetic process | 1.62E-10 | 3.08E-08 | 11 |
| BP | GO:0001894 | tissue homeostasis | 2.05E-10 | 3.73E-08 | 21 |
| BP | GO:0060348 | bone development | 2.29E-10 | 3.99E-08 | 20 |
| BP | GO:0007160 | cell-matrix adhesion | 2.58E-10 | 4.30E-08 | 19 |
| BP | GO:0002685 | regulation of leukocyte migration | 2.78E-10 | 4.44E-08 | 19 |
| BP | GO:0052548 | regulation of endopeptidase activity | 4.63E-10 | 7.12E-08 | 23 |
| BP | GO:0071560 | cellular response to transforming growth factor beta stimulus | 4.92E-10 | 7.28E-08 | 19 |
| BP | GO:1901652 | response to peptide | 5.42E-10 | 7.74E-08 | 25 |
| BP | GO:0071559 | response to transforming growth factor beta | 6.48E-10 | 8.93E-08 | 19 |
| BP | GO:0045765 | regulation of angiogenesis | 7.94E-10 | 1.06E-07 | 21 |
| BP | GO:1901342 | regulation of vasculature development | 1.00E-09 | 1.29E-07 | 21 |
| BP | GO:0010810 | regulation of cell-substrate adhesion | 1.05E-09 | 1.31E-07 | 18 |
| BP | GO:0070371 | ERK1 and ERK2 cascade | 1.43E-09 | 1.74E-07 | 22 |
| BP | GO:2000146 | negative regulation of cell motility | 2.09E-09 | 2.45E-07 | 21 |
| BP | GO:0040013 | negative regulation of locomotion | 2.41E-09 | 2.76E-07 | 22 |
| BP | GO:0042063 | gliogenesis | 2.81E-09 | 3.12E-07 | 22 |
| BP | GO:0090092 | regulation of transmembrane receptor protein serine/threonine kinase signaling pathway | 3.67E-09 | 3.96E-07 | 19 |
| BP | GO:0030336 | negative regulation of cell migration | 5.80E-09 | 6.10E-07 | 20 |
| BP | GO:0007162 | negative regulation of cell adhesion | 6.13E-09 | 6.28E-07 | 20 |
| BP | GO:0060326 | cell chemotaxis | 6.47E-09 | 6.46E-07 | 20 |
| BP | GO:0050878 | regulation of body fluid levels | 6.81E-09 | 6.64E-07 | 22 |
| BP | GO:0090287 | regulation of cellular response to growth factor stimulus | 8.01E-09 | 7.62E-07 | 20 |
| BP | GO:0010976 | positive regulation of neuron projection development | 8.27E-09 | 7.69E-07 | 17 |
| BP | GO:0007179 | transforming growth factor beta receptor signaling pathway | 1.03E-08 | 9.35E-07 | 16 |
| BP | GO:0007596 | blood coagulation | 1.20E-08 | 1.06E-06 | 15 |
| BP | GO:0070372 | regulation of ERK1 and ERK2 cascade | 1.22E-08 | 1.06E-06 | 20 |
| BP | GO:0007599 | hemostasis | 1.51E-08 | 1.25E-06 | 15 |
| BP | GO:0050817 | coagulation | 1.51E-08 | 1.25E-06 | 15 |
| BP | GO:1901214 | regulation of neuron death | 1.56E-08 | 1.28E-06 | 22 |
| BP | GO:0030282 | bone mineralization | 1.73E-08 | 1.34E-06 | 13 |
| BP | GO:0045766 | positive regulation of angiogenesis | 1.75E-08 | 1.34E-06 | 15 |
| BP | GO:1904018 | positive regulation of vasculature development | 1.75E-08 | 1.34E-06 | 15 |
| BP | GO:0097529 | myeloid leukocyte migration | 2.27E-08 | 1.72E-06 | 17 |
| BP | GO:0002687 | positive regulation of leukocyte migration | 2.46E-08 | 1.82E-06 | 14 |
| BP | GO:0110148 | biomineralization | 2.70E-08 | 1.97E-06 | 15 |
| BP | GO:0042692 | muscle cell differentiation | 2.87E-08 | 2.04E-06 | 23 |
| BP | GO:0010811 | positive regulation of cell-substrate adhesion | 3.23E-08 | 2.27E-06 | 13 |
| BP | GO:0031644 | regulation of nervous system process | 4.11E-08 | 2.83E-06 | 15 |
| BP | GO:0050767 | regulation of neurogenesis | 5.01E-08 | 3.39E-06 | 23 |
| BP | GO:0030595 | leukocyte chemotaxis | 5.66E-08 | 3.77E-06 | 16 |
| BP | GO:1901215 | negative regulation of neuron death | 8.97E-08 | 5.88E-06 | 17 |
| BP | GO:0031346 | positive regulation of cell projection organization | 9.14E-08 | 5.89E-06 | 22 |
| BP | GO:0031214 | biomineral tissue development | 1.01E-07 | 6.41E-06 | 14 |
| BP | GO:0007229 | integrin-mediated signaling pathway | 1.13E-07 | 7.09E-06 | 11 |
| BP | GO:0070997 | neuron death | 1.30E-07 | 7.97E-06 | 22 |
| BP | GO:0010574 | regulation of vascular endothelial growth factor production | 1.48E-07 | 8.94E-06 | 7 |
| BP | GO:0019221 | cytokine-mediated signaling pathway | 1.80E-07 | 1.07E-05 | 21 |
| BP | GO:0060350 | endochondral bone morphogenesis | 2.06E-07 | 1.19E-05 | 9 |
| BP | GO:1905517 | macrophage migration | 2.06E-07 | 1.19E-05 | 9 |
| BP | GO:1905521 | regulation of macrophage migration | 2.09E-07 | 1.19E-05 | 8 |
| BP | GO:0030111 | regulation of Wnt signaling pathway | 2.29E-07 | 1.29E-05 | 18 |
| BP | GO:0008347 | glial cell migration | 3.03E-07 | 1.68E-05 | 9 |
| BP | GO:0070374 | positive regulation of ERK1 and ERK2 cascade | 3.21E-07 | 1.75E-05 | 15 |
| BP | GO:0030178 | negative regulation of Wnt signaling pathway | 3.28E-07 | 1.77E-05 | 13 |
| BP | GO:0010573 | vascular endothelial growth factor production | 3.50E-07 | 1.87E-05 | 7 |
| BP | GO:0002693 | positive regulation of cellular extravasation | 3.81E-07 | 2.00E-05 | 6 |
| BP | GO:0043434 | response to peptide hormone | 4.31E-07 | 2.24E-05 | 19 |
| BP | GO:0030168 | platelet activation | 4.50E-07 | 2.30E-05 | 10 |
| BP | GO:0090101 | negative regulation of transmembrane receptor protein serine/threonine kinase signaling pathway | 5.25E-07 | 2.65E-05 | 12 |
| BP | GO:0016055 | Wnt signaling pathway | 5.56E-07 | 2.78E-05 | 21 |
| BP | GO:0050769 | positive regulation of neurogenesis | 5.90E-07 | 2.87E-05 | 17 |
| BP | GO:1903034 | regulation of response to wounding | 5.96E-07 | 2.87E-05 | 13 |
| BP | GO:0198738 | cell-cell signaling by wnt | 5.97E-07 | 2.87E-05 | 21 |
| BP | GO:0032103 | positive regulation of response to external stimulus | 7.38E-07 | 3.51E-05 | 21 |
| BP | GO:0007015 | actin filament organization | 7.64E-07 | 3.59E-05 | 21 |
| BP | GO:0010720 | positive regulation of cell development | 7.86E-07 | 3.65E-05 | 19 |
| BP | GO:0006953 | acute-phase response | 8.92E-07 | 4.10E-05 | 7 |
| BP | GO:0071674 | mononuclear cell migration | 9.26E-07 | 4.21E-05 | 13 |
| BP | GO:0048660 | regulation of smooth muscle cell proliferation | 9.85E-07 | 4.42E-05 | 13 |
| BP | GO:0071621 | granulocyte chemotaxis | 1.01E-06 | 4.50E-05 | 11 |
| BP | GO:0018149 | peptide cross-linking | 1.06E-06 | 4.65E-05 | 7 |
| BP | GO:0001667 | ameboidal-type cell migration | 1.12E-06 | 4.84E-05 | 21 |
| BP | GO:0046849 | bone remodeling | 1.14E-06 | 4.92E-05 | 10 |
| BP | GO:0048659 | smooth muscle cell proliferation | 1.49E-06 | 6.35E-05 | 13 |
| BP | GO:0097530 | granulocyte migration | 1.58E-06 | 6.63E-05 | 12 |
| BP | GO:0090090 | negative regulation of canonical Wnt signaling pathway | 1.59E-06 | 6.63E-05 | 11 |
| BP | GO:0060349 | bone morphogenesis | 1.62E-06 | 6.66E-05 | 10 |
| BP | GO:0051962 | positive regulation of nervous system development | 1.67E-06 | 6.81E-05 | 18 |
| BP | GO:0050727 | regulation of inflammatory response | 1.74E-06 | 6.94E-05 | 18 |
| BP | GO:0051604 | protein maturation | 1.74E-06 | 6.94E-05 | 18 |
| BP | GO:0045926 | negative regulation of growth | 1.86E-06 | 7.37E-05 | 15 |
| BP | GO:0035966 | response to topologically incorrect protein | 1.98E-06 | 7.75E-05 | 11 |
| BP | GO:0002526 | acute inflammatory response | 2.07E-06 | 8.04E-05 | 10 |
| BP | GO:0060070 | canonical Wnt signaling pathway | 2.24E-06 | 8.62E-05 | 16 |
| BP | GO:0043277 | apoptotic cell clearance | 2.33E-06 | 8.80E-05 | 7 |
| BP | GO:0033002 | muscle cell proliferation | 2.35E-06 | 8.80E-05 | 15 |
| BP | GO:0030308 | negative regulation of cell growth | 2.36E-06 | 8.80E-05 | 13 |
| BP | GO:0003018 | vascular process in circulatory system | 2.70E-06 | 9.91E-05 | 14 |
| BP | GO:0032965 | regulation of collagen biosynthetic process | 2.70E-06 | 9.91E-05 | 7 |
| BP | GO:0010959 | regulation of metal ion transport | 2.75E-06 | 0.0001 | 20 |
| BP | GO:0030324 | lung development | 2.84E-06 | 0.000102 | 14 |
| BP | GO:0044706 | multi-multicellular organism process | 2.93E-06 | 0.000105 | 13 |
| BP | GO:0030323 | respiratory tube development | 3.29E-06 | 0.000116 | 14 |
| BP | GO:0030574 | collagen catabolic process | 3.58E-06 | 0.000126 | 7 |
| BP | GO:0002062 | chondrocyte differentiation | 3.88E-06 | 0.000135 | 10 |
| BP | GO:0003012 | muscle system process | 4.41E-06 | 0.000152 | 19 |
| BP | GO:1990868 | response to chemokine | 4.76E-06 | 0.000161 | 8 |
| BP | GO:1990869 | cellular response to chemokine | 4.76E-06 | 0.000161 | 8 |
| BP | GO:0045862 | positive regulation of proteolysis | 4.98E-06 | 0.000167 | 17 |
| BP | GO:0060828 | regulation of canonical Wnt signaling pathway | 5.28E-06 | 0.000176 | 14 |
| BP | GO:0030850 | prostate gland development | 5.35E-06 | 0.000176 | 7 |
| BP | GO:0002063 | chondrocyte development | 5.37E-06 | 0.000176 | 6 |
| BP | GO:0043588 | skin development | 5.76E-06 | 0.000187 | 16 |
| BP | GO:0048608 | reproductive structure development | 5.99E-06 | 0.000192 | 16 |
| BP | GO:0014812 | muscle cell migration | 6.01E-06 | 0.000192 | 10 |
| BP | GO:0048247 | lymphocyte chemotaxis | 6.08E-06 | 0.000192 | 7 |
| BP | GO:0046697 | decidualization | 6.10E-06 | 0.000192 | 5 |
| BP | GO:0010712 | regulation of collagen metabolic process | 6.89E-06 | 0.000215 | 7 |
| BP | GO:0061458 | reproductive system development | 6.99E-06 | 0.000216 | 16 |
| BP | GO:0060395 | SMAD protein signal transduction | 7.42E-06 | 0.000228 | 9 |
| BP | GO:0044342 | type B pancreatic cell proliferation | 7.53E-06 | 0.00023 | 6 |
| BP | GO:0044703 | multi-organism reproductive process | 7.72E-06 | 0.000234 | 12 |
| BP | GO:0030593 | neutrophil chemotaxis | 8.04E-06 | 0.000241 | 9 |
| BP | GO:0010977 | negative regulation of neuron projection development | 8.28E-06 | 0.000247 | 11 |
| BP | GO:0007159 | leukocyte cell-cell adhesion | 8.33E-06 | 0.000247 | 18 |
| BP | GO:0001649 | osteoblast differentiation | 8.53E-06 | 0.00025 | 13 |
| BP | GO:0045453 | bone resorption | 8.63E-06 | 0.00025 | 8 |
| BP | GO:1903036 | positive regulation of response to wounding | 8.63E-06 | 0.00025 | 8 |
| BP | GO:0051146 | striated muscle cell differentiation | 8.76E-06 | 0.000252 | 16 |
| BP | GO:0048705 | skeletal system morphogenesis | 9.03E-06 | 0.000258 | 14 |
| BP | GO:1990266 | neutrophil migration | 9.72E-06 | 0.000276 | 10 |
| BP | GO:0060541 | respiratory system development | 1.17E-05 | 0.000328 | 14 |
| BP | GO:0071604 | transforming growth factor beta production | 1.20E-05 | 0.000336 | 6 |
| BP | GO:0002683 | negative regulation of immune system process | 1.25E-05 | 0.000346 | 19 |
| BP | GO:0061041 | regulation of wound healing | 1.26E-05 | 0.000348 | 10 |
| BP | GO:0072676 | lymphocyte migration | 1.28E-05 | 0.000349 | 9 |
| BP | GO:0043542 | endothelial cell migration | 1.30E-05 | 0.000353 | 13 |
| BP | GO:0043154 | negative regulation of cysteine-type endopeptidase activity involved in apoptotic process | 1.36E-05 | 0.000368 | 8 |
| BP | GO:0010812 | negative regulation of cell-substrate adhesion | 1.39E-05 | 0.000372 | 7 |
| BP | GO:0030278 | regulation of ossification | 1.53E-05 | 0.000406 | 10 |
| BP | GO:0051017 | actin filament bundle assembly | 1.55E-05 | 0.000411 | 11 |
| BP | GO:0014015 | positive regulation of gliogenesis | 1.62E-05 | 0.000425 | 8 |
| BP | GO:0001655 | urogenital system development | 1.63E-05 | 0.000425 | 17 |
| BP | GO:0070098 | chemokine-mediated signaling pathway | 1.72E-05 | 0.000446 | 7 |
| BP | GO:0061572 | actin filament bundle organization | 1.83E-05 | 0.000471 | 11 |
| BP | GO:0010575 | positive regulation of vascular endothelial growth factor production | 1.87E-05 | 0.000479 | 5 |
| BP | GO:0060393 | regulation of pathway-restricted SMAD protein phosphorylation | 1.91E-05 | 0.000483 | 7 |
| BP | GO:0061077 | chaperone-mediated protein folding | 1.91E-05 | 0.000483 | 7 |
| BP | GO:0042552 | myelination | 1.93E-05 | 0.000484 | 11 |
| BP | GO:0001101 | response to acid chemical | 2.01E-05 | 0.000502 | 12 |
| BP | GO:0030509 | BMP signaling pathway | 2.03E-05 | 0.000504 | 11 |
| BP | GO:0006986 | response to unfolded protein | 2.11E-05 | 0.000519 | 9 |
| BP | GO:0085029 | extracellular matrix assembly | 2.12E-05 | 0.000519 | 6 |
| BP | GO:0007272 | ensheathment of neurons | 2.26E-05 | 0.000546 | 11 |
| BP | GO:0008366 | axon ensheathment | 2.26E-05 | 0.000546 | 11 |
| BP | GO:1905048 | regulation of metallopeptidase activity | 2.51E-05 | 0.000603 | 4 |
| BP | GO:0006816 | calcium ion transport | 2.71E-05 | 0.000648 | 18 |
| BP | GO:0002691 | regulation of cellular extravasation | 2.75E-05 | 0.000654 | 6 |
| BP | GO:0007409 | axonogenesis | 2.79E-05 | 0.00066 | 19 |
| BP | GO:0060389 | pathway-restricted SMAD protein phosphorylation | 2.85E-05 | 0.000668 | 7 |
| BP | GO:0010631 | epithelial cell migration | 2.86E-05 | 0.000668 | 15 |
| BP | GO:0043523 | regulation of neuron apoptotic process | 2.92E-05 | 0.000678 | 14 |
| BP | GO:0090132 | epithelium migration | 3.07E-05 | 0.000708 | 15 |
| BP | GO:0022407 | regulation of cell-cell adhesion | 3.11E-05 | 0.000715 | 19 |
| BP | GO:0043534 | blood vessel endothelial cell migration | 3.15E-05 | 0.000719 | 9 |
| BP | GO:0002263 | cell activation involved in immune response | 3.29E-05 | 0.000742 | 15 |
| BP | GO:0090130 | tissue migration | 3.29E-05 | 0.000742 | 15 |
| BP | GO:1904062 | regulation of cation transmembrane transport | 3.35E-05 | 0.000752 | 17 |
| BP | GO:0006957 | complement activation; alternative pathway | 3.38E-05 | 0.000755 | 4 |
| BP | GO:0150076 | neuroinflammatory response | 3.45E-05 | 0.000765 | 7 |
| BP | GO:0048010 | vascular endothelial growth factor receptor signaling pathway | 3.52E-05 | 0.000772 | 6 |
| BP | GO:0071229 | cellular response to acid chemical | 3.55E-05 | 0.000772 | 11 |
| BP | GO:0071772 | response to BMP | 3.55E-05 | 0.000772 | 11 |
| BP | GO:0071773 | cellular response to BMP stimulus | 3.55E-05 | 0.000772 | 11 |
| BP | GO:2000117 | negative regulation of cysteine-type endopeptidase activity | 3.62E-05 | 0.000783 | 8 |
| BP | GO:0001818 | negative regulation of cytokine production | 3.79E-05 | 0.000814 | 14 |
| BP | GO:0022604 | regulation of cell morphogenesis | 4.03E-05 | 0.000862 | 15 |
| BP | GO:0070613 | regulation of protein processing | 4.14E-05 | 0.00088 | 7 |
| BP | GO:0010752 | regulation of cGMP-mediated signaling | 4.46E-05 | 0.000938 | 4 |
| BP | GO:0090177 | establishment of planar polarity involved in neural tube closure | 4.46E-05 | 0.000938 | 4 |
| BP | GO:0002690 | positive regulation of leukocyte chemotaxis | 4.52E-05 | 0.000946 | 8 |
| BP | GO:0032967 | positive regulation of collagen biosynthetic process | 4.58E-05 | 0.000953 | 5 |
| BP | GO:0042391 | regulation of membrane potential | 4.77E-05 | 0.000987 | 18 |
| BP | GO:0007613 | memory | 4.81E-05 | 0.000992 | 10 |
| BP | GO:0014910 | regulation of smooth muscle cell migration | 4.86E-05 | 0.000996 | 8 |
| BP | GO:0055001 | muscle cell development | 4.92E-05 | 0.000997 | 12 |
| BP | GO:0061138 | morphogenesis of a branching epithelium | 4.92E-05 | 0.000997 | 12 |
| BP | GO:0009749 | response to glucose | 4.97E-05 | 0.001003 | 11 |
| BP | GO:2000377 | regulation of reactive oxygen species metabolic process | 5.08E-05 | 0.001019 | 10 |
| BP | GO:0043524 | negative regulation of neuron apoptotic process | 5.21E-05 | 0.001038 | 11 |
| BP | GO:0031638 | zymogen activation | 5.22E-05 | 0.001038 | 8 |
| BP | GO:0001893 | maternal placenta development | 5.37E-05 | 0.001057 | 5 |
| BP | GO:0035987 | endodermal cell differentiation | 5.37E-05 | 0.001057 | 5 |
| BP | GO:0009746 | response to hexose | 5.71E-05 | 0.001118 | 11 |
| BP | GO:0071498 | cellular response to fluid shear stress | 5.78E-05 | 0.001126 | 4 |
| BP | GO:1903317 | regulation of protein maturation | 6.40E-05 | 0.001231 | 7 |
| BP | GO:0090288 | negative regulation of cellular response to growth factor stimulus | 6.44E-05 | 0.001231 | 8 |
| BP | GO:0034764 | positive regulation of transmembrane transport | 6.44E-05 | 0.001231 | 13 |
| BP | GO:0048588 | developmental cell growth | 6.44E-05 | 0.001231 | 13 |
| BP | GO:0034284 | response to monosaccharide | 6.54E-05 | 0.001245 | 11 |
| BP | GO:0006898 | receptor-mediated endocytosis | 6.68E-05 | 0.001265 | 13 |
| BP | GO:1905475 | regulation of protein localization to membrane | 6.84E-05 | 0.00129 | 11 |
| BP | GO:0048732 | gland development | 6.94E-05 | 0.001302 | 18 |
| BP | GO:0042176 | regulation of protein catabolic process | 7.02E-05 | 0.00131 | 15 |
| BP | GO:0097191 | extrinsic apoptotic signaling pathway | 7.12E-05 | 0.001323 | 12 |
| BP | GO:0010714 | positive regulation of collagen metabolic process | 7.26E-05 | 0.001341 | 5 |
| BP | GO:0010755 | regulation of plasminogen activation | 7.35E-05 | 0.001341 | 4 |
| BP | GO:0042249 | establishment of planar polarity of embryonic epithelium | 7.35E-05 | 0.001341 | 4 |
| BP | GO:0061430 | bone trabecula morphogenesis | 7.35E-05 | 0.001341 | 4 |
| BP | GO:0050678 | regulation of epithelial cell proliferation | 7.51E-05 | 0.001364 | 17 |
| BP | GO:0006457 | protein folding | 7.63E-05 | 0.001374 | 10 |
| BP | GO:0006936 | muscle contraction | 7.63E-05 | 0.001374 | 14 |
| BP | GO:0060560 | developmental growth involved in morphogenesis | 7.73E-05 | 0.001386 | 13 |
| BP | GO:0048638 | regulation of developmental growth | 8.19E-05 | 0.00146 | 16 |
| BP | GO:0003158 | endothelium development | 8.26E-05 | 0.00146 | 9 |
| BP | GO:0017015 | regulation of transforming growth factor beta receptor signaling pathway | 8.26E-05 | 0.00146 | 9 |
| BP | GO:0007565 | female pregnancy | 8.83E-05 | 0.001538 | 10 |
| BP | GO:0045123 | cellular extravasation | 8.85E-05 | 0.001538 | 7 |
| BP | GO:0043491 | protein kinase B signaling | 8.90E-05 | 0.001538 | 11 |
| BP | GO:0016525 | negative regulation of angiogenesis | 8.98E-05 | 0.001538 | 8 |
| BP | GO:0035967 | cellular response to topologically incorrect protein | 8.98E-05 | 0.001538 | 8 |
| BP | GO:0110149 | regulation of biomineralization | 8.98E-05 | 0.001538 | 8 |
| BP | GO:0045860 | positive regulation of protein kinase activity | 8.99E-05 | 0.001538 | 15 |
| BP | GO:1901653 | cellular response to peptide | 9.01E-05 | 0.001538 | 14 |
| BP | GO:0003007 | heart morphogenesis | 9.25E-05 | 0.001569 | 13 |
| BP | GO:0071230 | cellular response to amino acid stimulus | 9.27E-05 | 0.001569 | 10 |
| BP | GO:0072593 | reactive oxygen species metabolic process | 9.38E-05 | 0.001581 | 12 |
| BP | GO:0035567 | non-canonical Wnt signaling pathway | 9.45E-05 | 0.001586 | 6 |
| BP | GO:1903844 | regulation of cellular response to transforming growth factor beta stimulus | 9.74E-05 | 0.001629 | 9 |
| BP | GO:0016485 | protein processing | 9.92E-05 | 0.001652 | 13 |
| BP | GO:0014909 | smooth muscle cell migration | 0.000102 | 0.001684 | 8 |
| BP | GO:2000181 | negative regulation of blood vessel morphogenesis | 0.000102 | 0.001684 | 8 |
| BP | GO:1901343 | negative regulation of vasculature development | 0.000109 | 0.001784 | 8 |
| BP | GO:0001763 | morphogenesis of a branching structure | 0.000109 | 0.001784 | 12 |
| BP | GO:0002366 | leukocyte activation involved in immune response | 0.000109 | 0.001784 | 14 |
| BP | GO:0051402 | neuron apoptotic process | 0.000113 | 0.001834 | 14 |
| BP | GO:0090594 | inflammatory response to wounding | 0.000114 | 0.001845 | 4 |
| BP | GO:0031345 | negative regulation of cell projection organization | 0.000115 | 0.001847 | 11 |
| BP | GO:1903557 | positive regulation of tumor necrosis factor superfamily cytokine production | 0.000116 | 0.001855 | 8 |
| BP | GO:0009743 | response to carbohydrate | 0.000119 | 0.001898 | 11 |
| BP | GO:0006809 | nitric oxide biosynthetic process | 0.00012 | 0.001898 | 7 |
| BP | GO:0034103 | regulation of tissue remodeling | 0.00012 | 0.001898 | 7 |
| BP | GO:0051966 | regulation of synaptic transmission; glutamatergic | 0.00012 | 0.001898 | 7 |
| BP | GO:0048144 | fibroblast proliferation | 0.000121 | 0.001898 | 9 |
| BP | GO:0031032 | actomyosin structure organization | 0.000125 | 0.001951 | 11 |
| BP | GO:0071634 | regulation of transforming growth factor beta production | 0.000126 | 0.00196 | 5 |
| BP | GO:0002548 | monocyte chemotaxis | 0.000126 | 0.001962 | 6 |
| BP | GO:0001890 | placenta development | 0.000129 | 0.001992 | 10 |
| BP | GO:0051047 | positive regulation of secretion | 0.000136 | 0.002091 | 16 |
| BP | GO:0050729 | positive regulation of inflammatory response | 0.000141 | 0.002167 | 9 |
| BP | GO:1904645 | response to amyloid-beta | 0.000142 | 0.002182 | 5 |
| BP | GO:0051924 | regulation of calcium ion transport | 0.000144 | 0.002204 | 13 |
| BP | GO:0034976 | response to endoplasmic reticulum stress | 0.000147 | 0.002221 | 12 |
| BP | GO:0032652 | regulation of interleukin-1 production | 0.000147 | 0.002221 | 8 |
| BP | GO:0035249 | synaptic transmission; glutamatergic | 0.000147 | 0.002221 | 8 |
| BP | GO:0030500 | regulation of bone mineralization | 0.00015 | 0.002247 | 7 |
| BP | GO:0034620 | cellular response to unfolded protein | 0.000161 | 0.002385 | 7 |
| BP | GO:0002931 | response to ischemia | 0.000161 | 0.002385 | 5 |
| BP | GO:0060351 | cartilage development involved in endochondral bone morphogenesis | 0.000161 | 0.002385 | 5 |
| BP | GO:1903573 | negative regulation of response to endoplasmic reticulum stress | 0.000161 | 0.002385 | 5 |
| BP | GO:0050921 | positive regulation of chemotaxis | 0.000164 | 0.00242 | 9 |
| BP | GO:0042698 | ovulation cycle | 0.000166 | 0.002435 | 6 |
| BP | GO:0010001 | glial cell differentiation | 0.00017 | 0.002482 | 12 |
| BP | GO:0033627 | cell adhesion mediated by integrin | 0.000172 | 0.002511 | 7 |
| BP | GO:0071675 | regulation of mononuclear cell migration | 0.000176 | 0.002551 | 8 |
| BP | GO:0014911 | positive regulation of smooth muscle cell migration | 0.000181 | 0.00261 | 6 |
| BP | GO:0006636 | unsaturated fatty acid biosynthetic process | 0.000182 | 0.00261 | 5 |
| BP | GO:0051968 | positive regulation of synaptic transmission; glutamatergic | 0.000182 | 0.00261 | 5 |
| BP | GO:0043200 | response to amino acid | 0.000183 | 0.002623 | 10 |
| BP | GO:0046209 | nitric oxide metabolic process | 0.000184 | 0.002623 | 7 |
| BP | GO:0050766 | positive regulation of phagocytosis | 0.000184 | 0.002623 | 7 |
| BP | GO:0050866 | negative regulation of cell activation | 0.000185 | 0.002625 | 11 |
| BP | GO:0001659 | temperature homeostasis | 0.000191 | 0.002694 | 10 |
| BP | GO:1903555 | regulation of tumor necrosis factor superfamily cytokine production | 0.000191 | 0.002694 | 10 |
| BP | GO:0008217 | regulation of blood pressure | 0.000193 | 0.002699 | 11 |
| BP | GO:0031099 | regeneration | 0.0002 | 0.002791 | 9 |
| BP | GO:1903706 | regulation of hemopoiesis | 0.000202 | 0.002794 | 16 |
| BP | GO:0030728 | ovulation | 0.000202 | 0.002794 | 4 |
| BP | GO:0031643 | positive regulation of myelination | 0.000202 | 0.002794 | 4 |
| BP | GO:0001706 | endoderm formation | 0.000204 | 0.00281 | 5 |
| BP | GO:0051090 | regulation of DNA-binding transcription factor activity | 0.000207 | 0.002837 | 16 |
| BP | GO:0032612 | interleukin-1 production | 0.000208 | 0.002837 | 8 |
| BP | GO:0050764 | regulation of phagocytosis | 0.000208 | 0.002837 | 8 |
| BP | GO:0030512 | negative regulation of transforming growth factor beta receptor signaling pathway | 0.000211 | 0.002861 | 7 |
| BP | GO:2001057 | reactive nitrogen species metabolic process | 0.000211 | 0.002861 | 7 |
| BP | GO:0050730 | regulation of peptidyl-tyrosine phosphorylation | 0.000216 | 0.002921 | 12 |
| BP | GO:0007616 | long-term memory | 0.000228 | 0.003072 | 5 |
| BP | GO:0002040 | sprouting angiogenesis | 0.000232 | 0.003116 | 8 |
| BP | GO:0060562 | epithelial tube morphogenesis | 0.000238 | 0.003181 | 15 |
| BP | GO:0034405 | response to fluid shear stress | 0.00024 | 0.003197 | 4 |
| BP | GO:0051651 | maintenance of location in cell | 0.000241 | 0.003197 | 11 |
| BP | GO:0008360 | regulation of cell shape | 0.000242 | 0.003197 | 9 |
| BP | GO:0002688 | regulation of leukocyte chemotaxis | 0.000245 | 0.003236 | 8 |
| BP | GO:0002274 | myeloid leukocyte activation | 0.000248 | 0.003257 | 12 |
| BP | GO:0071706 | tumor necrosis factor superfamily cytokine production | 0.000257 | 0.003335 | 10 |
| BP | GO:0007044 | cell-substrate junction assembly | 0.000257 | 0.003335 | 7 |
| BP | GO:0032651 | regulation of interleukin-1 beta production | 0.000257 | 0.003335 | 7 |
| BP | GO:0070555 | response to interleukin-1 | 0.000257 | 0.003335 | 7 |
| BP | GO:0045807 | positive regulation of endocytosis | 0.000259 | 0.003349 | 8 |
| BP | GO:2001233 | regulation of apoptotic signaling pathway | 0.000265 | 0.003413 | 15 |
| BP | GO:0030100 | regulation of endocytosis | 0.000279 | 0.003591 | 11 |
| BP | GO:0043567 | regulation of insulin-like growth factor receptor signaling pathway | 0.000283 | 0.003622 | 4 |
| BP | GO:0009306 | protein secretion | 0.000287 | 0.003658 | 15 |
| BP | GO:0035592 | establishment of protein localization to extracellular region | 0.000294 | 0.003743 | 15 |
| BP | GO:0045600 | positive regulation of fat cell differentiation | 0.000296 | 0.003748 | 6 |
| BP | GO:0099601 | regulation of neurotransmitter receptor activity | 0.000296 | 0.003748 | 6 |
| BP | GO:1990138 | neuron projection extension | 0.000302 | 0.003797 | 10 |
| BP | GO:0048193 | Golgi vesicle transport | 0.000302 | 0.003797 | 12 |
| BP | GO:0018108 | peptidyl-tyrosine phosphorylation | 0.000308 | 0.003847 | 13 |
| BP | GO:0072503 | cellular divalent inorganic cation homeostasis | 0.000308 | 0.003847 | 13 |
| BP | GO:0014009 | glial cell proliferation | 0.00032 | 0.003975 | 6 |
| BP | GO:0030193 | regulation of blood coagulation | 0.00032 | 0.003975 | 6 |
| BP | GO:0018212 | peptidyl-tyrosine modification | 0.000327 | 0.00404 | 13 |
| BP | GO:0002246 | wound healing involved in inflammatory response | 0.00033 | 0.00404 | 3 |
| BP | GO:0071492 | cellular response to UV-A | 0.00033 | 0.00404 | 3 |
| BP | GO:2001280 | positive regulation of unsaturated fatty acid biosynthetic process | 0.00033 | 0.00404 | 3 |
| BP | GO:0002523 | leukocyte migration involved in inflammatory response | 0.000331 | 0.004043 | 4 |
| BP | GO:0071692 | protein localization to extracellular region | 0.000343 | 0.004183 | 15 |
| BP | GO:0048678 | response to axon injury | 0.000346 | 0.004198 | 6 |
| BP | GO:0048246 | macrophage chemotaxis | 0.000349 | 0.00421 | 5 |
| BP | GO:0060135 | maternal process involved in female pregnancy | 0.000349 | 0.00421 | 5 |
| BP | GO:0062012 | regulation of small molecule metabolic process | 0.000356 | 0.00428 | 14 |
| BP | GO:0045428 | regulation of nitric oxide biosynthetic process | 0.000373 | 0.004407 | 6 |
| BP | GO:0048662 | negative regulation of smooth muscle cell proliferation | 0.000373 | 0.004407 | 6 |
| BP | GO:1900046 | regulation of hemostasis | 0.000373 | 0.004407 | 6 |
| BP | GO:0032611 | interleukin-1 beta production | 0.000373 | 0.004407 | 7 |
| BP | GO:0060021 | roof of mouth development | 0.000373 | 0.004407 | 7 |
| BP | GO:0150115 | cell-substrate junction organization | 0.000373 | 0.004407 | 7 |
| BP | GO:0061351 | neural precursor cell proliferation | 0.000381 | 0.004479 | 10 |
| BP | GO:0045780 | positive regulation of bone resorption | 0.000384 | 0.004479 | 4 |
| BP | GO:0062009 | secondary palate development | 0.000384 | 0.004479 | 4 |
| BP | GO:0072376 | protein activation cascade | 0.000384 | 0.004479 | 4 |
| BP | GO:0007517 | muscle organ development | 0.000386 | 0.004479 | 14 |
| BP | GO:1903037 | regulation of leukocyte cell-cell adhesion | 0.000386 | 0.004479 | 14 |
| BP | GO:0014013 | regulation of gliogenesis | 0.000391 | 0.004533 | 8 |
| BP | GO:0051153 | regulation of striated muscle cell differentiation | 0.000419 | 0.004845 | 7 |
| BP | GO:0022408 | negative regulation of cell-cell adhesion | 0.000427 | 0.004909 | 10 |
| BP | GO:0007566 | embryo implantation | 0.000431 | 0.004909 | 6 |
| BP | GO:0010507 | negative regulation of autophagy | 0.000431 | 0.004909 | 6 |
| BP | GO:0050818 | regulation of coagulation | 0.000431 | 0.004909 | 6 |
| BP | GO:1905897 | regulation of response to endoplasmic reticulum stress | 0.000431 | 0.004909 | 6 |
| BP | GO:0031392 | regulation of prostaglandin biosynthetic process | 0.000435 | 0.004912 | 3 |
| BP | GO:0048251 | elastic fiber assembly | 0.000435 | 0.004912 | 3 |
| BP | GO:1902947 | regulation of tau-protein kinase activity | 0.000435 | 0.004912 | 3 |
| BP | GO:0031639 | plasminogen activation | 0.000444 | 0.004997 | 4 |
| BP | GO:0035296 | regulation of tube diameter | 0.000449 | 0.005031 | 9 |
| BP | GO:0097746 | blood vessel diameter maintenance | 0.000449 | 0.005031 | 9 |
| BP | GO:0048675 | axon extension | 0.000453 | 0.00506 | 8 |
| BP | GO:0043281 | regulation of cysteine-type endopeptidase activity involved in apoptotic process | 0.00046 | 0.005123 | 10 |
| BP | GO:0050863 | regulation of T cell activation | 0.000464 | 0.005152 | 14 |
| BP | GO:0042088 | T-helper 1 type immune response | 0.000467 | 0.005166 | 5 |
| BP | GO:0035150 | regulation of tube size | 0.000469 | 0.005168 | 9 |
| BP | GO:0001704 | formation of primary germ layer | 0.000471 | 0.005168 | 7 |
| BP | GO:2000060 | positive regulation of ubiquitin-dependent protein catabolic process | 0.000471 | 0.005168 | 7 |
| BP | GO:0006875 | cellular metal ion homeostasis | 0.000487 | 0.005328 | 15 |
| BP | GO:0034767 | positive regulation of ion transmembrane transport | 0.000495 | 0.005395 | 10 |
| BP | GO:0048146 | positive regulation of fibroblast proliferation | 0.000497 | 0.005395 | 6 |
| BP | GO:0080164 | regulation of nitric oxide metabolic process | 0.000497 | 0.005395 | 6 |
| BP | GO:0070167 | regulation of biomineral tissue development | 0.000498 | 0.005395 | 7 |
| BP | GO:0030195 | negative regulation of blood coagulation | 0.000512 | 0.005529 | 5 |
| BP | GO:0032755 | positive regulation of interleukin-6 production | 0.000527 | 0.005675 | 7 |
| BP | GO:0030516 | regulation of axon extension | 0.000557 | 0.005933 | 7 |
| BP | GO:0042310 | vasoconstriction | 0.000557 | 0.005933 | 7 |
| BP | GO:0048661 | positive regulation of smooth muscle cell proliferation | 0.000557 | 0.005933 | 7 |
| BP | GO:0060394 | negative regulation of pathway-restricted SMAD protein phosphorylation | 0.00056 | 0.005933 | 3 |
| BP | GO:0071635 | negative regulation of transforming growth factor beta production | 0.00056 | 0.005933 | 3 |
| BP | GO:1900047 | negative regulation of hemostasis | 0.00056 | 0.005933 | 5 |
| BP | GO:0051235 | maintenance of location | 0.000561 | 0.005933 | 13 |
| BP | GO:0071347 | cellular response to interleukin-1 | 0.00057 | 0.006011 | 6 |
| BP | GO:0014902 | myotube differentiation | 0.000574 | 0.006012 | 8 |
| BP | GO:0001516 | prostaglandin biosynthetic process | 0.000582 | 0.006012 | 4 |
| BP | GO:0006929 | substrate-dependent cell migration | 0.000582 | 0.006012 | 4 |
| BP | GO:0010955 | negative regulation of protein processing | 0.000582 | 0.006012 | 4 |
| BP | GO:0043032 | positive regulation of macrophage activation | 0.000582 | 0.006012 | 4 |
| BP | GO:0046457 | prostanoid biosynthetic process | 0.000582 | 0.006012 | 4 |
| BP | GO:0060343 | trabecula formation | 0.000582 | 0.006012 | 4 |
| BP | GO:1903318 | negative regulation of protein maturation | 0.000582 | 0.006012 | 4 |
| BP | GO:0043270 | positive regulation of ion transport | 0.000593 | 0.006103 | 13 |
| BP | GO:0019216 | regulation of lipid metabolic process | 0.0006 | 0.006162 | 14 |
| BP | GO:0002042 | cell migration involved in sprouting angiogenesis | 0.000612 | 0.006227 | 5 |
| BP | GO:0031103 | axon regeneration | 0.000612 | 0.006227 | 5 |
| BP | GO:0045124 | regulation of bone resorption | 0.000612 | 0.006227 | 5 |
| BP | GO:0050819 | negative regulation of coagulation | 0.000612 | 0.006227 | 5 |
| BP | GO:1902903 | regulation of supramolecular fiber organization | 0.000615 | 0.006238 | 14 |
| BP | GO:0032760 | positive regulation of tumor necrosis factor production | 0.000621 | 0.006282 | 7 |
| BP | GO:0050770 | regulation of axonogenesis | 0.000623 | 0.006286 | 9 |
| BP | GO:0006909 | phagocytosis | 0.000631 | 0.006348 | 14 |
| BP | GO:0043547 | positive regulation of GTPase activity | 0.000651 | 0.006534 | 11 |
| BP | GO:1905477 | positive regulation of protein localization to membrane | 0.000655 | 0.006561 | 7 |
| BP | GO:0099622 | cardiac muscle cell membrane repolarization | 0.000662 | 0.006594 | 4 |
| BP | GO:1905523 | positive regulation of macrophage migration | 0.000662 | 0.006594 | 4 |
| BP | GO:0071356 | cellular response to tumor necrosis factor | 0.000674 | 0.006699 | 9 |
| BP | GO:0002285 | lymphocyte activation involved in immune response | 0.000683 | 0.00677 | 10 |
| BP | GO:0031349 | positive regulation of defense response | 0.000689 | 0.006815 | 12 |
| BP | GO:0033674 | positive regulation of kinase activity | 0.000694 | 0.00685 | 15 |
| BP | GO:0048680 | positive regulation of axon regeneration | 0.000706 | 0.006884 | 3 |
| BP | GO:0060346 | bone trabecula formation | 0.000706 | 0.006884 | 3 |
| BP | GO:0072683 | T cell extravasation | 0.000706 | 0.006884 | 3 |
| BP | GO:0090179 | planar cell polarity pathway involved in neural tube closure | 0.000706 | 0.006884 | 3 |
| BP | GO:2001279 | regulation of unsaturated fatty acid biosynthetic process | 0.000706 | 0.006884 | 3 |
| BP | GO:1904019 | epithelial cell apoptotic process | 0.00072 | 0.007 | 8 |
| BP | GO:1903076 | regulation of protein localization to plasma membrane | 0.000728 | 0.00706 | 7 |
| BP | GO:0045444 | fat cell differentiation | 0.000737 | 0.007135 | 11 |
| BP | GO:0002825 | regulation of T-helper 1 type immune response | 0.000749 | 0.00719 | 4 |
| BP | GO:0003416 | endochondral bone growth | 0.000749 | 0.00719 | 4 |
| BP | GO:0051085 | chaperone cofactor-dependent protein refolding | 0.000749 | 0.00719 | 4 |
| BP | GO:0072659 | protein localization to plasma membrane | 0.00075 | 0.00719 | 12 |
| BP | GO:0032680 | regulation of tumor necrosis factor production | 0.000757 | 0.007234 | 9 |
| BP | GO:0019233 | sensory perception of pain | 0.000786 | 0.007461 | 8 |
| BP | GO:0045598 | regulation of fat cell differentiation | 0.000786 | 0.007461 | 8 |
| BP | GO:0043271 | negative regulation of ion transport | 0.000786 | 0.007461 | 9 |
| BP | GO:0061001 | regulation of dendritic spine morphogenesis | 0.00079 | 0.007461 | 5 |
| BP | GO:0008088 | axo-dendritic transport | 0.00079 | 0.007461 | 6 |
| BP | GO:0060537 | muscle tissue development | 0.000804 | 0.007575 | 16 |
| BP | GO:0021700 | developmental maturation | 0.000814 | 0.007649 | 13 |
| BP | GO:0050808 | synapse organization | 0.000839 | 0.007854 | 16 |
| BP | GO:0002828 | regulation of type 2 immune response | 0.000843 | 0.007854 | 4 |
| BP | GO:0060740 | prostate gland epithelium morphogenesis | 0.000843 | 0.007854 | 4 |
| BP | GO:1904646 | cellular response to amyloid-beta | 0.000843 | 0.007854 | 4 |
| BP | GO:0061383 | trabecula morphogenesis | 0.000857 | 0.007944 | 5 |
| BP | GO:0003231 | cardiac ventricle development | 0.000857 | 0.007944 | 8 |
| BP | GO:0070141 | response to UV-A | 0.000874 | 0.00796 | 3 |
| BP | GO:0070572 | positive regulation of neuron projection regeneration | 0.000874 | 0.00796 | 3 |
| BP | GO:0086014 | atrial cardiac muscle cell action potential | 0.000874 | 0.00796 | 3 |
| BP | GO:0086026 | atrial cardiac muscle cell to AV node cell signaling | 0.000874 | 0.00796 | 3 |
| BP | GO:0086066 | atrial cardiac muscle cell to AV node cell communication | 0.000874 | 0.00796 | 3 |
| BP | GO:0090178 | regulation of establishment of planar polarity involved in neural tube closure | 0.000874 | 0.00796 | 3 |
| BP | GO:0098840 | protein transport along microtubule | 0.000874 | 0.00796 | 3 |
| BP | GO:0099118 | microtubule-based protein transport | 0.000874 | 0.00796 | 3 |
| BP | GO:0050680 | negative regulation of epithelial cell proliferation | 0.00088 | 0.007991 | 9 |
| BP | GO:0051091 | positive regulation of DNA-binding transcription factor activity | 0.000885 | 0.008021 | 11 |
| BP | GO:0060415 | muscle tissue morphogenesis | 0.000894 | 0.008065 | 6 |
| BP | GO:0071333 | cellular response to glucose stimulus | 0.000894 | 0.008065 | 8 |
| BP | GO:0072507 | divalent inorganic cation homeostasis | 0.000901 | 0.008109 | 13 |
| BP | GO:0050803 | regulation of synapse structure or activity | 0.000912 | 0.008145 | 11 |
| BP | GO:0001935 | endothelial cell proliferation | 0.000913 | 0.008145 | 9 |
| BP | GO:0048754 | branching morphogenesis of an epithelial tube | 0.000913 | 0.008145 | 9 |
| BP | GO:1904064 | positive regulation of cation transmembrane transport | 0.000913 | 0.008145 | 9 |
| BP | GO:0034599 | cellular response to oxidative stress | 0.00094 | 0.008362 | 11 |
| BP | GO:0010758 | regulation of macrophage chemotaxis | 0.000946 | 0.008381 | 4 |
| BP | GO:0060512 | prostate gland morphogenesis | 0.000946 | 0.008381 | 4 |
| BP | GO:0006979 | response to oxidative stress | 0.000951 | 0.008392 | 14 |
| BP | GO:0032102 | negative regulation of response to external stimulus | 0.000951 | 0.008392 | 14 |
| BP | GO:0050679 | positive regulation of epithelial cell proliferation | 0.000956 | 0.008413 | 10 |
| BP | GO:0009410 | response to xenobiotic stimulus | 0.000962 | 0.008451 | 12 |
| BP | GO:0071331 | cellular response to hexose stimulus | 0.000973 | 0.008523 | 8 |
| BP | GO:0032640 | tumor necrosis factor production | 0.000983 | 0.008592 | 9 |
| BP | GO:0045446 | endothelial cell differentiation | 0.000985 | 0.008596 | 7 |
| BP | GO:0071622 | regulation of granulocyte chemotaxis | 0.001003 | 0.008711 | 5 |
| BP | GO:0098815 | modulation of excitatory postsynaptic potential | 0.001003 | 0.008711 | 5 |
| BP | GO:0010565 | regulation of cellular ketone metabolic process | 0.001014 | 0.008769 | 8 |
| BP | GO:0071326 | cellular response to monosaccharide stimulus | 0.001014 | 0.008769 | 8 |
| BP | GO:0006090 | pyruvate metabolic process | 0.001034 | 0.008908 | 7 |
| BP | GO:0090100 | positive regulation of transmembrane receptor protein serine/threonine kinase signaling pathway | 0.001034 | 0.008908 | 7 |
| BP | GO:1903532 | positive regulation of secretion by cell | 0.001047 | 0.008998 | 13 |
| BP | GO:0022612 | gland morphogenesis | 0.001056 | 0.009007 | 8 |
| BP | GO:0051147 | regulation of muscle cell differentiation | 0.001056 | 0.009007 | 8 |
| BP | GO:0001958 | endochondral ossification | 0.001057 | 0.009007 | 4 |
| BP | GO:0036075 | replacement ossification | 0.001057 | 0.009007 | 4 |
| BP | GO:0006968 | cellular defense response | 0.001066 | 0.009063 | 3 |
| BP | GO:0090596 | sensory organ morphogenesis | 0.001071 | 0.00909 | 12 |
| BP | GO:0031641 | regulation of myelination | 0.001082 | 0.009145 | 5 |
| BP | GO:0035924 | cellular response to vascular endothelial growth factor stimulus | 0.001082 | 0.009145 | 5 |
| BP | GO:0016050 | vesicle organization | 0.00113 | 0.009523 | 12 |
| BP | GO:0032970 | regulation of actin filament-based process | 0.001171 | 0.009842 | 14 |
| BP | GO:0043405 | regulation of MAP kinase activity | 0.001175 | 0.009842 | 9 |
| BP | GO:0048009 | insulin-like growth factor receptor signaling pathway | 0.001177 | 0.009842 | 4 |
| BP | GO:0097242 | amyloid-beta clearance | 0.001177 | 0.009842 | 4 |
| BP | GO:0002697 | regulation of immune effector process | 0.001181 | 0.009854 | 15 |
| BP | GO:0070588 | calcium ion transmembrane transport | 0.001191 | 0.009872 | 12 |
| BP | GO:0002286 | T cell activation involved in immune response | 0.001193 | 0.009872 | 7 |
| BP | GO:0048145 | regulation of fibroblast proliferation | 0.001193 | 0.009872 | 7 |
| BP | GO:0061387 | regulation of extent of cell growth | 0.001193 | 0.009872 | 7 |
| BP | GO:0006029 | proteoglycan metabolic process | 0.001201 | 0.009894 | 6 |
| BP | GO:0060079 | excitatory postsynaptic potential | 0.001201 | 0.009894 | 6 |
| BP | GO:0003205 | cardiac chamber development | 0.001217 | 0.010007 | 9 |
| BP | GO:2000177 | regulation of neural precursor cell proliferation | 0.00125 | 0.010221 | 7 |
| BP | GO:0006869 | lipid transport | 0.001253 | 0.010221 | 14 |
| BP | GO:0031102 | neuron projection regeneration | 0.001256 | 0.010221 | 5 |
| BP | GO:0048260 | positive regulation of receptor-mediated endocytosis | 0.001256 | 0.010221 | 5 |
| BP | GO:0090303 | positive regulation of wound healing | 0.001256 | 0.010221 | 5 |
| BP | GO:0045732 | positive regulation of protein catabolic process | 0.00126 | 0.010235 | 9 |
| BP | GO:2001234 | negative regulation of apoptotic signaling pathway | 0.001273 | 0.010315 | 10 |
| BP | GO:0019934 | cGMP-mediated signaling | 0.001307 | 0.010485 | 4 |
| BP | GO:0033687 | osteoblast proliferation | 0.001307 | 0.010485 | 4 |
| BP | GO:0051084 | 'de novo' post-translational protein folding | 0.001307 | 0.010485 | 4 |
| BP | GO:0060071 | Wnt signaling pathway; planar cell polarity pathway | 0.001307 | 0.010485 | 4 |
| BP | GO:0010927 | cellular component assembly involved in morphogenesis | 0.001309 | 0.010485 | 7 |
| BP | GO:1903052 | positive regulation of proteolysis involved in protein catabolic process | 0.001309 | 0.010485 | 7 |
| BP | GO:0010876 | lipid localization | 0.00134 | 0.010698 | 15 |
| BP | GO:0031016 | pancreas development | 0.001344 | 0.010698 | 6 |
| BP | GO:0045104 | intermediate filament cytoskeleton organization | 0.001344 | 0.010698 | 6 |
| BP | GO:0046850 | regulation of bone remodeling | 0.00135 | 0.010705 | 5 |
| BP | GO:0034612 | response to tumor necrosis factor | 0.00135 | 0.010705 | 9 |
| BP | GO:2000116 | regulation of cysteine-type endopeptidase activity | 0.001354 | 0.01071 | 10 |
| BP | GO:0071322 | cellular response to carbohydrate stimulus | 0.001396 | 0.01101 | 8 |
| BP | GO:1990778 | protein localization to cell periphery | 0.001399 | 0.01101 | 13 |
| BP | GO:0001654 | eye development | 0.0014 | 0.01101 | 14 |
| BP | GO:0045103 | intermediate filament-based process | 0.00142 | 0.011128 | 6 |
| BP | GO:0048644 | muscle organ morphogenesis | 0.00142 | 0.011128 | 6 |
| BP | GO:0001953 | negative regulation of cell-matrix adhesion | 0.001446 | 0.01126 | 4 |
| BP | GO:0006458 | 'de novo' protein folding | 0.001446 | 0.01126 | 4 |
| BP | GO:0098868 | bone growth | 0.001446 | 0.01126 | 4 |
| BP | GO:0009064 | glutamine family amino acid metabolic process | 0.001449 | 0.011262 | 5 |
| BP | GO:1990845 | adaptive thermogenesis | 0.001451 | 0.011262 | 8 |
| BP | GO:0150063 | visual system development | 0.001495 | 0.011575 | 14 |
| BP | GO:0032793 | positive regulation of CREB transcription factor activity | 0.001524 | 0.011754 | 3 |
| BP | GO:0070486 | leukocyte aggregation | 0.001524 | 0.011754 | 3 |
| BP | GO:0098930 | axonal transport | 0.001553 | 0.011936 | 5 |
| BP | GO:2000401 | regulation of lymphocyte migration | 0.001553 | 0.011936 | 5 |
| BP | GO:0010632 | regulation of epithelial cell migration | 0.001574 | 0.012074 | 10 |
| BP | GO:0044344 | cellular response to fibroblast growth factor stimulus | 0.001583 | 0.012115 | 6 |
| BP | GO:0050654 | chondroitin sulfate proteoglycan metabolic process | 0.001594 | 0.012183 | 4 |
| BP | GO:0006874 | cellular calcium ion homeostasis | 0.001608 | 0.012265 | 11 |
| BP | GO:0048880 | sensory system development | 0.00163 | 0.012404 | 14 |
| BP | GO:0070661 | leukocyte proliferation | 0.001646 | 0.012505 | 13 |
| BP | GO:0051148 | negative regulation of muscle cell differentiation | 0.001663 | 0.012585 | 5 |
| BP | GO:0072678 | T cell migration | 0.001663 | 0.012585 | 5 |
| BP | GO:0032868 | response to insulin | 0.00167 | 0.012617 | 10 |
| BP | GO:0062197 | cellular response to chemical stress | 0.00174 | 0.013116 | 12 |
| BP | GO:1905332 | positive regulation of morphogenesis of an epithelium | 0.001754 | 0.013185 | 4 |
| BP | GO:0071774 | response to fibroblast growth factor | 0.001759 | 0.013185 | 6 |
| BP | GO:0120162 | positive regulation of cold-induced thermogenesis | 0.001759 | 0.013185 | 6 |
| BP | GO:0048008 | platelet-derived growth factor receptor signaling pathway | 0.001778 | 0.013286 | 5 |
| BP | GO:0033559 | unsaturated fatty acid metabolic process | 0.001787 | 0.013286 | 7 |
| BP | GO:2000027 | regulation of animal organ morphogenesis | 0.001787 | 0.013286 | 7 |
| BP | GO:0010038 | response to metal ion | 0.00179 | 0.013286 | 11 |
| BP | GO:0040037 | negative regulation of fibroblast growth factor receptor signaling pathway | 0.001792 | 0.013286 | 3 |
| BP | GO:1902236 | negative regulation of endoplasmic reticulum stress-induced intrinsic apoptotic signaling pathway | 0.001792 | 0.013286 | 3 |
| BP | GO:0051154 | negative regulation of striated muscle cell differentiation | 0.001924 | 0.014209 | 4 |
| BP | GO:1904037 | positive regulation of epithelial cell apoptotic process | 0.001924 | 0.014209 | 4 |
| BP | GO:0071695 | anatomical structure maturation | 0.001936 | 0.014276 | 11 |
| BP | GO:2001243 | negative regulation of intrinsic apoptotic signaling pathway | 0.001949 | 0.014339 | 6 |
| BP | GO:0001678 | cellular glucose homeostasis | 0.001956 | 0.014339 | 8 |
| BP | GO:0006941 | striated muscle contraction | 0.001956 | 0.014339 | 8 |
| BP | GO:0001933 | negative regulation of protein phosphorylation | 0.002011 | 0.014716 | 12 |
| BP | GO:0042246 | tissue regeneration | 0.002026 | 0.014783 | 5 |
| BP | GO:0001936 | regulation of endothelial cell proliferation | 0.002027 | 0.014783 | 8 |
| BP | GO:0046031 | ADP metabolic process | 0.00205 | 0.014922 | 6 |
| BP | GO:0042326 | negative regulation of phosphorylation | 0.002061 | 0.014974 | 13 |
| BP | GO:0002281 | macrophage activation involved in immune response | 0.002088 | 0.015062 | 3 |
| BP | GO:0030502 | negative regulation of bone mineralization | 0.002088 | 0.015062 | 3 |
| BP | GO:0032252 | secretory granule localization | 0.002088 | 0.015062 | 3 |
| BP | GO:0060221 | retinal rod cell differentiation | 0.002088 | 0.015062 | 3 |
| BP | GO:0044262 | cellular carbohydrate metabolic process | 0.002093 | 0.015067 | 11 |
| BP | GO:0090257 | regulation of muscle system process | 0.002103 | 0.0151 | 10 |
| BP | GO:0090175 | regulation of establishment of planar polarity | 0.002105 | 0.0151 | 4 |
| BP | GO:0001889 | liver development | 0.002114 | 0.015115 | 7 |
| BP | GO:0050773 | regulation of dendrite development | 0.002114 | 0.015115 | 7 |
| BP | GO:1903829 | positive regulation of protein localization | 0.002127 | 0.015174 | 15 |
| BP | GO:0099565 | chemical synaptic transmission; postsynaptic | 0.002155 | 0.015299 | 6 |
| BP | GO:0000768 | syncytium formation by plasma membrane fusion | 0.002159 | 0.015299 | 5 |
| BP | GO:0071677 | positive regulation of mononuclear cell migration | 0.002159 | 0.015299 | 5 |
| BP | GO:0140253 | cell-cell fusion | 0.002159 | 0.015299 | 5 |
| BP | GO:0010594 | regulation of endothelial cell migration | 0.002177 | 0.015396 | 8 |
| BP | GO:0060485 | mesenchyme development | 0.002202 | 0.015549 | 11 |
| BP | GO:0009612 | response to mechanical stimulus | 0.002255 | 0.015891 | 8 |
| BP | GO:0050708 | regulation of protein secretion | 0.002259 | 0.015893 | 11 |
| BP | GO:0001938 | positive regulation of endothelial cell proliferation | 0.002264 | 0.015898 | 6 |
| BP | GO:0042092 | type 2 immune response | 0.002297 | 0.01603 | 4 |
| BP | GO:0048873 | homeostasis of number of cells within a tissue | 0.002297 | 0.01603 | 4 |
| BP | GO:1902624 | positive regulation of neutrophil migration | 0.002297 | 0.01603 | 4 |
| BP | GO:0030239 | myofibril assembly | 0.002299 | 0.01603 | 5 |
| BP | GO:0030003 | cellular cation homeostasis | 0.00234 | 0.016282 | 15 |
| BP | GO:0045216 | cell-cell junction organization | 0.002343 | 0.016282 | 9 |
| BP | GO:0030510 | regulation of BMP signaling pathway | 0.002377 | 0.01646 | 6 |
| BP | GO:0042116 | macrophage activation | 0.002377 | 0.01646 | 6 |
| BP | GO:0002698 | negative regulation of immune effector process | 0.002389 | 0.01649 | 7 |
| BP | GO:0061008 | hepaticobiliary system development | 0.002389 | 0.01649 | 7 |
| BP | GO:0010310 | regulation of hydrogen peroxide metabolic process | 0.002413 | 0.016511 | 3 |
| BP | GO:0050650 | chondroitin sulfate proteoglycan biosynthetic process | 0.002413 | 0.016511 | 3 |
| BP | GO:0072378 | blood coagulation; fibrin clot formation | 0.002413 | 0.016511 | 3 |
| BP | GO:0150146 | cell junction disassembly | 0.002413 | 0.016511 | 3 |
| BP | GO:2000353 | positive regulation of endothelial cell apoptotic process | 0.002413 | 0.016511 | 3 |
| BP | GO:0008202 | steroid metabolic process | 0.002425 | 0.016566 | 12 |
| BP | GO:0006949 | syncytium formation | 0.002444 | 0.016612 | 5 |
| BP | GO:0055002 | striated muscle cell development | 0.002444 | 0.016612 | 5 |
| BP | GO:1904705 | regulation of vascular associated smooth muscle cell proliferation | 0.002444 | 0.016612 | 5 |
| BP | GO:0009100 | glycoprotein metabolic process | 0.002482 | 0.016777 | 12 |
| BP | GO:0050807 | regulation of synapse organization | 0.002484 | 0.016777 | 10 |
| BP | GO:0055123 | digestive system development | 0.002487 | 0.016777 | 7 |
| BP | GO:1904375 | regulation of protein localization to cell periphery | 0.002487 | 0.016777 | 7 |
| BP | GO:0009135 | purine nucleoside diphosphate metabolic process | 0.002494 | 0.016777 | 6 |
| BP | GO:0009179 | purine ribonucleoside diphosphate metabolic process | 0.002494 | 0.016777 | 6 |
| BP | GO:0010952 | positive regulation of peptidase activity | 0.002502 | 0.016802 | 8 |
| BP | GO:0010563 | negative regulation of phosphorus metabolic process | 0.002567 | 0.017182 | 14 |
| BP | GO:0045936 | negative regulation of phosphate metabolic process | 0.002567 | 0.017182 | 14 |
| BP | GO:0006690 | icosanoid metabolic process | 0.002587 | 0.01729 | 7 |
| BP | GO:0060997 | dendritic spine morphogenesis | 0.002597 | 0.017294 | 5 |
| BP | GO:1905330 | regulation of morphogenesis of an epithelium | 0.002597 | 0.017294 | 5 |
| BP | GO:0034614 | cellular response to reactive oxygen species | 0.002691 | 0.017892 | 7 |
| BP | GO:0086009 | membrane repolarization | 0.002718 | 0.01804 | 4 |
| BP | GO:0003300 | cardiac muscle hypertrophy | 0.002741 | 0.018164 | 6 |
| BP | GO:1903522 | regulation of blood circulation | 0.002767 | 0.018232 | 10 |
| BP | GO:0042730 | fibrinolysis | 0.002767 | 0.018232 | 3 |
| BP | GO:0150104 | transport across blood-brain barrier | 0.002767 | 0.018232 | 3 |
| BP | GO:0007369 | gastrulation | 0.002769 | 0.018232 | 8 |
| BP | GO:1990874 | vascular associated smooth muscle cell proliferation | 0.002922 | 0.019204 | 5 |
| BP | GO:0030279 | negative regulation of ossification | 0.002947 | 0.019335 | 4 |
| BP | GO:0097553 | calcium ion transmembrane import into cytosol | 0.00296 | 0.019391 | 8 |
| BP | GO:0034446 | substrate adhesion-dependent cell spreading | 0.003006 | 0.01966 | 6 |
| BP | GO:0032409 | regulation of transporter activity | 0.003035 | 0.01982 | 11 |
| BP | GO:0048813 | dendrite morphogenesis | 0.003059 | 0.019941 | 8 |
| BP | GO:0061045 | negative regulation of wound healing | 0.003095 | 0.020143 | 5 |
| BP | GO:0010469 | regulation of signaling receptor activity | 0.003138 | 0.020273 | 7 |
| BP | GO:0120161 | regulation of cold-induced thermogenesis | 0.003138 | 0.020273 | 7 |
| BP | GO:0010595 | positive regulation of endothelial cell migration | 0.003146 | 0.020273 | 6 |
| BP | GO:0010232 | vascular transport | 0.003152 | 0.020273 | 3 |
| BP | GO:0072677 | eosinophil migration | 0.003152 | 0.020273 | 3 |
| BP | GO:1901623 | regulation of lymphocyte chemotaxis | 0.003152 | 0.020273 | 3 |
| BP | GO:2001044 | regulation of integrin-mediated signaling pathway | 0.003152 | 0.020273 | 3 |
| BP | GO:0043409 | negative regulation of MAPK cascade | 0.003161 | 0.020273 | 8 |
| BP | GO:0048592 | eye morphogenesis | 0.003161 | 0.020273 | 8 |
| BP | GO:0043403 | skeletal muscle tissue regeneration | 0.003188 | 0.020417 | 4 |
| BP | GO:1903038 | negative regulation of leukocyte cell-cell adhesion | 0.003258 | 0.020828 | 7 |
| BP | GO:0016331 | morphogenesis of embryonic epithelium | 0.003265 | 0.020841 | 8 |
| BP | GO:0007492 | endoderm development | 0.003275 | 0.020875 | 5 |
| BP | GO:0014897 | striated muscle hypertrophy | 0.00329 | 0.020933 | 6 |
| BP | GO:0032956 | regulation of actin cytoskeleton organization | 0.003317 | 0.021073 | 12 |
| BP | GO:0007611 | learning or memory | 0.003337 | 0.021156 | 11 |
| BP | GO:0050920 | regulation of chemotaxis | 0.003341 | 0.021156 | 9 |
| BP | GO:0106106 | cold-induced thermogenesis | 0.003381 | 0.021378 | 7 |
| BP | GO:0001843 | neural tube closure | 0.003439 | 0.021675 | 6 |
| BP | GO:0071346 | cellular response to interferon-gamma | 0.003439 | 0.021675 | 6 |
| BP | GO:0002292 | T cell differentiation involved in immune response | 0.003463 | 0.02176 | 5 |
| BP | GO:2000379 | positive regulation of reactive oxygen species metabolic process | 0.003463 | 0.02176 | 5 |
| BP | GO:0000302 | response to reactive oxygen species | 0.003481 | 0.021838 | 8 |
| BP | GO:0009991 | response to extracellular stimulus | 0.003556 | 0.022058 | 13 |
| BP | GO:0003417 | growth plate cartilage development | 0.003568 | 0.022058 | 3 |
| BP | GO:0009190 | cyclic nucleotide biosynthetic process | 0.003568 | 0.022058 | 3 |
| BP | GO:0042026 | protein refolding | 0.003568 | 0.022058 | 3 |
| BP | GO:0052652 | cyclic purine nucleotide metabolic process | 0.003568 | 0.022058 | 3 |
| BP | GO:0009895 | negative regulation of catabolic process | 0.003579 | 0.022058 | 11 |
| BP | GO:0022409 | positive regulation of cell-cell adhesion | 0.003579 | 0.022058 | 11 |
| BP | GO:0045927 | positive regulation of growth | 0.003579 | 0.022058 | 11 |
| BP | GO:0055074 | calcium ion homeostasis | 0.003579 | 0.022058 | 11 |
| BP | GO:0071375 | cellular response to peptide hormone stimulus | 0.003588 | 0.022058 | 10 |
| BP | GO:0014896 | muscle hypertrophy | 0.003593 | 0.022058 | 6 |
| BP | GO:0031532 | actin cytoskeleton reorganization | 0.003593 | 0.022058 | 6 |
| BP | GO:0060606 | tube closure | 0.003593 | 0.022058 | 6 |
| BP | GO:0050731 | positive regulation of peptidyl-tyrosine phosphorylation | 0.003593 | 0.022058 | 8 |
| BP | GO:0003206 | cardiac chamber morphogenesis | 0.003639 | 0.022302 | 7 |
| BP | GO:0045669 | positive regulation of osteoblast differentiation | 0.003659 | 0.02239 | 5 |
| BP | GO:0010543 | regulation of platelet activation | 0.003711 | 0.022608 | 4 |
| BP | GO:0010762 | regulation of fibroblast migration | 0.003711 | 0.022608 | 4 |
| BP | GO:0045214 | sarcomere organization | 0.003711 | 0.022608 | 4 |
| BP | GO:0030217 | T cell differentiation | 0.003748 | 0.022797 | 11 |
| BP | GO:0070663 | regulation of leukocyte proliferation | 0.003774 | 0.022917 | 10 |
| BP | GO:0050777 | negative regulation of immune response | 0.003947 | 0.023898 | 8 |
| BP | GO:0050905 | neuromuscular process | 0.003947 | 0.023898 | 8 |
| BP | GO:0010862 | positive regulation of pathway-restricted SMAD protein phosphorylation | 0.003994 | 0.024025 | 4 |
| BP | GO:0045429 | positive regulation of nitric oxide biosynthetic process | 0.003994 | 0.024025 | 4 |
| BP | GO:0010640 | regulation of platelet-derived growth factor receptor signaling pathway | 0.004016 | 0.024025 | 3 |
| BP | GO:0010759 | positive regulation of macrophage chemotaxis | 0.004016 | 0.024025 | 3 |
| BP | GO:0010763 | positive regulation of fibroblast migration | 0.004016 | 0.024025 | 3 |
| BP | GO:0035357 | peroxisome proliferator activated receptor signaling pathway | 0.004016 | 0.024025 | 3 |
| BP | GO:0045063 | T-helper 1 cell differentiation | 0.004016 | 0.024025 | 3 |
| BP | GO:1903975 | regulation of glial cell migration | 0.004016 | 0.024025 | 3 |
| BP | GO:0006633 | fatty acid biosynthetic process | 0.004053 | 0.024209 | 7 |
| BP | GO:1904035 | regulation of epithelial cell apoptotic process | 0.004086 | 0.024371 | 6 |
| BP | GO:0042129 | regulation of T cell proliferation | 0.004197 | 0.02496 | 8 |
| BP | GO:2001257 | regulation of cation channel activity | 0.004197 | 0.02496 | 8 |
| BP | GO:0009185 | ribonucleoside diphosphate metabolic process | 0.004261 | 0.025263 | 6 |
| BP | GO:0060078 | regulation of postsynaptic membrane potential | 0.004261 | 0.025263 | 6 |
| BP | GO:0045088 | regulation of innate immune response | 0.004288 | 0.025321 | 9 |
| BP | GO:0046456 | icosanoid biosynthetic process | 0.00429 | 0.025321 | 4 |
| BP | GO:1902041 | regulation of extrinsic apoptotic signaling pathway via death domain receptors | 0.00429 | 0.025321 | 4 |
| BP | GO:0008154 | actin polymerization or depolymerization | 0.00446 | 0.026247 | 8 |
| BP | GO:0098739 | import across plasma membrane | 0.00446 | 0.026247 | 8 |
| BP | GO:0001822 | kidney development | 0.004489 | 0.026348 | 11 |
| BP | GO:0030449 | regulation of complement activation | 0.004497 | 0.026348 | 3 |
| BP | GO:0099623 | regulation of cardiac muscle cell membrane repolarization | 0.004497 | 0.026348 | 3 |
| BP | GO:0008625 | extrinsic apoptotic signaling pathway via death domain receptors | 0.00452 | 0.02637 | 5 |
| BP | GO:0032722 | positive regulation of chemokine production | 0.00452 | 0.02637 | 5 |
| BP | GO:0070373 | negative regulation of ERK1 and ERK2 cascade | 0.00452 | 0.02637 | 5 |
| BP | GO:0045773 | positive regulation of axon extension | 0.004601 | 0.026683 | 4 |
| BP | GO:0060251 | regulation of glial cell proliferation | 0.004601 | 0.026683 | 4 |
| BP | GO:1903426 | regulation of reactive oxygen species biosynthetic process | 0.004601 | 0.026683 | 4 |
| BP | GO:1904407 | positive regulation of nitric oxide metabolic process | 0.004601 | 0.026683 | 4 |
| BP | GO:0014020 | primary neural tube formation | 0.004628 | 0.0268 | 6 |
| BP | GO:0034109 | homotypic cell-cell adhesion | 0.004756 | 0.027466 | 5 |
| BP | GO:0060998 | regulation of dendritic spine development | 0.004756 | 0.027466 | 5 |
| BP | GO:0002460 | adaptive immune response based on somatic recombination of immune receptors built from immunoglobulin superfamily domains | 0.004769 | 0.027498 | 14 |
| BP | GO:0006888 | endoplasmic reticulum to Golgi vesicle-mediated transport | 0.004819 | 0.02771 | 6 |
| BP | GO:0051209 | release of sequestered calcium ion into cytosol | 0.004819 | 0.02771 | 6 |
| BP | GO:0048762 | mesenchymal cell differentiation | 0.004898 | 0.028119 | 9 |
| BP | GO:0009299 | mRNA transcription | 0.004926 | 0.028121 | 4 |
| BP | GO:0045907 | positive regulation of vasoconstriction | 0.004926 | 0.028121 | 4 |
| BP | GO:0060412 | ventricular septum morphogenesis | 0.004926 | 0.028121 | 4 |
| BP | GO:0061756 | leukocyte adhesion to vascular endothelial cell | 0.004926 | 0.028121 | 4 |
| BP | GO:0014706 | striated muscle tissue development | 0.004933 | 0.028121 | 10 |
| BP | GO:0010634 | positive regulation of epithelial cell migration | 0.004987 | 0.028306 | 7 |
| BP | GO:0043129 | surfactant homeostasis | 0.005011 | 0.028306 | 3 |
| BP | GO:0045723 | positive regulation of fatty acid biosynthetic process | 0.005011 | 0.028306 | 3 |
| BP | GO:0060252 | positive regulation of glial cell proliferation | 0.005011 | 0.028306 | 3 |
| BP | GO:0070168 | negative regulation of biomineral tissue development | 0.005011 | 0.028306 | 3 |
| BP | GO:0010769 | regulation of cell morphogenesis involved in differentiation | 0.005017 | 0.028306 | 6 |
| BP | GO:0051283 | negative regulation of sequestering of calcium ion | 0.005017 | 0.028306 | 6 |
| BP | GO:0002695 | negative regulation of leukocyte activation | 0.005022 | 0.028306 | 8 |
| BP | GO:0072006 | nephron development | 0.005158 | 0.029029 | 7 |
| BP | GO:0032412 | regulation of ion transmembrane transporter activity | 0.005171 | 0.029063 | 10 |
| BP | GO:0042542 | response to hydrogen peroxide | 0.00522 | 0.029272 | 6 |
| BP | GO:0003151 | outflow tract morphogenesis | 0.005255 | 0.029272 | 5 |
| BP | GO:0019229 | regulation of vasoconstriction | 0.005255 | 0.029272 | 5 |
| BP | GO:0048041 | focal adhesion assembly | 0.005255 | 0.029272 | 5 |
| BP | GO:0006692 | prostanoid metabolic process | 0.005267 | 0.029272 | 4 |
| BP | GO:0006693 | prostaglandin metabolic process | 0.005267 | 0.029272 | 4 |
| BP | GO:0045687 | positive regulation of glial cell differentiation | 0.005267 | 0.029272 | 4 |
| BP | GO:2000351 | regulation of endothelial cell apoptotic process | 0.005267 | 0.029272 | 4 |
| BP | GO:0015850 | organic hydroxy compound transport | 0.005293 | 0.029338 | 10 |
| BP | GO:0042593 | glucose homeostasis | 0.005293 | 0.029338 | 10 |
| BP | GO:0033500 | carbohydrate homeostasis | 0.005418 | 0.029926 | 10 |
| BP | GO:0001676 | long-chain fatty acid metabolic process | 0.00543 | 0.029926 | 6 |
| BP | GO:0030316 | osteoclast differentiation | 0.00543 | 0.029926 | 6 |
| BP | GO:0051282 | regulation of sequestering of calcium ion | 0.00543 | 0.029926 | 6 |
| BP | GO:0046890 | regulation of lipid biosynthetic process | 0.005479 | 0.030154 | 8 |
| BP | GO:0048708 | astrocyte differentiation | 0.005517 | 0.030305 | 5 |
| BP | GO:0003094 | glomerular filtration | 0.005559 | 0.030305 | 3 |
| BP | GO:0003299 | muscle hypertrophy in response to stress | 0.005559 | 0.030305 | 3 |
| BP | GO:0014898 | cardiac muscle hypertrophy in response to stress | 0.005559 | 0.030305 | 3 |
| BP | GO:0090103 | cochlea morphogenesis | 0.005559 | 0.030305 | 3 |
| BP | GO:0110150 | negative regulation of biomineralization | 0.005559 | 0.030305 | 3 |
| BP | GO:2000310 | regulation of NMDA receptor activity | 0.005559 | 0.030305 | 3 |
| BP | GO:0001974 | blood vessel remodeling | 0.005623 | 0.030447 | 4 |
| BP | GO:0042149 | cellular response to glucose starvation | 0.005623 | 0.030447 | 4 |
| BP | GO:0070527 | platelet aggregation | 0.005623 | 0.030447 | 4 |
| BP | GO:1902622 | regulation of neutrophil migration | 0.005623 | 0.030447 | 4 |
| BP | GO:2000378 | negative regulation of reactive oxygen species metabolic process | 0.005623 | 0.030447 | 4 |
| BP | GO:0051048 | negative regulation of secretion | 0.005637 | 0.030483 | 8 |
| BP | GO:1905954 | positive regulation of lipid localization | 0.005645 | 0.030483 | 6 |
| BP | GO:2001236 | regulation of extrinsic apoptotic signaling pathway | 0.005695 | 0.03071 | 7 |
| BP | GO:0009259 | ribonucleotide metabolic process | 0.005728 | 0.030807 | 13 |
| BP | GO:0034329 | cell junction assembly | 0.005728 | 0.030807 | 13 |
| BP | GO:0003281 | ventricular septum development | 0.005789 | 0.03105 | 5 |
| BP | GO:0021675 | nerve development | 0.005789 | 0.03105 | 5 |
| BP | GO:0046394 | carboxylic acid biosynthetic process | 0.005806 | 0.031098 | 10 |
| BP | GO:0048259 | regulation of receptor-mediated endocytosis | 0.005867 | 0.03134 | 6 |
| BP | GO:0051208 | sequestering of calcium ion | 0.005867 | 0.03134 | 6 |
| BP | GO:0071900 | regulation of protein serine/threonine kinase activity | 0.005936 | 0.031645 | 11 |
| BP | GO:0016053 | organic acid biosynthetic process | 0.005939 | 0.031645 | 10 |
| BP | GO:0072001 | renal system development | 0.00606 | 0.03221 | 11 |
| BP | GO:0001738 | morphogenesis of a polarized epithelium | 0.00607 | 0.03221 | 5 |
| BP | GO:1900542 | regulation of purine nucleotide metabolic process | 0.00607 | 0.03221 | 5 |
| BP | GO:0032535 | regulation of cellular component size | 0.006124 | 0.032249 | 12 |
| BP | GO:0072330 | monocarboxylic acid biosynthetic process | 0.006135 | 0.032249 | 8 |
| BP | GO:0001780 | neutrophil homeostasis | 0.006142 | 0.032249 | 3 |
| BP | GO:0014887 | cardiac muscle adaptation | 0.006142 | 0.032249 | 3 |
| BP | GO:0048875 | chemical homeostasis within a tissue | 0.006142 | 0.032249 | 3 |
| BP | GO:0061003 | positive regulation of dendritic spine morphogenesis | 0.006142 | 0.032249 | 3 |
| BP | GO:0090023 | positive regulation of neutrophil chemotaxis | 0.006142 | 0.032249 | 3 |
| BP | GO:1902235 | regulation of endoplasmic reticulum stress-induced intrinsic apoptotic signaling pathway | 0.006142 | 0.032249 | 3 |
| BP | GO:0017157 | regulation of exocytosis | 0.006163 | 0.03232 | 9 |
| BP | GO:1902105 | regulation of leukocyte differentiation | 0.006187 | 0.032405 | 11 |
| BP | GO:0006937 | regulation of muscle contraction | 0.006273 | 0.032765 | 7 |
| BP | GO:0032675 | regulation of interleukin-6 production | 0.006273 | 0.032765 | 7 |
| BP | GO:0030514 | negative regulation of BMP signaling pathway | 0.006383 | 0.032995 | 4 |
| BP | GO:0031646 | positive regulation of nervous system process | 0.006383 | 0.032995 | 4 |
| BP | GO:0034113 | heterotypic cell-cell adhesion | 0.006383 | 0.032995 | 4 |
| BP | GO:0043536 | positive regulation of blood vessel endothelial cell migration | 0.006383 | 0.032995 | 4 |
| BP | GO:0044060 | regulation of endocrine process | 0.006383 | 0.032995 | 4 |
| BP | GO:0050732 | negative regulation of peptidyl-tyrosine phosphorylation | 0.006383 | 0.032995 | 4 |
| BP | GO:0072577 | endothelial cell apoptotic process | 0.006383 | 0.032995 | 4 |
| BP | GO:0110151 | positive regulation of biomineralization | 0.006383 | 0.032995 | 4 |
| BP | GO:0043087 | regulation of GTPase activity | 0.006448 | 0.033267 | 11 |
| BP | GO:0048015 | phosphatidylinositol-mediated signaling | 0.006475 | 0.033267 | 7 |
| BP | GO:2000058 | regulation of ubiquitin-dependent protein catabolic process | 0.006475 | 0.033267 | 7 |
| BP | GO:0008361 | regulation of cell size | 0.006485 | 0.033267 | 8 |
| BP | GO:0010721 | negative regulation of cell development | 0.006485 | 0.033267 | 8 |
| BP | GO:0030307 | positive regulation of cell growth | 0.006485 | 0.033267 | 8 |
| BP | GO:0022898 | regulation of transmembrane transporter activity | 0.006499 | 0.033296 | 10 |
| BP | GO:0001952 | regulation of cell-matrix adhesion | 0.006569 | 0.033526 | 6 |
| BP | GO:0003279 | cardiac septum development | 0.006569 | 0.033526 | 6 |
| BP | GO:0030048 | actin filament-based movement | 0.006569 | 0.033526 | 6 |
| BP | GO:0032691 | negative regulation of interleukin-1 beta production | 0.006759 | 0.034233 | 3 |
| BP | GO:0046885 | regulation of hormone biosynthetic process | 0.006759 | 0.034233 | 3 |
| BP | GO:0071353 | cellular response to interleukin-4 | 0.006759 | 0.034233 | 3 |
| BP | GO:0086019 | cell-cell signaling involved in cardiac conduction | 0.006759 | 0.034233 | 3 |
| BP | GO:0097205 | renal filtration | 0.006759 | 0.034233 | 3 |
| BP | GO:1905562 | regulation of vascular endothelial cell proliferation | 0.006759 | 0.034233 | 3 |
| BP | GO:0043030 | regulation of macrophage activation | 0.006787 | 0.034285 | 4 |
| BP | GO:0140353 | lipid export from cell | 0.006787 | 0.034285 | 4 |
| BP | GO:2000278 | regulation of DNA biosynthetic process | 0.006817 | 0.034393 | 6 |
| BP | GO:0007292 | female gamete generation | 0.006893 | 0.034691 | 7 |
| BP | GO:0071248 | cellular response to metal ion | 0.006893 | 0.034691 | 7 |
| BP | GO:0048511 | rhythmic process | 0.006945 | 0.034742 | 10 |
| BP | GO:0006096 | glycolytic process | 0.00697 | 0.034742 | 5 |
| BP | GO:0032436 | positive regulation of proteasomal ubiquitin-dependent protein catabolic process | 0.00697 | 0.034742 | 5 |
| BP | GO:0019693 | ribose phosphate metabolic process | 0.00697 | 0.034742 | 13 |
| BP | GO:0002158 | osteoclast proliferation | 0.007034 | 0.034742 | 2 |
| BP | GO:0003149 | membranous septum morphogenesis | 0.007034 | 0.034742 | 2 |
| BP | GO:0035360 | positive regulation of peroxisome proliferator activated receptor signaling pathway | 0.007034 | 0.034742 | 2 |
| BP | GO:0043615 | astrocyte cell migration | 0.007034 | 0.034742 | 2 |
| BP | GO:0060022 | hard palate development | 0.007034 | 0.034742 | 2 |
| BP | GO:0060392 | negative regulation of SMAD protein signal transduction | 0.007034 | 0.034742 | 2 |
| BP | GO:0072674 | multinuclear osteoclast differentiation | 0.007034 | 0.034742 | 2 |
| BP | GO:0098883 | synapse pruning | 0.007034 | 0.034742 | 2 |
| BP | GO:0099640 | axo-dendritic protein transport | 0.007034 | 0.034742 | 2 |
| BP | GO:1900147 | regulation of Schwann cell migration | 0.007034 | 0.034742 | 2 |
| BP | GO:1901724 | positive regulation of cell proliferation involved in kidney development | 0.007034 | 0.034742 | 2 |
| BP | GO:0043406 | positive regulation of MAP kinase activity | 0.007071 | 0.034882 | 6 |
| BP | GO:0048017 | inositol lipid-mediated signaling | 0.00711 | 0.034945 | 7 |
| BP | GO:0051250 | negative regulation of lymphocyte activation | 0.00711 | 0.034945 | 7 |
| BP | GO:0061025 | membrane fusion | 0.00711 | 0.034945 | 7 |
| BP | GO:0042743 | hydrogen peroxide metabolic process | 0.007207 | 0.035338 | 4 |
| BP | GO:0050775 | positive regulation of dendrite morphogenesis | 0.007207 | 0.035338 | 4 |
| BP | GO:0071496 | cellular response to external stimulus | 0.007256 | 0.035531 | 10 |
| BP | GO:0006757 | ATP generation from ADP | 0.007289 | 0.035609 | 5 |
| BP | GO:0048814 | regulation of dendrite morphogenesis | 0.007289 | 0.035609 | 5 |
| BP | GO:0006829 | zinc ion transport | 0.007413 | 0.035947 | 3 |
| BP | GO:0033688 | regulation of osteoblast proliferation | 0.007413 | 0.035947 | 3 |
| BP | GO:0035767 | endothelial cell chemotaxis | 0.007413 | 0.035947 | 3 |
| BP | GO:0060384 | innervation | 0.007413 | 0.035947 | 3 |
| BP | GO:0071624 | positive regulation of granulocyte chemotaxis | 0.007413 | 0.035947 | 3 |
| BP | GO:0101023 | vascular endothelial cell proliferation | 0.007413 | 0.035947 | 3 |
| BP | GO:0050890 | cognition | 0.007427 | 0.035972 | 11 |
| BP | GO:0006066 | alcohol metabolic process | 0.007576 | 0.036649 | 11 |
| BP | GO:0035914 | skeletal muscle cell differentiation | 0.007619 | 0.03677 | 5 |
| BP | GO:1903035 | negative regulation of response to wounding | 0.007619 | 0.03677 | 5 |
| BP | GO:0051930 | regulation of sensory perception of pain | 0.007645 | 0.03685 | 4 |
| BP | GO:1990830 | cellular response to leukemia inhibitory factor | 0.007741 | 0.03727 | 10 |
| BP | GO:0035265 | organ growth | 0.00783 | 0.037608 | 8 |
| BP | GO:0048639 | positive regulation of developmental growth | 0.00783 | 0.037608 | 8 |
| BP | GO:1901216 | positive regulation of neuron death | 0.007874 | 0.03777 | 6 |
| BP | GO:1990823 | response to leukemia inhibitory factor | 0.007908 | 0.037892 | 10 |
| BP | GO:0006140 | regulation of nucleotide metabolic process | 0.007959 | 0.038089 | 5 |
| BP | GO:0051896 | regulation of protein kinase B signaling | 0.008027 | 0.038368 | 7 |
| BP | GO:0001666 | response to hypoxia | 0.008038 | 0.038377 | 8 |
| BP | GO:0051931 | regulation of sensory perception | 0.008099 | 0.038451 | 4 |
| BP | GO:0048011 | neurotrophin TRK receptor signaling pathway | 0.008102 | 0.038451 | 3 |
| BP | GO:0060390 | regulation of SMAD protein signal transduction | 0.008102 | 0.038451 | 3 |
| BP | GO:0061050 | regulation of cell growth involved in cardiac muscle cell development | 0.008102 | 0.038451 | 3 |
| BP | GO:0070670 | response to interleukin-4 | 0.008102 | 0.038451 | 3 |
| BP | GO:0001841 | neural tube formation | 0.008155 | 0.038566 | 6 |
| BP | GO:0014065 | phosphatidylinositol 3-kinase signaling | 0.008155 | 0.038566 | 6 |
| BP | GO:0050868 | negative regulation of T cell activation | 0.008155 | 0.038566 | 6 |
| BP | GO:0032635 | interleukin-6 production | 0.008269 | 0.039059 | 7 |
| BP | GO:0016358 | dendrite development | 0.008427 | 0.039757 | 10 |
| BP | GO:0048839 | inner ear development | 0.008467 | 0.039835 | 8 |
| BP | GO:1903531 | negative regulation of secretion by cell | 0.008517 | 0.039835 | 7 |
| BP | GO:0033212 | iron import into cell | 0.008523 | 0.039835 | 2 |
| BP | GO:0034351 | negative regulation of glial cell apoptotic process | 0.008523 | 0.039835 | 2 |
| BP | GO:0036135 | Schwann cell migration | 0.008523 | 0.039835 | 2 |
| BP | GO:0043568 | positive regulation of insulin-like growth factor receptor signaling pathway | 0.008523 | 0.039835 | 2 |
| BP | GO:0061517 | macrophage proliferation | 0.008523 | 0.039835 | 2 |
| BP | GO:0097278 | complement-dependent cytotoxicity | 0.008523 | 0.039835 | 2 |
| BP | GO:1903078 | positive regulation of protein localization to plasma membrane | 0.008571 | 0.040013 | 4 |
| BP | GO:0070252 | actin-mediated cell contraction | 0.00867 | 0.040427 | 5 |
| BP | GO:0009132 | nucleoside diphosphate metabolic process | 0.00874 | 0.040706 | 6 |
| BP | GO:0002675 | positive regulation of acute inflammatory response | 0.008828 | 0.040876 | 3 |
| BP | GO:0009187 | cyclic nucleotide metabolic process | 0.008828 | 0.040876 | 3 |
| BP | GO:0040036 | regulation of fibroblast growth factor receptor signaling pathway | 0.008828 | 0.040876 | 3 |
| BP | GO:1904706 | negative regulation of vascular associated smooth muscle cell proliferation | 0.008828 | 0.040876 | 3 |
| BP | GO:1905314 | semi-lunar valve development | 0.008828 | 0.040876 | 3 |
| BP | GO:0030098 | lymphocyte differentiation | 0.008998 | 0.041618 | 13 |
| BP | GO:0001736 | establishment of planar polarity | 0.009061 | 0.04181 | 4 |
| BP | GO:1903409 | reactive oxygen species biosynthetic process | 0.009061 | 0.04181 | 4 |
| BP | GO:0048738 | cardiac muscle tissue development | 0.009225 | 0.042518 | 9 |
| BP | GO:1902905 | positive regulation of supramolecular fiber organization | 0.009293 | 0.04278 | 7 |
| BP | GO:0002699 | positive regulation of immune effector process | 0.009349 | 0.04299 | 10 |
| BP | GO:0019935 | cyclic-nucleotide-mediated signaling | 0.009424 | 0.043287 | 5 |
| BP | GO:1903039 | positive regulation of leukocyte cell-cell adhesion | 0.009436 | 0.043289 | 9 |
| BP | GO:0006887 | exocytosis | 0.009509 | 0.043575 | 12 |
| BP | GO:0051222 | positive regulation of protein transport | 0.009542 | 0.043678 | 10 |
| BP | GO:0007164 | establishment of tissue polarity | 0.009568 | 0.043699 | 4 |
| BP | GO:0044319 | wound healing; spreading of cells | 0.009591 | 0.043699 | 3 |
| BP | GO:0060306 | regulation of membrane repolarization | 0.009591 | 0.043699 | 3 |
| BP | GO:0090505 | epiboly involved in wound healing | 0.009591 | 0.043699 | 3 |
| BP | GO:0034341 | response to interferon-gamma | 0.009673 | 0.044024 | 6 |
| BP | GO:0045621 | positive regulation of lymphocyte differentiation | 0.009999 | 0.045076 | 6 |
| BP | GO:0009150 | purine ribonucleotide metabolic process | 0.010018 | 0.045076 | 12 |
| BP | GO:0010656 | negative regulation of muscle cell apoptotic process | 0.010094 | 0.045076 | 4 |
| BP | GO:0006047 | UDP-N-acetylglucosamine metabolic process | 0.010141 | 0.045076 | 2 |
| BP | GO:0006182 | cGMP biosynthetic process | 0.010141 | 0.045076 | 2 |
| BP | GO:0007168 | receptor guanylyl cyclase signaling pathway | 0.010141 | 0.045076 | 2 |
| BP | GO:0009404 | toxin metabolic process | 0.010141 | 0.045076 | 2 |
| BP | GO:0010572 | positive regulation of platelet activation | 0.010141 | 0.045076 | 2 |
| BP | GO:0015911 | long-chain fatty acid import across plasma membrane | 0.010141 | 0.045076 | 2 |
| BP | GO:0033089 | positive regulation of T cell differentiation in thymus | 0.010141 | 0.045076 | 2 |
| BP | GO:0033210 | leptin-mediated signaling pathway | 0.010141 | 0.045076 | 2 |
| BP | GO:0035437 | maintenance of protein localization in endoplasmic reticulum | 0.010141 | 0.045076 | 2 |
| BP | GO:0045348 | positive regulation of MHC class II biosynthetic process | 0.010141 | 0.045076 | 2 |
| BP | GO:0045625 | regulation of T-helper 1 cell differentiation | 0.010141 | 0.045076 | 2 |
| BP | GO:0071732 | cellular response to nitric oxide | 0.010141 | 0.045076 | 2 |
| BP | GO:0072124 | regulation of glomerular mesangial cell proliferation | 0.010141 | 0.045076 | 2 |
| BP | GO:0086013 | membrane repolarization during cardiac muscle cell action potential | 0.010141 | 0.045076 | 2 |
| BP | GO:0099519 | dense core granule cytoskeletal transport | 0.010141 | 0.045076 | 2 |
| BP | GO:1900102 | negative regulation of endoplasmic reticulum unfolded protein response | 0.010141 | 0.045076 | 2 |
| BP | GO:1903028 | positive regulation of opsonization | 0.010141 | 0.045076 | 2 |
| BP | GO:2001223 | negative regulation of neuron migration | 0.010141 | 0.045076 | 2 |
| BP | GO:0007200 | phospholipase C-activating G protein-coupled receptor signaling pathway | 0.010222 | 0.045286 | 5 |
| BP | GO:0014066 | regulation of phosphatidylinositol 3-kinase signaling | 0.010222 | 0.045286 | 5 |
| BP | GO:0032147 | activation of protein kinase activity | 0.010222 | 0.045286 | 5 |
| BP | GO:0014002 | astrocyte development | 0.010391 | 0.045879 | 3 |
| BP | GO:0048679 | regulation of axon regeneration | 0.010391 | 0.045879 | 3 |
| BP | GO:0090504 | epiboly | 0.010391 | 0.045879 | 3 |
| BP | GO:0015909 | long-chain fatty acid transport | 0.010638 | 0.046714 | 4 |
| BP | GO:0032835 | glomerulus development | 0.010638 | 0.046714 | 4 |
| BP | GO:0048286 | lung alveolus development | 0.010638 | 0.046714 | 4 |
| BP | GO:0009408 | response to heat | 0.010638 | 0.046714 | 5 |
| BP | GO:0097061 | dendritic spine organization | 0.010638 | 0.046714 | 5 |
| BP | GO:0035148 | tube formation | 0.010698 | 0.046876 | 7 |
| BP | GO:0071897 | DNA biosynthetic process | 0.010698 | 0.046876 | 7 |
| BP | GO:0072521 | purine-containing compound metabolic process | 0.010933 | 0.04785 | 13 |
| BP | GO:0035051 | cardiocyte differentiation | 0.010997 | 0.04808 | 7 |
| BP | GO:0110053 | regulation of actin filament organization | 0.011012 | 0.048091 | 9 |
| BP | GO:0010770 | positive regulation of cell morphogenesis involved in differentiation | 0.011065 | 0.048272 | 5 |
| BP | GO:0010043 | response to zinc ion | 0.011228 | 0.048876 | 3 |
| BP | GO:0014912 | negative regulation of smooth muscle cell migration | 0.011228 | 0.048876 | 3 |
| BP | GO:0032388 | positive regulation of intracellular transport | 0.011302 | 0.04909 | 7 |
| BP | GO:1905952 | regulation of lipid localization | 0.011302 | 0.04909 | 7 |
| BP | GO:0007173 | epidermal growth factor receptor signaling pathway | 0.011504 | 0.049806 | 5 |
| BP | GO:0055013 | cardiac muscle cell development | 0.011504 | 0.049806 | 5 |
| BP | GO:0090630 | activation of GTPase activity | 0.011504 | 0.049806 | 5 |
| CC | GO:0062023 | collagen-containing extracellular matrix | 1.29E-45 | 4.59E-43 | 60 |
| CC | GO:0005581 | collagen trimer | 1.03E-21 | 1.84E-19 | 21 |
| CC | GO:0005604 | basement membrane | 3.62E-13 | 2.64E-11 | 17 |
| CC | GO:0005583 | fibrillar collagen trimer | 3.70E-13 | 2.64E-11 | 8 |
| CC | GO:0098643 | banded collagen fibril | 3.70E-13 | 2.64E-11 | 8 |
| CC | GO:0098644 | complex of collagen trimers | 8.60E-11 | 5.12E-09 | 8 |
| CC | GO:0030141 | secretory granule | 1.01E-09 | 5.16E-08 | 25 |
| CC | GO:0031252 | cell leading edge | 5.82E-09 | 2.60E-07 | 23 |
| CC | GO:0042383 | sarcolemma | 4.30E-08 | 1.71E-06 | 14 |
| CC | GO:0030133 | transport vesicle | 1.81E-07 | 6.45E-06 | 19 |
| CC | GO:0043292 | contractile fiber | 5.78E-07 | 1.88E-05 | 15 |
| CC | GO:0001726 | ruffle | 7.57E-07 | 2.25E-05 | 12 |
| CC | GO:0005884 | actin filament | 1.59E-06 | 4.01E-05 | 11 |
| CC | GO:0030016 | myofibril | 1.59E-06 | 4.01E-05 | 14 |
| CC | GO:0070382 | exocytic vesicle | 1.68E-06 | 4.01E-05 | 15 |
| CC | GO:0030017 | sarcomere | 2.29E-06 | 5.10E-05 | 13 |
| CC | GO:0031674 | I band | 3.22E-06 | 6.76E-05 | 11 |
| CC | GO:0045121 | membrane raft | 5.18E-06 | 9.12E-05 | 18 |
| CC | GO:0032432 | actin filament bundle | 5.20E-06 | 9.12E-05 | 9 |
| CC | GO:0042641 | actomyosin | 5.20E-06 | 9.12E-05 | 9 |
| CC | GO:0098857 | membrane microdomain | 5.36E-06 | 9.12E-05 | 18 |
| CC | GO:0019897 | extrinsic component of plasma membrane | 8.31E-06 | 0.000135 | 12 |
| CC | GO:0030018 | Z disc | 9.07E-06 | 0.000141 | 10 |
| CC | GO:0034663 | endoplasmic reticulum chaperone complex | 1.30E-05 | 0.000189 | 4 |
| CC | GO:0098802 | plasma membrane signaling receptor complex | 1.33E-05 | 0.000189 | 12 |
| CC | GO:0001725 | stress fiber | 2.03E-05 | 0.000268 | 8 |
| CC | GO:0097517 | contractile actin filament bundle | 2.03E-05 | 0.000268 | 8 |
| CC | GO:0030667 | secretory granule membrane | 2.59E-05 | 0.00033 | 8 |
| CC | GO:0005614 | interstitial matrix | 5.95E-05 | 0.000732 | 4 |
| CC | GO:0098636 | protein complex involved in cell adhesion | 8.90E-05 | 0.001059 | 6 |
| CC | GO:0005925 | focal adhesion | 9.39E-05 | 0.001081 | 10 |
| CC | GO:0005790 | smooth endoplasmic reticulum | 9.97E-05 | 0.001113 | 5 |
| CC | GO:0031091 | platelet alpha granule | 0.000143 | 0.001552 | 4 |
| CC | GO:0019898 | extrinsic component of membrane | 0.000157 | 0.001645 | 14 |
| CC | GO:0030055 | cell-substrate junction | 0.000171 | 0.001743 | 10 |
| CC | GO:0043235 | receptor complex | 0.000192 | 0.001908 | 16 |
| CC | GO:0005798 | Golgi-associated vesicle | 0.000236 | 0.002278 | 7 |
| CC | GO:0032127 | dense core granule membrane | 0.000247 | 0.002324 | 3 |
| CC | GO:0005901 | caveola | 0.000269 | 0.002461 | 7 |
| CC | GO:0032593 | insulin-responsive compartment | 0.000337 | 0.003006 | 3 |
| CC | GO:0030135 | coated vesicle | 0.00036 | 0.003133 | 10 |
| CC | GO:0070062 | extracellular exosome | 0.000439 | 0.003728 | 7 |
| CC | GO:0030027 | lamellipodium | 0.000515 | 0.004254 | 9 |
| CC | GO:0030137 | COPI-coated vesicle | 0.000524 | 0.004254 | 4 |
| CC | GO:0045335 | phagocytic vesicle | 0.000722 | 0.005602 | 7 |
| CC | GO:0098637 | protein complex involved in cell-matrix adhesion | 0.000722 | 0.005602 | 3 |
| CC | GO:0005788 | endoplasmic reticulum lumen | 0.000874 | 0.006593 | 6 |
| CC | GO:1903561 | extracellular vesicle | 0.000886 | 0.006593 | 7 |
| CC | GO:0098858 | actin-based cell projection | 0.000916 | 0.006673 | 10 |
| CC | GO:0008305 | integrin complex | 0.000972 | 0.006943 | 4 |
| CC | GO:0031256 | leading edge membrane | 0.001063 | 0.007404 | 8 |
| CC | GO:0008021 | synaptic vesicle | 0.001078 | 0.007404 | 10 |
| CC | GO:0005791 | rough endoplasmic reticulum | 0.001111 | 0.007486 | 6 |
| CC | GO:0031225 | anchored component of membrane | 0.001237 | 0.008179 | 9 |
| CC | GO:0030663 | COPI-coated vesicle membrane | 0.00131 | 0.008501 | 3 |
| CC | GO:0043230 | extracellular organelle | 0.001366 | 0.008557 | 7 |
| CC | GO:0065010 | extracellular membrane-bounded organelle | 0.001366 | 0.008557 | 7 |
| CC | GO:0030426 | growth cone | 0.001421 | 0.008746 | 9 |
| CC | GO:0030427 | site of polarized growth | 0.001795 | 0.010541 | 9 |
| CC | GO:0043034 | costamere | 0.001831 | 0.010541 | 3 |
| CC | GO:1905286 | serine-type peptidase complex | 0.001831 | 0.010541 | 3 |
| CC | GO:1905370 | serine-type endopeptidase complex | 0.001831 | 0.010541 | 3 |
| CC | GO:0030139 | endocytic vesicle | 0.001915 | 0.010725 | 9 |
| CC | GO:0030175 | filopodium | 0.001923 | 0.010725 | 6 |
| CC | GO:0044853 | plasma membrane raft | 0.002115 | 0.011616 | 7 |
| CC | GO:0043209 | myelin sheath | 0.002175 | 0.011765 | 9 |
| CC | GO:0044295 | axonal growth cone | 0.00236 | 0.012575 | 4 |
| CC | GO:0045111 | intermediate filament cytoskeleton | 0.002444 | 0.012832 | 8 |
| CC | GO:0005882 | intermediate filament | 0.00249 | 0.012873 | 7 |
| CC | GO:0032587 | ruffle membrane | 0.002524 | 0.012873 | 5 |
| CC | GO:0034358 | plasma lipoprotein particle | 0.003274 | 0.016236 | 4 |
| CC | GO:1990777 | lipoprotein particle | 0.003274 | 0.016236 | 4 |
| CC | GO:0009925 | basal plasma membrane | 0.003503 | 0.017129 | 10 |
| CC | GO:0005793 | endoplasmic reticulum-Golgi intermediate compartment | 0.003575 | 0.017246 | 5 |
| CC | GO:0016324 | apical plasma membrane | 0.003675 | 0.017492 | 12 |
| CC | GO:0032994 | protein-lipid complex | 0.004101 | 0.019013 | 4 |
| CC | GO:0090665 | glycoprotein complex | 0.004101 | 0.019013 | 3 |
| CC | GO:0002102 | podosome | 0.004591 | 0.021014 | 3 |
| CC | GO:0030863 | cortical cytoskeleton | 0.005199 | 0.023494 | 6 |
| CC | GO:0045177 | apical part of cell | 0.005266 | 0.0235 | 14 |
| CC | GO:0030864 | cortical actin cytoskeleton | 0.00542 | 0.02389 | 5 |
| CC | GO:0030662 | coated vesicle membrane | 0.00626 | 0.026858 | 5 |
| CC | GO:0097440 | apical dendrite | 0.006269 | 0.026858 | 3 |
| CC | GO:0045178 | basal part of cell | 0.00639 | 0.026858 | 10 |
| CC | GO:0005769 | early endosome | 0.006395 | 0.026858 | 11 |
| CC | GO:0072562 | blood microparticle | 0.007136 | 0.029622 | 2 |
| CC | GO:0005938 | cell cortex | 0.0073 | 0.029957 | 10 |
| CC | GO:0001533 | cornified envelope | 0.00831 | 0.03371 | 4 |
| CC | GO:0002199 | zona pellucida receptor complex | 0.008647 | 0.034684 | 2 |
| CC | GO:0045171 | intercellular bridge | 0.008936 | 0.035342 | 5 |
| CC | GO:0005921 | gap junction | 0.009009 | 0.035342 | 3 |
| CC | GO:0016528 | sarcoplasm | 0.009318 | 0.036159 | 5 |
| CC | GO:0034703 | cation channel complex | 0.009534 | 0.036597 | 8 |
| CC | GO:0031594 | neuromuscular junction | 0.009712 | 0.036884 | 5 |
| CC | GO:0031253 | cell projection membrane | 0.009816 | 0.036887 | 10 |
| CC | GO:0030140 | trans-Golgi network transport vesicle | 0.010602 | 0.038232 | 3 |
| CC | GO:0031045 | dense core granule | 0.010602 | 0.038232 | 3 |
| CC | GO:0042470 | melanosome | 0.010602 | 0.038232 | 3 |
| CC | GO:0048770 | pigment granule | 0.010602 | 0.038232 | 3 |
| CC | GO:0043204 | perikaryon | 0.011405 | 0.040599 | 6 |
| CC | GO:0014704 | intercalated disc | 0.011486 | 0.040599 | 4 |
| CC | GO:0005916 | fascia adherens | 0.012053 | 0.041777 | 2 |
| CC | GO:0098871 | postsynaptic actin cytoskeleton | 0.012053 | 0.041777 | 2 |
| CC | GO:0016235 | aggresome | 0.013279 | 0.04515 | 3 |
| CC | GO:0031941 | filamentous actin | 0.013279 | 0.04515 | 3 |
| CC | GO:0034362 | low-density lipoprotein particle | 0.013942 | 0.046956 | 2 |
| MF | GO:0005201 | extracellular matrix structural constituent | 2.65E-24 | 1.58E-21 | 28 |
| MF | GO:0030020 | extracellular matrix structural constituent conferring tensile strength | 2.31E-17 | 6.87E-15 | 14 |
| MF | GO:0005539 | glycosaminoglycan binding | 7.32E-16 | 1.45E-13 | 25 |
| MF | GO:0005178 | integrin binding | 1.41E-15 | 2.09E-13 | 21 |
| MF | GO:0008201 | heparin binding | 9.34E-15 | 1.11E-12 | 21 |
| MF | GO:0005518 | collagen binding | 2.20E-12 | 2.18E-10 | 14 |
| MF | GO:0061134 | peptidase regulator activity | 8.14E-12 | 6.92E-10 | 22 |
| MF | GO:0019838 | growth factor binding | 1.72E-11 | 1.28E-09 | 17 |
| MF | GO:0050839 | cell adhesion molecule binding | 2.18E-11 | 1.44E-09 | 23 |
| MF | GO:1901681 | sulfur compound binding | 2.94E-11 | 1.75E-09 | 22 |
| MF | GO:0030414 | peptidase inhibitor activity | 7.30E-09 | 3.95E-07 | 17 |
| MF | GO:0001968 | fibronectin binding | 1.05E-08 | 5.21E-07 | 9 |
| MF | GO:0043394 | proteoglycan binding | 3.68E-08 | 1.68E-06 | 9 |
| MF | GO:0002020 | protease binding | 1.62E-07 | 6.88E-06 | 14 |
| MF | GO:0048407 | platelet-derived growth factor binding | 2.68E-07 | 1.06E-05 | 5 |
| MF | GO:0061135 | endopeptidase regulator activity | 4.52E-07 | 1.68E-05 | 15 |
| MF | GO:0008083 | growth factor activity | 6.40E-07 | 2.17E-05 | 12 |
| MF | GO:0004857 | enzyme inhibitor activity | 6.57E-07 | 2.17E-05 | 20 |
| MF | GO:0050840 | extracellular matrix binding | 7.28E-07 | 2.28E-05 | 8 |
| MF | GO:0004866 | endopeptidase inhibitor activity | 1.06E-06 | 3.16E-05 | 14 |
| MF | GO:0001664 | G protein-coupled receptor binding | 1.22E-06 | 3.47E-05 | 17 |
| MF | GO:0017147 | Wnt-protein binding | 1.73E-06 | 4.68E-05 | 6 |
| MF | GO:0005200 | structural constituent of cytoskeleton | 3.22E-06 | 8.32E-05 | 8 |
| MF | GO:0004867 | serine-type endopeptidase inhibitor activity | 8.31E-06 | 0.000206 | 10 |
| MF | GO:0046332 | SMAD binding | 1.54E-05 | 0.000358 | 8 |
| MF | GO:0051787 | misfolded protein binding | 1.57E-05 | 0.000358 | 5 |
| MF | GO:0004175 | endopeptidase activity | 3.25E-05 | 0.000717 | 18 |
| MF | GO:0005125 | cytokine activity | 4.95E-05 | 0.001052 | 12 |
| MF | GO:0030169 | low-density lipoprotein particle binding | 7.50E-05 | 0.001539 | 4 |
| MF | GO:0003779 | actin binding | 9.15E-05 | 0.001749 | 17 |
| MF | GO:0003756 | protein disulfide isomerase activity | 9.41E-05 | 0.001749 | 4 |
| MF | GO:0016864 | intramolecular oxidoreductase activity; transposing S-S bonds | 9.41E-05 | 0.001749 | 4 |
| MF | GO:0038024 | cargo receptor activity | 0.000107 | 0.001925 | 7 |
| MF | GO:0003924 | GTPase activity | 0.000152 | 0.00266 | 13 |
| MF | GO:0051015 | actin filament binding | 0.000172 | 0.002931 | 11 |
| MF | GO:0005516 | calmodulin binding | 0.000217 | 0.003592 | 10 |
| MF | GO:0048306 | calcium-dependent protein binding | 0.000233 | 0.003664 | 7 |
| MF | GO:0016504 | peptidase activator activity | 0.000234 | 0.003664 | 5 |
| MF | GO:0004683 | calmodulin-dependent protein kinase activity | 0.000245 | 0.003737 | 4 |
| MF | GO:0005525 | GTP binding | 0.000309 | 0.004594 | 14 |
| MF | GO:0071813 | lipoprotein particle binding | 0.000453 | 0.006417 | 4 |
| MF | GO:0071814 | protein-lipid complex binding | 0.000453 | 0.006417 | 4 |
| MF | GO:0004222 | metalloendopeptidase activity | 0.000486 | 0.006721 | 7 |
| MF | GO:0043236 | laminin binding | 0.00052 | 0.007032 | 4 |
| MF | GO:0019001 | guanyl nucleotide binding | 0.00057 | 0.007375 | 14 |
| MF | GO:0032561 | guanyl ribonucleotide binding | 0.00057 | 0.007375 | 14 |
| MF | GO:0044548 | S100 protein binding | 0.000717 | 0.009081 | 3 |
| MF | GO:0008237 | metallopeptidase activity | 0.000756 | 0.009367 | 9 |
| MF | GO:0042813 | Wnt receptor activity | 0.000888 | 0.010783 | 3 |
| MF | GO:0016860 | intramolecular oxidoreductase activity | 0.001194 | 0.014214 | 5 |
| MF | GO:0097718 | disordered domain specific binding | 0.001332 | 0.015544 | 4 |
| MF | GO:0008238 | exopeptidase activity | 0.001625 | 0.018598 | 6 |
| MF | GO:0005506 | iron ion binding | 0.001879 | 0.021091 | 8 |
| MF | GO:0008009 | chemokine activity | 0.001961 | 0.02161 | 4 |
| MF | GO:0010314 | phosphatidylinositol-5-phosphate binding | 0.00245 | 0.026028 | 3 |
| MF | GO:0016641 | oxidoreductase activity; acting on the CH-NH2 group of donors; oxygen as acceptor | 0.00245 | 0.026028 | 3 |
| MF | GO:0004197 | cysteine-type endopeptidase activity | 0.00256 | 0.026722 | 6 |
| MF | GO:0019955 | cytokine binding | 0.002993 | 0.030354 | 7 |
| MF | GO:0031625 | ubiquitin protein ligase binding | 0.00301 | 0.030354 | 11 |
| MF | GO:0008528 | G protein-coupled peptide receptor activity | 0.00311 | 0.030708 | 7 |
| MF | GO:0016705 | oxidoreductase activity; acting on paired donors; with incorporation or reduction of molecular oxygen | 0.003172 | 0.030708 | 9 |
| MF | GO:0050431 | transforming growth factor beta binding | 0.0032 | 0.030708 | 3 |
| MF | GO:0042379 | chemokine receptor binding | 0.00335 | 0.03164 | 5 |
| MF | GO:0016638 | oxidoreductase activity; acting on the CH-NH2 group of donors | 0.003622 | 0.033673 | 3 |
| MF | GO:1902936 | phosphatidylinositol bisphosphate binding | 0.004018 | 0.036283 | 6 |
| MF | GO:0001653 | peptide receptor activity | 0.004025 | 0.036283 | 7 |
| MF | GO:0005262 | calcium channel activity | 0.004192 | 0.037227 | 6 |
| MF | GO:0044389 | ubiquitin-like protein ligase binding | 0.004463 | 0.03905 | 11 |
| MF | GO:0001848 | complement binding | 0.005086 | 0.043854 | 3 |
| MF | GO:0042562 | hormone binding | 0.00564 | 0.04794 | 5 |
| MF | GO:0005544 | calcium-dependent phospholipid binding | 0.005728 | 0.048006 | 4 |

P-values were adjusted using the Benjamini–Hochberg method.

# Supplemental Table 5- Differentially expressed protein (p.adj < 0.05) between untreated α-MHC^719/+^ and wildtype mice

| prot.id | log2FC | p.adj |
| --- | --- | --- |
| *Aldh1a1* | 0.641363 | 4.63E-07 |
| *Ankrd1* | 0.610975 | 1.5E-05 |
| *Arhgdib* | 0.697848 | 0.000144 |
| *Ass1* | 1.115443 | 0.000145 |
| *Bdh1* | 0.67222 | 2.09E-13 |
| *Ca3* | 0.983917 | 0.000179 |
| *Capg* | 0.667086 | 0.000208 |
| *Cdk5rap3* | 0.588755 | 0.040116 |
| *Cilp2* | 0.777896 | 0.002109 |
| *Cnn1* | 0.811987 | 8.37E-05 |
| *Col12a1* | 1.109176 | 1.8E-10 |
| *Comp* | 1.100478 | 5.24E-06 |
| *Csrp2* | 0.718675 | 0.049219 |
| *Efhd2* | 0.631119 | 4.09E-05 |
| *Fasn* | 0.676443 | 0.000708 |
| *Fermt3* | 0.694512 | 0.010308 |
| *Ftl1* | 0.626043 | 4.23E-05 |
| *G6pdx* | 0.682085 | 2.48E-06 |
| *Gclc* | 0.64915 | 0.047166 |
| *Gstt1* | 0.65125 | 0.022657 |
| *Hmgcs2* | 0.612537 | 0.002728 |
| *Htatip2* | 0.627684 | 0.007934 |
| *Inmt* | 0.820832 | 0.00105 |
| *Itga8* | 1.165332 | 0.000251 |
| *Itgb3* | 1.634857 | 5.11E-05 |
| *Lmod1* | 1.134744 | 0.008527 |
| *Loxl1* | 0.850106 | 4.95E-08 |
| *Mcpt4* | 1.124836 | 0.004361 |
| *Myh7* | 1.049061 | 0 |
| *Myot* | 0.597332 | 1.23E-13 |
| *Npnt* | 1.031696 | 0.005233 |
| *Phgdh* | 0.916001 | 0.014477 |
| *Pir* | 0.626582 | 0.047247 |
| *Sh3bgrl3* | 0.664741 | 0.042524 |
| *Smtn* | 0.891884 | 4.46E-12 |
| *Sqstm1* | 0.944026 | 0.000231 |
| *Thbs1* | 0.702436 | 2.3E-07 |
| *Thbs4* | 0.799005 | 0.01663 |
| *Tubb1* | 0.809283 | 0.041848 |
| *Uap1l1* | 0.655272 | 9.36E-05 |
| *Xirp2* | 0.667595 | 0 |
| *Art3* | -0.64688 | 1.7E-13 |
| *Ca4* | -0.6217 | 0.00094 |
| *H1-5* | -0.65972 | 1.05E-06 |
| *Mtco1* | -0.59771 | 0.003506 |
| *Mtnd4* | -0.61707 | 1.92E-07 |
| *Ndufa3* | -0.58984 | 0.027805 |
| *Rtn2* | -0.61761 | 0.00094 |
| *Tmem38a* | -0.80818 | 3.41E-05 |
| *Abcb10* | -0.34867 | 9.45E-08 |
| *Abcb7* | -0.28604 | 0.009423 |
| *Abcb8* | -0.20699 | 0.003144 |
| *Abhd14b* | 0.375386 | 0.030616 |
| *Ablim2* | 0.325111 | 0.010889 |
| *Acaa2* | -0.2065 | 0.004393 |
| *Acad10* | 0.245139 | 0.000849 |
| *Acad11* | -0.3186 | 0.009423 |
| *Acox1* | -0.28533 | 0.003147 |
| *Acsf2* | 0.25667 | 0.03063 |
| *Acsl1* | -0.15698 | 0.002472 |
| *Actn1* | 0.275762 | 0.001472 |
| *Actn4* | 0.190655 | 0.011672 |
| *Actr2* | 0.279291 | 0.00534 |
| *Actr3* | 0.327563 | 3.06E-05 |
| *Add1* | 0.304536 | 0.00548 |
| *Adh1* | 0.276716 | 0.045197 |
| *Adss1* | 0.212708 | 0.018505 |
| *Afg3l2* | -0.2211 | 0.002013 |
| *Afm* | 0.247985 | 0.020454 |
| *Agk* | -0.34764 | 0.008071 |
| *Agrn* | 0.206795 | 0.03483 |
| *Ahcy* | 0.274108 | 2.33E-05 |
| *Aifm1* | -0.15997 | 0.011406 |
| *Akap2* | 0.340215 | 0.005191 |
| *Akr1a1* | 0.420391 | 1.99E-06 |
| *Alb* | 0.237278 | 5.79E-07 |
| *Aldh2* | 0.170641 | 0.012223 |
| *Aldh7a1* | 0.384756 | 0.000525 |
| *Anxa1* | 0.50945 | 6.02E-10 |
| *Anxa2* | 0.315486 | 8.36E-08 |
| *Anxa3* | 0.177927 | 0.02233 |
| *Anxa4* | 0.509188 | 4.68E-09 |
| *Anxa5* | 0.22321 | 0.00072 |
| *Ap2a2* | 0.199401 | 0.034052 |
| *Apeh* | 0.261168 | 0.007023 |
| *Apoe* | 0.334027 | 0.004467 |
| *Apool* | -0.29787 | 0.000115 |
| *Appl1* | 0.260107 | 0.007934 |
| *Aprt* | 0.394733 | 0.000994 |
| *Arcn1* | 0.29697 | 0.006274 |
| *Arhgap1* | 0.496157 | 0.000144 |
| *Arhgdia* | 0.267774 | 0.005794 |
| *Arl3* | 0.472053 | 0.031389 |
| *Arpc2* | 0.258837 | 0.029569 |
| *Arpc3* | 0.334061 | 0.005442 |
| *Arpc4* | 0.278194 | 0.024711 |
| *Asah1* | 0.426989 | 0.005049 |
| *Aspn* | 0.3682 | 0.014128 |
| *Atad3* | -0.27222 | 0.000393 |
| *Atic* | 0.367138 | 1.38E-08 |
| *Atp1a1* | -0.39752 | 3.72E-10 |
| *Atp1a2* | -0.4265 | 4.31E-06 |
| *Atp1b1* | -0.46109 | 6.12E-08 |
| *Atp2a2* | -0.36038 | 0 |
| *Atp5mg* | -0.29663 | 0.011672 |
| *Atp5pb* | -0.25222 | 0.009939 |
| *Atp5pd* | -0.40662 | 9.31E-09 |
| *Atp5pf* | -0.38742 | 0.006322 |
| *Atp6v1a* | 0.225692 | 0.021298 |
| *Bag3* | -0.1913 | 0.008494 |
| *Bri3bp* | -0.38974 | 0.03241 |
| *Bsg* | -0.41359 | 2.03E-05 |
| *C3* | 0.173661 | 4.72E-06 |
| *C4b* | 0.201303 | 0.049902 |
| *Cacna2d1* | -0.3363 | 4.55E-06 |
| *Cand1* | 0.233122 | 0.003216 |
| *Cap1* | 0.46242 | 2.33E-06 |
| *Capn1* | 0.281681 | 0.004393 |
| *Capn2* | 0.224925 | 0.012659 |
| *Casq2* | -0.23914 | 0.011672 |
| *Cbr1* | 0.177043 | 0.028574 |
| *Cbr2* | 0.345866 | 0.00791 |
| *Cct2* | 0.193772 | 0.005501 |
| *Cct3* | 0.217174 | 0.000145 |
| *Cct4* | 0.218661 | 0.002881 |
| *Cct5* | 0.191528 | 0.009397 |
| *Cct6a* | 0.207583 | 0.031389 |
| *Cct7* | 0.267125 | 0.000173 |
| *Cct8* | 0.15725 | 0.009063 |
| *Cd36* | -0.23293 | 0.00908 |
| *Cdh13* | -0.26641 | 0.025024 |
| *Ces1d* | 0.18426 | 0.035215 |
| *Chchd3* | -0.27134 | 0.015951 |
| *Ckb* | 0.268637 | 0.011672 |
| *Ckm* | -0.15853 | 0.02277 |
| *Ckmt2* | -0.18189 | 0.026267 |
| *Clic1* | 0.462276 | 5.11E-05 |
| *Clip1* | 0.300813 | 1.83E-06 |
| *Cltc* | 0.154024 | 0.000245 |
| *Clu* | 0.542774 | 1.76E-07 |
| *Cmya5* | -0.18315 | 0.002849 |
| *Col18a1* | 0.496733 | 0.002728 |
| *Col6a1* | 0.280242 | 0.005033 |
| *Col6a2* | 0.317519 | 0.000238 |
| *Col6a6* | 0.386374 | 1.76E-07 |
| *Copa* | 0.197818 | 0.005906 |
| *Copb1* | 0.338821 | 0.004205 |
| *Cops2* | 0.342134 | 0.045502 |
| *Coro1a* | 0.569422 | 0.002819 |
| *Coro1b* | 0.342681 | 0.015984 |
| *Coro1c* | 0.265615 | 0.048685 |
| *Cox4i1* | -0.28277 | 0.004645 |
| *Cox5a* | -0.24567 | 0.024877 |
| *Cox6c* | -0.35651 | 0.043978 |
| *Cp* | 0.203172 | 0.000488 |
| *Cpne1* | 0.353619 | 0.021416 |
| *Cpne3* | 0.409759 | 0.025316 |
| *Cpt2* | -0.16036 | 0.006361 |
| *Csk* | 0.506207 | 0.049922 |
| *Ctnna1* | 0.162382 | 0.004285 |
| *Ctnnb1* | 0.228794 | 0.000464 |
| *Ctps1* | 0.294905 | 0.048488 |
| *Ctsz* | 0.434464 | 0.046597 |
| *Cttn* | 0.273333 | 0.013695 |
| *Cyc1* | -0.36754 | 1.3E-06 |
| *Dbnl* | 0.298209 | 0.013473 |
| *Dctn1* | 0.227945 | 0.000345 |
| *Dctn2* | 0.173143 | 0.027341 |
| *Ddx39b* | 0.353999 | 0.010889 |
| *Des* | 0.221149 | 1.56E-06 |
| *Dlat* | -0.22834 | 0.000305 |
| *Dlst* | -0.24424 | 0.008461 |
| *Dnajb4* | 0.273909 | 0.011406 |
| *Dnm2* | 0.271417 | 0.04273 |
| *Dpysl2* | 0.240889 | 0.000145 |
| *Dpysl3* | 0.374222 | 0.000179 |
| *Dsp* | 0.114453 | 1.05E-06 |
| *Dusp3* | 0.307721 | 0.00791 |
| *Dync1h1* | 0.065409 | 0.015944 |
| *Dysf* | -0.20494 | 2.49E-06 |
| *Eef1a1* | 0.510553 | 0.000927 |
| *Efemp1* | 0.432451 | 0.001163 |
| *Ehd4* | 0.164967 | 0.006361 |
| *Eif4a1* | 0.358263 | 0.000235 |
| *Eif6* | 0.442574 | 0.004621 |
| *Emilin1* | 0.482805 | 6.86E-05 |
| *Epb41* | 0.240006 | 0.040511 |
| *Ephx1* | 0.277426 | 0.019909 |
| *Ephx2* | -0.25896 | 6.37E-05 |
| *Esd* | 0.295862 | 0.004263 |
| *Etfrf1* | -0.38306 | 0.026832 |
| *F13a1* | 0.403172 | 8.41E-06 |
| *Fabp3* | -0.33895 | 0.001443 |
| *Fam162a* | -0.40781 | 0.005397 |
| *Flna* | 0.377874 | 8.56E-11 |
| *Flnb* | 0.131975 | 0.006322 |
| *Flnc* | 0.131055 | 0.000311 |
| *Fmo2* | 0.30816 | 0.020926 |
| *Fmod* | 0.49233 | 0.013965 |
| *Ganab* | 0.256131 | 0.006806 |
| *Gc* | 0.239624 | 0.000561 |
| *Gda* | 0.406628 | 0.001429 |
| *Gdi1* | 0.205586 | 0.011407 |
| *Gfm1* | -0.14604 | 0.015951 |
| *Git1* | 0.419876 | 0.043459 |
| *Gja1* | -0.52767 | 1.08E-05 |
| *Glul* | 0.27732 | 0.02875 |
| *Golga4* | 0.249388 | 0.031818 |
| *Gpi* | 0.159763 | 0.040116 |
| *Gpld1* | 0.283183 | 0.014631 |
| *Gpx1* | 0.317342 | 0.00249 |
| *Gpx3* | 0.446188 | 0.000195 |
| *Gpx4* | 0.275035 | 0.025006 |
| *Gsn* | 0.284144 | 0.001323 |
| *Gsta4* | 0.30896 | 0.000494 |
| *Gstm1* | 0.419642 | 2.08E-07 |
| *Gstm2* | 0.231968 | 0.03241 |
| *Gstm5* | 0.264262 | 0.008236 |
| *H4c16* | -0.37788 | 0.000972 |
| *Hadh* | 0.237634 | 0.041295 |
| *Hadha* | -0.14268 | 0.001025 |
| *Hadhb* | -0.14805 | 0.012732 |
| *Hccs* | -0.27764 | 0.015916 |
| *Hhatl* | -0.40827 | 4.76E-07 |
| *Hk1* | 0.14968 | 0.003031 |
| *Hnrnpf* | 0.307727 | 0.016079 |
| *Hprt1* | 0.341479 | 0.009697 |
| *Hrg* | 0.25183 | 0.011406 |
| *Hsp90aa1* | 0.354765 | 9.31E-09 |
| *Hsp90ab1* | 0.147403 | 0.005866 |
| *Hspa2* | 0.34352 | 0.03241 |
| *Hspa9* | -0.13115 | 0.02966 |
| *Hspb3* | 0.41621 | 0.004393 |
| *Hspd1* | -0.20102 | 7.49E-05 |
| *Hspg2* | 0.070485 | 0.007934 |
| *Hyou1* | 0.231349 | 0.011672 |
| *Iah1* | 0.543555 | 0.014637 |
| *Ide* | 0.229665 | 0.022657 |
| *Idh1* | 0.175682 | 0.015951 |
| *Igbp1* | 0.313959 | 0.03889 |
| *Ighm* | 0.305083 | 0.000614 |
| *Ilk* | 0.280053 | 2.15E-05 |
| *Immt* | -0.16848 | 0.001309 |
| *Itga5* | 0.287854 | 0.045927 |
| *Itih3* | 0.530946 | 0.0053 |
| *Ivns1abp* | 0.557029 | 3.37E-06 |
| *Jph2* | -0.29119 | 0.009794 |
| *Jup* | 0.189815 | 0.002849 |
| *Kank2* | 0.238455 | 0.00623 |
| *Klhl40* | 0.393171 | 0.015951 |
| *Kng1* | 0.214378 | 0.005949 |
| *Ktn1* | -0.23008 | 0.015951 |
| *Lama2* | -0.20199 | 4.33E-11 |
| *Lama5* | 0.181368 | 0.000145 |
| *Lamb1* | -0.30219 | 8.56E-11 |
| *Lamc1* | -0.1957 | 6.91E-07 |
| *Lap3* | 0.184756 | 0.011672 |
| *Lclat1* | -0.37994 | 0.004138 |
| *Lcp1* | 0.551524 | 0.000296 |
| *Ldb3* | -0.18659 | 0.001311 |
| *Letm1* | -0.34822 | 4.94E-06 |
| *Lmna* | 0.347549 | 0 |
| *Lmnb2* | 0.240923 | 0.00534 |
| *Lta4h* | 0.329697 | 4.41E-05 |
| *Ltbp4* | 0.36024 | 0.005353 |
| *Lum* | 0.465326 | 6.04E-06 |
| *Maoa* | 0.449183 | 0.00249 |
| *Maob* | 0.271239 | 0.000123 |
| *Map1b* | 0.361118 | 0.010889 |
| *Mfge8* | 0.437437 | 0.018954 |
| *Mmaa* | -0.23972 | 0.045861 |
| *Mpc1* | -0.3641 | 0.029445 |
| *Mrc1* | 0.218025 | 0.035353 |
| *Mrrf* | -0.26069 | 0.023414 |
| *Msn* | 0.123858 | 0.048909 |
| *mt-Nd2* | -0.53433 | 0.015741 |
| *Mtap* | 0.247574 | 0.029569 |
| *Mtfp1* | -0.24886 | 0.022657 |
| *Mthfd1* | 0.178425 | 0.011148 |
| *Mtnd1* | -0.49441 | 0.001419 |
| *Mtnd5* | -0.46523 | 1.92E-05 |
| *Mvp* | 0.422188 | 8.56E-11 |
| *Mybpc3* | -0.12892 | 0.002013 |
| *Mybphl* | 0.381576 | 0.024711 |
| *Myh10* | 0.24341 | 0.004393 |
| *Myh11* | 0.308507 | 5.41E-08 |
| *Myh14* | 0.185684 | 0.003841 |
| *Myh6* | -0.14142 | 0.002079 |
| *Myh9* | 0.234644 | 9.06E-09 |
| *Myl3* | -0.24771 | 0.004388 |
| *Mylk3* | -0.28396 | 0.011672 |
| *Myof* | 0.523726 | 0.023459 |
| *Myom1* | -0.11745 | 0.000867 |
| *Myoz2* | -0.24337 | 0.003026 |
| *Naca* | -0.30884 | 0 |
| *Nap1l4* | 0.22146 | 0.020135 |
| *Napa* | 0.31206 | 0.003432 |
| *Naprt* | 0.361687 | 0.019735 |
| *Naxe* | 0.251608 | 0.036363 |
| *Ndufa13* | -0.37163 | 1.23E-05 |
| *Ndufa8* | -0.26335 | 0.007934 |
| *Ndufa9* | -0.28068 | 4.95E-08 |
| *Ndufab1* | -0.36958 | 0.006345 |
| *Ndufaf4* | -0.33725 | 0.010003 |
| *Ndufb1* | -0.37026 | 0.023414 |
| *Ndufb10* | -0.24159 | 0.004235 |
| *Ndufb11* | -0.26852 | 0.013194 |
| *Ndufb4* | -0.25338 | 0.046597 |
| *Ndufb5* | -0.35398 | 0.002832 |
| *Ndufb6* | -0.46092 | 0.009089 |
| *Ndufb8* | -0.42623 | 4.3E-05 |
| *Ndufs1* | -0.1837 | 0.000323 |
| *Ndufs2* | -0.15604 | 0.02094 |
| *Ndufs4* | -0.5439 | 8.16E-06 |
| *Ndufs8* | -0.2932 | 0.009423 |
| *Ndufv2* | -0.25212 | 0.019926 |
| *Nebl* | -0.21553 | 6.18E-05 |
| *Nes* | 0.525688 | 0.00268 |
| *Nid2* | 0.224301 | 0.000195 |
| *Nnt* | -0.19578 | 2.81E-05 |
| *Nqo1* | 0.40015 | 8.62E-06 |
| *Nrap* | 0.393556 | 0 |
| *Nsf* | 0.233222 | 0.047231 |
| *Nt5c* | 0.573449 | 0.008744 |
| *Obscn* | 0.132557 | 2.43E-10 |
| *Ociad1* | -0.30858 | 0.01052 |
| *Oxct1* | -0.20201 | 0.01702 |
| *P4hb* | 0.254226 | 9.96E-05 |
| *Pafah1b1* | 0.231462 | 0.019735 |
| *Palld* | -0.16046 | 0.04355 |
| *Papln* | 0.325642 | 0.00288 |
| *Pc* | 0.146572 | 0.020669 |
| *Pdhb* | -0.18206 | 0.009939 |
| *Pdhx* | -0.22186 | 0.03063 |
| *Pdia3* | 0.274601 | 3.07E-06 |
| *Pdia4* | 0.302492 | 0.000525 |
| *Pdk1* | 0.206274 | 0.030316 |
| *Pdlim7* | 0.480666 | 0.005349 |
| *Pdp1* | 0.229469 | 0.013129 |
| *Pepd* | 0.270023 | 0.014549 |
| *Pfkp* | 0.394035 | 0.000532 |
| *Pfn1* | 0.237045 | 0.020454 |
| *Pgam1* | 0.31048 | 0.001163 |
| *Pgd* | 0.2839 | 9.58E-05 |
| *Pgm1* | -0.14521 | 0.015951 |
| *Pgm5* | 0.42317 | 0 |
| *Phb1* | -0.2211 | 0.000896 |
| *Phb2* | -0.28668 | 1.83E-06 |
| *Plg* | 0.25981 | 1.92E-05 |
| *Plin2* | -0.36095 | 0.029569 |
| *Plin3* | -0.3282 | 0.000348 |
| *Plin5* | -0.39356 | 7.09E-05 |
| *Pls3* | 0.448191 | 2.03E-05 |
| *Pnp* | 0.250331 | 0.015382 |
| *Postn* | 0.137878 | 0.024617 |
| *Ppia* | 0.205823 | 0.031389 |
| *Ppp1r12a* | 0.248951 | 0.044867 |
| *Ppp1r3a* | -0.44366 | 1.62E-07 |
| *Psma1* | 0.281147 | 0.008945 |
| *Psma3* | 0.295284 | 0.038411 |
| *Psma6* | 0.322475 | 0.002906 |
| *Psmb4* | 0.333085 | 0.003238 |
| *Psmc2* | 0.200993 | 0.004036 |
| *Psmc3* | 0.156789 | 0.029812 |
| *Psmc6* | 0.211121 | 0.012691 |
| *Psmd13* | 0.19801 | 0.009026 |
| *Psmd14* | 0.259121 | 0.034625 |
| *Ptbp1* | 0.356409 | 0.043459 |
| *Pter* | 0.358184 | 0.005936 |
| *Ptgds* | 0.491725 | 0.034773 |
| *Ptgis* | 0.292695 | 0.004393 |
| *Pzp* | 0.125425 | 0.014393 |
| *Qsox1* | 0.42235 | 0.014267 |
| *Rack1* | 0.185489 | 0.02691 |
| *Rcc1* | 0.318089 | 0.036157 |
| *Rnh1* | 0.253692 | 0.000166 |
| *Rpl13* | -0.33524 | 0.016506 |
| *Rpl13a* | -0.32905 | 0.007851 |
| *Rpl18* | -0.28439 | 0.02691 |
| *Rpl23* | -0.29975 | 0.046514 |
| *Rpl24* | -0.51064 | 0.007479 |
| *Rpl4* | -0.19096 | 0.004036 |
| *Rpl7* | -0.1995 | 0.011902 |
| *Rpl7a* | -0.32304 | 0.003841 |
| *Rps24* | -0.42795 | 0.029713 |
| *Rps26* | -0.49174 | 0.030673 |
| *Rsu1* | 0.216874 | 0.030851 |
| *Rtn4* | 0.30897 | 0.000573 |
| *Ruvbl1* | 0.356257 | 0.018156 |
| *Ryr2* | -0.44493 | 0 |
| *Samm50* | -0.28348 | 0.000151 |
| *Sars1* | 0.299427 | 0.015951 |
| *Sccpdh* | -0.24674 | 0.016128 |
| *Sdhc* | -0.38409 | 0.015951 |
| *Sec23a* | 0.35746 | 0.000323 |
| *Septin2* | 0.350192 | 0.000813 |
| *Septin7* | 0.250354 | 0.043314 |
| *Septin9* | 0.356455 | 0.001438 |
| *Serbp1* | -0.37266 | 0.003238 |
| *Serpinb6* | 0.306861 | 2.14E-05 |
| *Serpinf2* | 0.249064 | 0.035399 |
| *Sfpq* | -0.19595 | 0.025727 |
| *Sgca* | -0.18874 | 0.044541 |
| *Sh3bgrl* | 0.320167 | 0.046514 |
| *Shmt1* | 0.482841 | 0.034052 |
| *Slc12a7* | -0.27892 | 0.005906 |
| *Slc16a1* | -0.41352 | 0.003205 |
| *Slc25a11* | -0.24026 | 0.000472 |
| *Slc25a12* | -0.28993 | 5.79E-10 |
| *Slc25a13* | -0.33828 | 8.56E-11 |
| *Slc25a20* | -0.25318 | 0.001252 |
| *Slc25a3* | -0.27941 | 0.001438 |
| *Slc25a4* | -0.26262 | 0.003173 |
| *Slc25a42* | -0.43622 | 0.001613 |
| *Slc8a1* | -0.23107 | 0.005493 |
| *Snd1* | 0.29371 | 1.73E-06 |
| *Sntb2* | 0.379281 | 0.047615 |
| *Snx1* | 0.450052 | 0.005618 |
| *Snx2* | 0.321108 | 0.004326 |
| *Snx6* | 0.482976 | 0.036534 |
| *Sord* | 0.434786 | 9.45E-05 |
| *Speg* | -0.1182 | 0.009794 |
| *Spg7* | -0.27925 | 0.006221 |
| *Spr* | 0.214156 | 0.002969 |
| *Sptan1* | 0.065553 | 0.019735 |
| *Sptb* | 0.155877 | 0.000193 |
| *Sqor* | 0.213389 | 0.006649 |
| *Srl* | -0.25961 | 8.8E-08 |
| *Stat3* | 0.285417 | 0.015382 |
| *Sucla2* | -0.14856 | 0.017491 |
| *Suclg2* | 0.278591 | 2.14E-05 |
| *Svil* | 0.205629 | 0.000145 |
| *Synpo2l* | 0.161017 | 0.007319 |
| *Tagln2* | 0.36082 | 0.000119 |
| *Taldo1* | 0.23782 | 0.004277 |
| *Tcap* | -0.28969 | 0.004369 |
| *Tcp1* | 0.280023 | 0.000311 |
| *Tecrl* | -0.481 | 0.00249 |
| *Tf* | 0.142288 | 0.001477 |
| *Tfam* | -0.28694 | 0.047495 |
| *Tfrc* | -0.28264 | 0.003216 |
| *Tgfbi* | 0.263118 | 0.012691 |
| *Tkt* | 0.389868 | 7.43E-07 |
| *Tln1* | 0.396077 | 0 |
| *Tmem143* | -0.31226 | 0.008882 |
| *Tmem65* | -0.41322 | 4.87E-05 |
| *Tmod1* | -0.20248 | 0.011207 |
| *Tmod4* | 0.521149 | 0.012834 |
| *Tnni3* | -0.29749 | 0.002969 |
| *Tnnt2* | -0.38744 | 1.49E-06 |
| *Tomm5* | -0.33076 | 0.047741 |
| *Tpm1* | -0.32407 | 0.005278 |
| *Tpm2* | 0.327186 | 0.03496 |
| *Trim28* | 0.375318 | 0.048909 |
| *Trnt1* | 0.37488 | 0.027244 |
| *Ttn* | -0.19108 | 0 |
| *Tubb5* | 0.42717 | 0.011207 |
| *Tufm* | -0.13964 | 0.03241 |
| *Txndc5* | 0.486642 | 0.013129 |
| *Txnl1* | 0.217509 | 0.029241 |
| *Txnrd1* | 0.229373 | 0.026885 |
| *Uchl1* | 0.366925 | 0.040511 |
| *Uchl5* | 0.368217 | 0.013194 |
| *Ucp3* | -0.26593 | 0.015246 |
| *Ugdh* | 0.527618 | 0.03241 |
| *Uqcr10* | -0.32726 | 0.048488 |
| *Uqcrb* | -0.28124 | 0.00288 |
| *Uqcrc2* | -0.17264 | 0.008527 |
| *Uqcrfs1* | -0.30139 | 0.000386 |
| *Uqcrh* | -0.31696 | 0.031837 |
| *Uqcrq* | -0.37025 | 0.005442 |
| *Usp14* | 0.243445 | 0.001735 |
| *Vat1* | 0.544142 | 1.96E-07 |
| *Vcan* | 0.453737 | 0.000855 |
| *Vcl* | 0.098089 | 0.007907 |
| *Vcp* | 0.147392 | 0.000313 |
| *Vim* | 0.398175 | 4.76E-07 |
| *Vps26b* | 0.361368 | 0.046551 |
| *Vps29* | 0.327324 | 0.038907 |
| *Vtn* | 0.46869 | 0.000207 |
| *Vwa5a* | 0.312045 | 0.000405 |
| *Vwf* | 0.477508 | 0.000994 |
| *Wdr1* | 0.187928 | 0.031379 |
| *Wfs1* | -0.34507 | 0.012291 |
| *Xirp1* | 0.150821 | 0.004467 |
| *Ywhaz* | 0.234368 | 0.031837 |
| *Zyx* | 0.318937 | 0.022024 |

P-values were adjusted using the Benjamini–Hochberg method.

# Supplemental Table 6- Differentially expressed protein (p.adj < 0.05)between α-MHC^719/+^ treated with pio and wildtype mice

| prot.id | log2FC | p.adj |  |  |  |
| --- | --- | --- | --- | --- | --- |
| *Aspn* | 1.056665 | 0.008267909 |  |  |  |
| *Bdh1* | 1.0193 | 6.04195E-10 |  |  |  |
| *Fth1* | 0.979406 | 6.11622E-12 |  |  |  |
| *Myh7* | 0.875244 | 0 |  |  |  |
| *Hadh* | 0.828754 | 4.48057E-10 |  |  |  |
| *Dcn* | 0.635528 | 0.032368294 |  |  |  |
| *Serpinc1* | 0.604521 | 0.000540663 |  |  |  |
| *Lum* | 0.565103 | 0.022013436 |  |  |  |
| *Sdr39u1* | 0.508448 | 9.04852E-05 |  |  |  |
| *Acad10* | 0.490921 | 1.675E-06 |  |  |  |
| *Prosc* | 0.4508 | 0.009683682 |  |  |  |
| *Sqrdl* | 0.418215 | 0.013763113 |  |  |  |
| *Uchl3;Uchl4* | 0.399208 | 0.01603293 |  |  |  |
| *Mgll* | 0.359197 | 0.034785408 |  |  |  |
| *Suclg2* | 0.344731 | 3.585E-07 |  |  |  |
| *Ugp2* | 0.342869 | 0.000380608 |  |  |  |
| *Serpina1c;Serpina1a* | 0.336656 | 0.000183745 |  |  |  |
| *Aldh4a1* | 0.314543 | 0.017401822 |  |  |  |
| *Hpx* | 0.31251 | 0.049979793 |  |  |  |
| *Fabp4* | 0.263038 | 0.01901621 |  |  |  |
| *Sirt5* | 0.258302 | 0.049979793 |  |  |  |
| *Vim* | 0.236277 | 0.032368294 |  |  |  |
| *Dhrs4* | 0.23606 | 0.039089346 |  |  |  |
| *Idh2* | 0.214062 | 0.000265369 |  |  |  |
| *Fbn1* | 0.210553 | 0.003644296 |  |  |  |
| *Nnt* | 0.166132 | 0.000970137 |  |  |  |
| *Aco2* | 0.129769 | 0.017972377 |  |  |  |
| *Ttn* | -0.02687 | 0.004588595 |  |  |  |
| *Atp2a2* | -0.1632 | 6.87249E-05 |  |  |  |
| *Ryr2* | -0.17535 | 1.44874E-09 |  |  |  |
| *Gdi2* | -0.21289 | 0.014735288 |  |  |  |
| *Ehd4* | -0.24348 | 0.000865835 |  |  |  |
| *Pgls* | -0.28187 | 0.038378761 |  |  |  |
| *Ehd1* | -0.29033 | 6.87249E-05 |  |  |  |
| *Glo1* | -0.35037 | 9.19411E-05 |  |  |  |
| *Hbb-b1* | -0.37579 | 1.25916E-05 |  |  |  |
| *Gja1* | -0.40316 | 0.022013436 |  |  |  |
| *Slc4a1* | -0.43121 | 0.033736691 |  |  |  |
| *Hbb-b2* | -0.49809 | 0.00024642 |  |  |  |
| *Ca2* | -0.57773 | 8.47877E-09 |  |  |  |
| *Art3* | -0.66904 | 0.004224491 |  |  |  |
| *Tfrc* | -0.71776 | 0.018242197 |  |  |  |
| *Aldh1b1* | -0.89427 | 0.002243823 |  |  |  |
| *Cox8b* | -0.97569 | 0.005249495 |  |  |  |
| *Pln* | -1.25449 | 0.004224491 |  |  |  |
| *Nppa* | -1.61449 | 0.027379631 |  |  |  |

P-values were adjusted using the Benjamini–Hochberg method.

# Supplemental Table 7- Differentially expressed protein (p.adj < 0.05)between α-MHC^719/+^ treated with R-pio and untreated wildtype mice

| prot.id | log2FC | p.adj |
| --- | --- | --- |
| *Efhd2* | 0.608047 | 5.90E-05 |
| *Hadh* | 0.590741 | 1.84E-07 |
| *Ivns1abp* | 0.698655 | 8.28E-11 |
| *Myh7* | 0.592539 | 0 |
| *Nln* | 0.634812 | 0.018507 |
| *Ptgds* | 0.736542 | 0.00235 |
| *S100a6* | 0.609024 | 0.040414 |
| *Shmt1* | 0.60852 | 0.008746 |
| *Sparc* | 1.028295 | 4.46E-04 |
| *Thbs4* | 0.9321 | 9.84E-04 |
| *Xirp2* | 0.764136 | 0 |
| *Aoc3* | -0.79004 | 1.63E-08 |
| *Aqp1* | -0.59009 | 5.25E-04 |
| *Art3* | -0.6268 | 2.04E-11 |
| *Atp6v1d* | -1.21243 | 0.038091 |
| *Ca3* | -0.83382 | 0.001354 |
| *Cilp2* | -1.3016 | 1.77E-12 |
| *Col1a1* | -0.6115 | 0.010001 |
| *Eln* | -0.99579 | 1.61E-07 |
| *Fbln5* | -0.67978 | 0.002097 |
| *Krt10* | -0.7055 | 0.00304 |
| *Loxl1* | -1.01754 | 5.02E-04 |
| *Myh11* | -0.85912 | 0 |
| *Ogn* | -0.60693 | 8.46E-05 |
| *Vwf* | -0.6215 | 0.005896 |
| *Abcb10* | -0.29341 | 1.35E-06 |
| *Abcb7* | -0.31402 | 2.64E-04 |
| *Abcb8* | -0.17053 | 0.010001 |
| *Acaa2* | -0.19443 | 8.12E-04 |
| *Acad8* | 0.142346 | 0.031641 |
| *Acox1* | -0.33299 | 8.94E-05 |
| *Actr3* | 0.164003 | 0.038091 |
| *Adss1* | 0.202961 | 0.024949 |
| *Afg3l2* | -0.15424 | 0.03928 |
| *Agk* | -0.24547 | 0.035815 |
| *Ahsa1* | 0.286642 | 0.008835 |
| *Aifm1* | -0.17705 | 0.003488 |
| *Akap2* | 0.354199 | 0.00568 |
| *Akr1a1* | 0.286142 | 6.36E-04 |
| *Alb* | 0.101052 | 0.032015 |
| *Ankrd1* | 0.58018 | 5.34E-04 |
| *Anpep* | -0.30446 | 0.03928 |
| *Anxa1* | 0.302146 | 7.63E-05 |
| *Anxa4* | 0.245741 | 0.004294 |
| *Anxa5* | 0.244983 | 2.13E-04 |
| *Apool* | -0.22584 | 0.002423 |
| *Arcn1* | 0.223432 | 0.021282 |
| *Asph* | -0.23722 | 0.001359 |
| *Atic* | 0.178644 | 0.006504 |
| *Atp1a1* | -0.48708 | 0 |
| *Atp1a2* | -0.28232 | 0.001146 |
| *Atp1b1* | -0.40005 | 1.00E-05 |
| *Atp2a2* | -0.38115 | 0 |
| *Atp5mg* | -0.26456 | 0.040164 |
| *Atp5pb* | -0.26131 | 0.00568 |
| *Atp5pd* | -0.20659 | 0.008503 |
| *Bag2* | 0.25408 | 0.039991 |
| *Bcam* | -0.19137 | 0.038091 |
| *Bdh1* | 0.292894 | 0.031075 |
| *Bsg* | -0.36046 | 1.77E-04 |
| *Cacna2d1* | -0.42289 | 2.00E-10 |
| *Capg* | 0.500144 | 0.010001 |
| *Cct2* | 0.201998 | 0.002119 |
| *Cct3* | 0.237862 | 3.74E-06 |
| *Cct4* | 0.164787 | 0.025802 |
| *Cct5* | 0.189258 | 0.006498 |
| *Cct6a* | 0.226074 | 0.03928 |
| *Cct7* | 0.21778 | 0.002707 |
| *Cct8* | 0.206208 | 5.32E-05 |
| *Cd36* | -0.20675 | 0.023124 |
| *Cdh13* | -0.40622 | 5.89E-05 |
| *Cfl2* | 0.300404 | 0.019879 |
| *Clic1* | 0.348369 | 0.001441 |
| *Clip1* | 0.257357 | 1.25E-05 |
| *Cltc* | 0.091034 | 0.018918 |
| *Clu* | 0.491063 | 6.42E-10 |
| *Clybl* | 0.141374 | 0.031497 |
| *Cox4i1* | -0.22263 | 0.03928 |
| *Cryab* | 0.228336 | 0.017512 |
| *Ctnna1* | 0.15427 | 0.003347 |
| *Ctps1* | 0.260472 | 0.044052 |
| *Cyc1* | -0.23617 | 0.003309 |
| *Dars1* | 0.173693 | 0.004294 |
| *Ddah2* | 0.257287 | 0.012714 |
| *Ddx1* | 0.160607 | 0.035238 |
| *Des* | 0.338696 | 0 |
| *Dnaja4* | 0.273707 | 0.003372 |
| *Dnajb4* | 0.316619 | 0.004191 |
| *Dsp* | 0.089945 | 6.24E-05 |
| *Dusp3* | 0.246195 | 0.042475 |
| *Dysf* | -0.13241 | 0.002797 |
| *Eef2* | 0.090676 | 0.044052 |
| *Ehbp1l1* | 0.216318 | 0.03846 |
| *Ehd4* | 0.144937 | 0.021892 |
| *Eif4a1* | 0.260944 | 0.00888 |
| *Ephx2* | -0.17287 | 0.020825 |
| *Ezr* | -0.34598 | 0.020596 |
| *Fabp4* | 0.202209 | 0.038597 |
| *Fam162a* | -0.32876 | 0.011143 |
| *Fbn1* | -0.45049 | 4.32E-11 |
| *Fga* | -0.32034 | 2.64E-04 |
| *Fgb* | -0.27349 | 9.13E-04 |
| *Fgg* | -0.32243 | 1.58E-04 |
| *Fhl1* | 0.368388 | 4.94E-04 |
| *Flna* | -0.56113 | 4.37E-12 |
| *Flnc* | 0.289771 | 0 |
| *Fmo2* | -0.57517 | 0.001345 |
| *Fscn1* | 0.341979 | 0.029287 |
| *Ftl1* | 0.324705 | 0.015306 |
| *Gcsh* | 0.449023 | 0.036635 |
| *Gimap4* | 0.203139 | 0.038597 |
| *Gja1* | -0.51741 | 8.34E-05 |
| *Glul* | 0.331663 | 0.001339 |
| *Gnas* | -0.24997 | 0.007738 |
| *Gpi* | 0.150585 | 0.04567 |
| *Gpx4* | 0.357875 | 0.005238 |
| *Gsta4* | 0.278378 | 0.004167 |
| *H1-0* | -0.32902 | 0.03928 |
| *H1-5* | -0.32688 | 0.027378 |
| *Hadha* | -0.13716 | 0.002707 |
| *Hhatl* | -0.34004 | 9.55E-05 |
| *Hk1* | 0.209215 | 2.22E-06 |
| *Hprt1* | 0.305352 | 0.022361 |
| *Hsp90aa1* | 0.283154 | 2.67E-06 |
| *Hsp90ab1* | 0.162375 | 0.002119 |
| *Hspa4* | 0.130807 | 0.036635 |
| *Hspb1* | 0.432375 | 2.39E-07 |
| *Hspb2* | 0.287791 | 0.025806 |
| *Hspb3* | 0.386902 | 0.010001 |
| *Hspb6* | 0.255063 | 0.009185 |
| *Hspb7* | 0.308736 | 0.005896 |
| *Hsph1* | 0.259275 | 0.015031 |
| *Htatip2* | 0.57409 | 0.008229 |
| *Iah1* | 0.513963 | 0.043014 |
| *Ighg1* | -0.58051 | 3.17E-04 |
| *Immt* | -0.1605 | 0.00299 |
| *Itga7* | -0.20412 | 0.042216 |
| *Jph2* | -0.34007 | 0.001345 |
| *Klhl40* | 0.450768 | 0.005167 |
| *Klhl41* | 0.232103 | 0.002182 |
| *Lama2* | -0.19846 | 9.45E-12 |
| *Lamb1* | -0.20142 | 4.43E-06 |
| *Lamb2* | -0.15365 | 0.007551 |
| *Lamc1* | -0.21701 | 1.61E-07 |
| *Lap3* | 0.179258 | 0.006201 |
| *Lclat1* | -0.32633 | 0.007738 |
| *Letm1* | -0.37029 | 1.94E-08 |
| *Lmna* | 0.197841 | 2.08E-06 |
| *Lmnb1* | -0.18887 | 0.009927 |
| *Lonp1* | 0.125131 | 0.008722 |
| *Lpl* | -0.26192 | 0.00716 |
| *Ltbp4* | -0.35721 | 0.001787 |
| *Maob* | -0.31402 | 4.12E-04 |
| *Map3k20* | 0.275481 | 0.015977 |
| *Mif* | 0.379014 | 0.012714 |
| *Mmut* | -0.18108 | 0.018495 |
| *Mtap* | 0.217114 | 0.03928 |
| *Mtnd4* | -0.31197 | 0.014703 |
| *Mtnd5* | -0.26881 | 0.033884 |
| *Mvp* | 0.438539 | 5.58E-14 |
| *Mylk* | -0.5247 | 0.002108 |
| *Myot* | 0.413805 | 1.47E-06 |
| *Myoz2* | -0.1946 | 0.036139 |
| *Naca* | -0.10617 | 0.001213 |
| *Nceh1* | -0.22551 | 0.007396 |
| *Ndufa13* | -0.30328 | 1.31E-04 |
| *Ndufa9* | -0.15178 | 0.013371 |
| *Ndufb1* | -0.30398 | 0.042216 |
| *Ndufb11* | -0.26323 | 0.008629 |
| *Ndufb5* | -0.26124 | 0.028445 |
| *Ndufb8* | -0.3382 | 0.002886 |
| *Ndufc2* | -0.23731 | 0.03928 |
| *Nebl* | -0.16338 | 0.004249 |
| *Nes* | 0.552135 | 0.002052 |
| *Nexn* | 0.191557 | 0.034434 |
| *Nnt* | -0.20844 | 9.80E-07 |
| *Nol3* | 0.291789 | 0.006504 |
| *Nqo1* | 0.24781 | 0.006067 |
| *Nrap* | 0.521359 | 0 |
| *Nrp1* | -0.31627 | 0.038597 |
| *Oat* | 0.162434 | 0.018839 |
| *Obscn* | 0.176097 | 0 |
| *Ogdh* | 0.117778 | 0.00205 |
| *Pa2g4* | 0.239399 | 0.002655 |
| *Pam* | -0.53164 | 0.001339 |
| *Pdia3* | 0.168993 | 0.001809 |
| *Pdia4* | 0.221732 | 0.010717 |
| *Pdxdc1* | 0.553555 | 0.036623 |
| *Pgd* | 0.19755 | 0.021282 |
| *Pgm5* | 0.318005 | 2.24E-08 |
| *Phb2* | -0.17076 | 0.00456 |
| *Plec* | 0.076406 | 8.34E-05 |
| *Plg* | 0.14799 | 0.011508 |
| *Plin3* | -0.20636 | 0.039673 |
| *Plin5* | -0.27274 | 0.008031 |
| *Pls3* | 0.242361 | 0.040237 |
| *Postn* | -0.32506 | 3.87E-04 |
| *Ppp1r3a* | -0.31914 | 3.40E-04 |
| *Ppp2r1a* | 0.190274 | 0.00262 |
| *Prxl2b* | 0.278876 | 0.008031 |
| *Psma6* | 0.269127 | 0.00888 |
| *Psmc2* | 0.156891 | 0.039693 |
| *Psmc3* | 0.179282 | 0.006498 |
| *Psmc6* | 0.181136 | 0.01913 |
| *Psmd13* | 0.205849 | 0.008835 |
| *Psmd2* | 0.139374 | 0.04909 |
| *Pter* | 0.286661 | 0.03928 |
| *Ptges3* | 0.387604 | 0.042216 |
| *Ptgis* | -0.45483 | 0.007738 |
| *Pygb* | -0.1245 | 0.04849 |
| *Rack1* | 0.191369 | 0.009716 |
| *Rdx* | -0.23938 | 0.033852 |
| *Rnh1* | 0.272131 | 2.69E-06 |
| *Rpsa* | 0.198804 | 0.035351 |
| *Rtn2* | -0.54019 | 0.005235 |
| *Rtn4* | 0.325879 | 4.51E-04 |
| *Ryr2* | -0.39159 | 0 |
| *Sars1* | 0.243316 | 0.039673 |
| *Septin2* | 0.253145 | 0.016094 |
| *Septin9* | 0.263835 | 0.044313 |
| *Serpinb6* | 0.243808 | 0.004661 |
| *Serpinc1* | -0.40611 | 3.13E-04 |
| *Serpinh1* | 0.216967 | 0.009991 |
| *Sgca* | -0.20652 | 0.0114 |
| *Slc12a7* | -0.17912 | 0.045025 |
| *Slc25a11* | -0.19108 | 0.006404 |
| *Slc25a12* | -0.24463 | 5.51E-08 |
| *Slc25a13* | -0.26364 | 3.19E-07 |
| *Slc25a3* | -0.24662 | 8.12E-04 |
| *Slc25a42* | -0.34122 | 0.029746 |
| *Slc27a1* | -0.34422 | 0.006504 |
| *Slc2a4* | -0.35385 | 0.03928 |
| *Slc4a1* | -0.46629 | 6.73E-05 |
| *Slc8a1* | -0.20117 | 0.035351 |
| *Sorbs2* | 0.203202 | 5.69E-05 |
| *Spg7* | -0.22791 | 0.014001 |
| *Spr* | 0.144193 | 0.041253 |
| *Srl* | -0.2015 | 7.04E-05 |
| *Stom* | -0.28554 | 0.018839 |
| *Svil* | 0.243751 | 2.56E-05 |
| *Synpo2l* | 0.398357 | 2.57E-13 |
| *Tcp1* | 0.221645 | 0.00345 |
| *Tecrl* | -0.53819 | 0.004076 |
| *Thbs1* | -0.53336 | 9.84E-04 |
| *Tmem65* | -0.44018 | 8.54E-06 |
| *Tmod1* | -0.20004 | 0.014218 |
| *Tmod4* | 0.583943 | 0.010001 |
| *Tmpo* | -0.24485 | 0.004144 |
| *Tnnt2* | -0.21605 | 0.019358 |
| *Trim72* | 0.220571 | 2.08E-04 |
| *Ttn* | -0.09582 | 0 |
| *Txnl1* | 0.247883 | 0.005235 |
| *Uap1* | 0.372501 | 0.034436 |
| *Uap1l1* | 0.3821 | 0.011603 |
| *Uchl1* | 0.503077 | 8.34E-05 |
| *Ugp2* | 0.153949 | 0.006862 |
| *Vat1* | 0.259077 | 0.027576 |
| *Vcp* | 0.112636 | 0.013079 |
| *Vdac1* | -0.13367 | 0.035139 |
| *Vwa5a* | 0.240081 | 0.004456 |
| *Wdr1* | 0.212259 | 0.009927 |
| *Xirp1* | 0.318212 | 1.34E-11 |
| *Yars1* | 0.185342 | 0.042151 |
| *Ywhah* | 0.259594 | 0.01577 |

P-values were adjusted using the Benjamini–Hochberg method.
